# Comprehensive Analysis of Arabinogalactan Protein-Encoding Genes Reveals the Involvement of Three *BrFLA* Genes in Pollen Germination in *Brassica rapa*

**DOI:** 10.3390/ijms222313142

**Published:** 2021-12-05

**Authors:** Huiting Huang, Yingjing Miao, Yuting Zhang, Li Huang, Jiashu Cao, Sue Lin

**Affiliations:** 1 Institute of Life Sciences, College of Life and Environmental Science, Wenzhou University, Wenzhou 325000, China; 20451334008@stu.wzu.edu.cn (H.H.); yjmiao@zju.edu.cn (Y.M.); 20461337006@stu.wzu.edu.cn (Y.Z.); 2 Laboratory of Cell & Molecular Biology, Institute of Vegetable Science, Zhejiang University, Hangzhou 310058, China; lihuang@zju.edu.cn; 3 Biomedicine Collaborative Innovation Center of Zhejiang Province, Wenzhou University, Wenzhou 325000, China

**Keywords:** Chinese cabbage (*Brassica rapa* ssp. *chinensis*), arabinogalactan protein (AGP), fasciclin-like AGP (FLA), male sterility, pollen germination

## Abstract

Arabinogalactan proteins (AGPs) are a superfamily of hydroxyproline-rich glycoproteins that are massively glycosylated, widely implicated in plant growth and development. No comprehensive analysis of the *AGP* gene family has been performed in Chinese cabbage (*Brassica rapa* ssp. *chinensis*). Here, we identified a total of 293 putative AGP-encoding genes in *B. rapa*, including 25 classical AGPs, three lysine-rich AGPs, 30 AG-peptides, 36 fasciclin-like AGPs (FLAs), 59 phytocyanin-like AGPs, 33 xylogen-like AGPs, 102 other chimeric AGPs, two non-classical AGPs and three AGP/extensin hybrids. Their protein structures, phylogenetic relationships, chromosomal location and gene duplication status were comprehensively analyzed. Based on RNA sequencing data, we found that 73 *AGP* genes were differentially expressed in the floral buds of the sterile and fertile plants at least at one developmental stage in *B. rapa*, suggesting a potential role of AGPs in male reproductive development. We further characterized *BrFLA2*, *BrFLA28* and *BrFLA32*, three FLA members especially expressed in anthers, pollen grains and pollen tubes. *BrFLA2*, *BrFLA28* and *BrFLA32* are indispensable for the proper timing of pollen germination under high relative humidity. Our study greatly extends the repertoire of AGPs in *B. rapa* and reveals a role for three members of the FLA subfamily in pollen germination.

## 1. Introduction

Arabinogalactan proteins (AGPs) are a class of hydroxyproline-rich proteoglycan compounds that are widely present in higher plants [1]. AGPs consist of variable core protein backbones covered by carbohydrate side chains rich in arabinose and galactose, with a molecular mass of about 60–300 kDa. For most of the AGPs, the core protein structure occupies less than 10% of the mature molecule, while more than 90% is related to the carbohydrate side chains [1,2]. The complexity arising from the incredible diversity of the glycans decorating the protein backbone makes AGPs a large complex family in higher plants. To date, a total of 85 and 282 putative AGPs have been identified in the model plant *Arabidopsis thaliana* (hereafter, Arabidopsis) and rice, respectively [3,4,5,6,7,8,9,10,11].

According to the domain constitutions of the core proteins, AGPs are divided into classical AGPs, AG-peptides, lysine (Lys)-rich AGPs, chimeric AGPs (CAGPs) and non-classical AGPs [10]. Classical AGPs usually consist of (i) an N-terminal signal peptide sequence, (ii) a central domain with variable length and rich in proline (Pro), alanine (Ala), serine (Ser) and threonine (Thr) (PAST) residues, and (iii) a C-terminal glycosylphosphatidylinositol (GPI)-anchored signal [12]. One unique type of classical AGP with PAST-rich domains inserted by Lys-rich regions is defined as Lys-rich AGPs [13]. The other kind of classical AGP, namely AG-peptides, only has 10–13 amino acid residues in short protein backbones [12]. CAGPs could be further classified into several subfamilies based on other different conservative domains, such as fasciclin-like AGPs (FLAs), xylogen-like AGPs (XYLPs) and phytocyanin-like AGPs (PLAs; also named as early nodulin-like proteins, ENODLs) [6,7,8,14,15,16]. In addition, some AGPs possess other conservative domains such as extensins (EXTs) are also identified and termed as AGP/EXT hybrids (HAEs) [6].

Much of what we knew with respect to the identification and localization of AGPs traditionally resulted from the use of *β*-glucosyl Yariv reagents (*β*-Yariv; specifically bind to AGPs) or a set of AGP-specific monoclonal antibodies (mABs; recognize AGP carbohydrate epitopes) [17,18,19,20]. Still, AGPs cannot be specifically distinguished using these two reagents. With the increasing amount of whole genome sequencing data and transcriptome data originated from plants, it has been extremely crucial to identify and classify AGPs at the genomic level and transcriptome level. Using bioinformatic approaches, AGPs have been identified in a variety of higher plants, as well as mosses (*Sphagnum* sp., *Physcomitrella patens* and *Polytrichastrum formosum*), ferns (*Mohria caffrorum*) and even brown algae (*Ectocarpus siliculosus*) [3,4,5,6,8,9,10,11,21,22,23,24,25,26,27].

The affinity of AGPs to *β*-Yariv reagents and mABs is especially informative when backed up by independent functional analyses on specific AGPs using molecular genetics approaches, such as T-DNA-tagged lines, gene overexpression, RNA interference (RNAi) and antisense RNA, as well as transcriptomic techniques [5,28,29,30,31,32,33,34,35,36,37,38]. Indeed, such experimental approaches have demonstrated that AGPs are implicated in various processes of plant growth and development or adaption [39,40,41,42,43,44,45,46,47,48]. In addition, some studies have revealed that AGPs may be associated with signal transduction and response to multiple plant hormones [33,49,50]. Therefore, the identification and systematic analyses of the plant AGP family will facilitate our understanding of their biological functions.

Chinese cabbage (*Brassica rapa* ssp. *chinensis* Makino) is the most important economic leaf vegetable in Asia and an important resource for studying the evolution of polyploidy genomes [51,52]. In our previous study, a genic male-sterile system ‘*Bcajh97-01A/B*’ was developed in *B. rapa* [53]. Homozygous male-sterile line ‘*Bcajh97-01A*’ and heterozygous male-fertile line ‘*Bcajh97-01B*’ are sibling lines segregated in a 1:1 ratio. Mutation in *Male Meiosis Cytokinesis* interferes with the meiotic cytokinesis and tapetal programmed cell death, leading to microspore degeneration, pollen wall defects and thus complete male sterility [54]. Previously, a Perl script program was designed to enable the search for 33 classical AGPs, 28 AG-peptides and three Lys-rich AGPs in *B. rapa* [55]. In addition, 33 FLAs and 52 ENODLs were identified in the annotated genome of *B. rapa* using several prediction algorithms [51,56]. A Python script named “Finding-AGP” was creatively compiled to not only identify AGPs with high PAST percentage (>50%) but also cover most CAGPs with a relatively low PAST proportion, which eventually successfully discovered 264 AGPs in *B. rapa*, including five HAEs [10]. Nevertheless, the information with regard to the AGPs in *B. rapa* does not remain intact, with non-uniform standards.

In this study, in order to explore the function of AGPs in *B. rapa*, especially their roles in male reproductive development, first, we systematically identified and analyzed AGPs in *B. rapa*. We identified 293 putative *BrAGP* genes in the genome of *B. rapa*, including 25 classical AGPs, three Lys-rich AGPs, 30 AG-peptides, 36 FLAs, 59 PLAs, 33 XYLP genes, 102 other CAGP genes, two non-classical AGPs and three HAEs, and conducted a phylogenetic analysis. In addition, we found that 73 AGP-encoding genes in *B. rapa* are differentially expressed in floral buds between the sterile and fertile plants at least at one developmental stage. We further demonstrated that three FLA members, *BrFLA2*, *BrFLA28* and *BrFLA32*, are required for maintaining the proper timing of pollen germination under high relative humidity. Our study provides a resource for studying AGPs in *B. rapa* and reveals a role for AGPs in male reproductive development.

## 2. Results

### 2.1. Comprehensive Analysis of Putative AGP Genes in B. rapa

**Figure 1:**
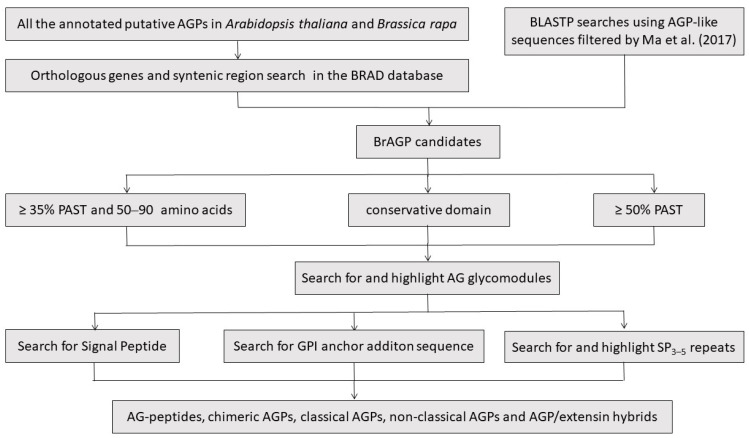
Schematic workflow of the integrated strategy in identification, classification and analysis of AGPs in *Brassica rapa*. Orthologous genes and syntenic searches of all the annotated putative AGPs in *Arabidopsis thaliana* and *B. rapa* were performed in the Brassica database (BRAD) to filter out AGP candidates. Additional BLASTP searches using AGP-like sequences were conducted in BRAD. Classical AGPs were defined as having greater than 50% PAST coupled with the presence of AG glycomodules and an N-terminal signal sequence. AG-peptides were defined to be between 50 and 90 amino acids in length with biased amino acid compositions of at least 35% PAST coupled with the presence of AG glycomodules and an N-terminal signal sequence. The presence of a GPI anchor addition sequence was used to provide added support for the identification of classical AGPs and AG-peptides. Chimeric AGPs were defined as possessing conservative domains coupled with the localized distribution of putative AG glycomodules. AGP/extensin hybrids were defined as containing two or more SP_3–5_ repeats and a characteristic used to identify AGPs. The remaining AGP candidates with biased amino acid compositions of at least 50% PAST were deemed as non-classical AGPs.

All the annotated putative BrAGPs in *B. rapa* were gathered and summarized based on previous studies, including 33 classical AGPs, 28 AG-peptides, three Lys-rich classical AGPs, 33 FLAs and 48 PLAs (28 BrENODLs, 11 BrUCLs, 8 BrSCLs and 1 unknown PLA) (Table S1) [51,55,56]. Those CAGPs that were not defined as an AGP for lacking N-terminal signal peptide came back in our consideration. The orthologous genes of all the annotated AtAGPs were searched in the Brassica database (BRAD, http://brassicadb.cn, V3.0, accessed on 1 December 2021). In this study, the total number of AtAGPs was 151 and included 23 classical AGPs, three Lys-rich classical AGPs, 16 AG-peptides, 22 FLAs, 28 PLAs, 22 XYLPs, 32 other CAGPs, four HAEs and one non-classical AGP (Table S2) [3,6,10,16,57]. In addition, BLASTP was performed in the BRAD using AGP-like sequences (part of whole protein sequences) filtered in Arabidopsis and *B. rapa*, respectively [10]. An overview of our integrated strategy to identify whole AGP families is shown in Figure 1.

Initially, 356 *B. rapa* AGP candidates were filtered out, among which 32 candidates were obtained by searching for proteins between 50 and 90 amino acids in length with biased amino acid compositions of at least 35% PAST (Table S3). Bra030409 (BrAGP42) was excluded here due to a PAST percentage below threshold and the absence of AG glycomodules. The presence of an N-terminal signal sequence provided additional supports for all AG-peptides except Bra040832 and Bra008021. In total, 30 potential AG-peptides were defined, the majority of which (28 of 30) were found to be GPI-anchored proteins. Two newly identified AG-peptides, Bra008765 and Bra023457, were designated as BrAGP46 and BrAGP47, respectively.

The remaining candidates longer than 90 amino acids in length were subjected to confirm the existence of conversed domains for the identification of CAGPs. Searching of the HMMERScan, Pfam (PF02469), SMART (SM00554) and InterPro (IPR000782) identified 39 AGP candidates predicted to possess fasciclin (FAS) domains (Table S3). The proteins with FAS domains were next manually scanned for the presence of putative AG glycomodules. It is noteworthy that three proteins (Bra009905, Bra034511 and Bra036584) were excluded from the FLA subfamily due to the lack of AG glycomodules. Ultimately, 36 *BrFLA* genes were identified in *B. rapa*. Three newly identified *BrFLA* genes, Bra018207, Bra007193 and Bra023589, were named as *BrFLA34*, *BrFLA35* and *BrFLA36*, respectively. A further algorithm predicted that two (BrFLA34 and BrFLA35) of these lack N-terminal secretion signal sequences and 23 of them (63.89%) are predicted to be GPI-anchored to the plasma membrane. In addition, 35 BrFLAs were predicted to have putative N-glycosylation sites.

Using the HMMERScan, Pfam (PF02298) and InterPro (IPR003245), a total of 65 plastocyanin-like (PCNL) domain-containing proteins were identified in *B. rapa* initially (Table S3). To check whether those identified proteins possess AG glycomodules, we manually analyzed Pro-containing sequence motifs in each of them, and discovered that all but six (Bra024208, Bra023459/BrENODL30, Bra009934/BrENODL16, Bra008763/BrENODL14, Bra021481 and Bra019625) contain these glycomodules. These 59 proteins with putative AG glycomodules were identified as BrPLAs, of which 53 were predicted to contain N-terminal signal sequences required for conventional protein secretion, but six (BrENODL7, BrENODL40, BrENODL48, BrENODL52, BrUCL4 and BrSCL2) did not. In addition, 54 BrPLAs were expected to have GPI anchor signal sequences and 39 had putative N-glycosylation sites. Based on motif constitution, all BrPLAs described here could be divided into four subfamilies: 35 ENODLs (including a newly identified *BrPLA* gene, Bra025873, named as *BrENODL53*), nine SCLs, 13 UCLs and two unknown PCNL-containing proteins (Bra019044 and Bra018282).

To identify XYLPs in *B. rapa*, we performed orthologous gene search of all the annotated AtXYLPs and additional BLASTP searches in the BRAD. After confirming the presence of non-specific lipid transfer protein 2 (nsLTP2) domains by submission to the HMMERScan, Pfam (PF14368), SMART (SM00499) and InterPro (IPR016140), 41 proteins were yielded (Table S3). According to the presence of a well-conserved eight-cysteine motif [7,9], Bra030067 and Bra039574 were excluded from the list of XYLP subfamily but classified as other CAGPs, and named as BrCAGP1 and BrCAGP2. It is noteworthy that the residue is conserved as a hydrophobic residue, always leucine, isoleucine or valine between Cys5 (C5) and Cys6 (C6) (Table S4). We then manually scanned all these BrXYLP protein sequences to ensure the presence of AG glycomodules. Putative AG glycomodules in all BrXYLPs except for six (Bra030873, Bra012033, Bra015985, Bra015984, Bra008102 and Bra027016) were found to be distributed before and/or after the nsLTP2 domain (Table S4), suggesting that 33 BrXYLPs may be chimeric AGPs. We named them as BrXYLP1–BrXYLP33.

Analogously to already classified chimeric subfamilies, such as FLA, PLA and XYLP, extra types of CAGPs were explored. A total of 100 candidates with other conservative domains and indispensable AG glycomodules were obtained by the integrated screening method of AGP prediction (Table S3). Typically, there were 25 CAGPs with protein kinase (PKinase)-like domains, 11 CAGPs with glycosyl hydrolase (GH)-like domains, nine CAGPs with formin homology 2 (FH2)-like domains, seven CAGPs with glycerophosphoryl diester phosphodiesterase (GDPD)-like domains, seven CAGPs with pollen Ole e I (POeI)-like domains, four CAGPs with leucine-rich repeat (LRR)-like domains, five CAGPs with X8-like domains and five CAGPs with pectin methylesterase inhibitor (PMEI)-like domains. It is noteworthy that BrAGP55, documented as a classical AGP in the literature [55], was classified into the list of CAGP subfamily here as it possessed a Nsp1-like C-terminal region. Among these newly identified CAGPs, seven members (Bra013339, Bra002969, Bra017734, Bra027650, Bra020846, Bra038210 and Bra022423) also have characteristics of EXTs with the presence of the SP_3_ tetrapeptide and SP_4_ pentapeptide motifs. We termed them as chimeric AGP–EXT hybrids (BrCHAE1–BrCHAE7). The remaining CAGPs are named as BrCAGP3–BrCAGP94. The majority (83 of 100) were predicted to have a signal peptide, whereas only some (34 of 100) were predicted to have a GPI anchor.

Besides the seven BrCHAEs identified above, additionally three HAEs (Bra030020, Bra009880 and Bra014023) were yield with a biased amino acid composition of PAST greater than 50% and sequence characteristics of both AGPs and EXTs, and termed as BrHAE1, BrHAE2 and BrHAE3 (Table S3).

After removing proteins belonging to AG-peptides, CAGPs and HAEs, the remining sequences were retained for further analysis. Initially, 25 classical AGP candidates were searched for biased amino acid compositions of at least 50% PAST and the presence of N-terminal signal sequences and putative AG glycomodules, among which three (BrAGP17, BrAGP18.1 and BrAGP18.2) are Lys-rich AGPs (Table S3). The presence of a GPI-modified anchor sequence provided additional supports for all classical AGPs except Bra011948 (BrAGP54.1). Four candidate proteins, Bra027649 (BrAGP52), Bra027648 (BrAGP53), Bra035700 (BrAGP54.2) and Bra038294 (BrAGP57), previously identified as AGPs by [55], were excluded from the list of classical AGPs here due to the biased amino acid composition below the PAST threshold. In addition, three additional classical AGPs, Bra032796 (BrAGP50.1), Bra010966 (BrAGP50.2) and Bra012505 (BrAGP50.3), are below the 50% PAST threshold but were identified by searching the Brassica protein database annotations and the presence of putative AG glycomodules, N-terminal signal sequences and GPI-modified anchor sequences. These three AGPs were added to the list of classical AGPs. Then, PAST threshold, signal peptide and AG glycomodule filtration resulted in two non-classical AGPs, Bra040103 (BrAGP58.1) and Bra021074 (BrAGP58.2).

In conclusion, a total of 293 putative *BrAGP* genes were screened out here, including 25 classical AGPs, three Lys-rich AGPs, 30 AG-peptides, 36 FLAs, 59 PLAs, 33 XYLP, 102 other CAGPs, two non-classical AGPs and three HAEs (Table S3). The classical AGPs ranged in size from 115 to 339 amino acids; the AG-peptides ranged in size from 58 to 90 amino acids; the FLAs ranged in size from 201 to 457 amino acids; the PLAs ranged in size from 118 to 713 amino acids; the XYLPs ranged in size from 146 to 387 amino acids; other CAGPs ranged in size from 141 to 1071 amino acids. Compared with the results in previous studies [51,55,56], 153 additional putative BrAGPs were screened out. These newly identified BrAGPs included two AG-peptides, three FLAs, 11 PLAs, 33 XYLP genes, 101 other CAGP genes and three HAEs (Table S3). The protein backbones of newly identified BrAGPs, including the predicted locations of their signal peptides, GPI anchor addition sequences and conversed domains, are displayed in Table S4.

### 2.2. Phylogenetic Analysis of BrAGPs and AtAGPs

**Figure 2:**
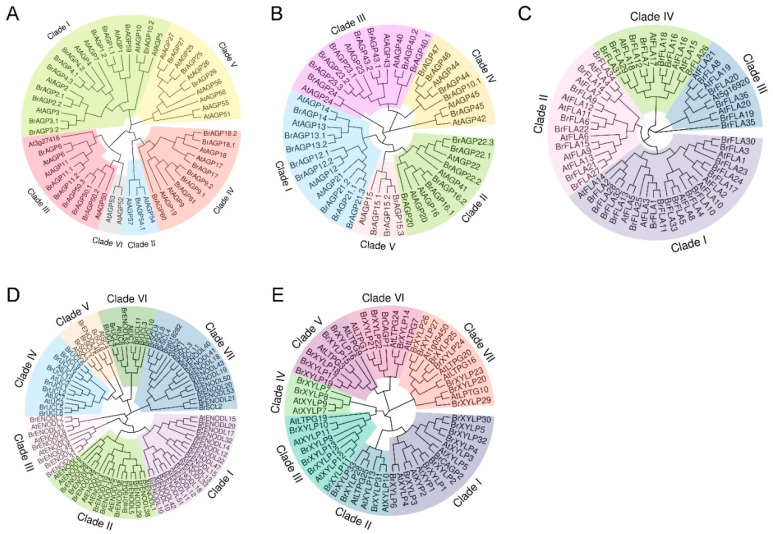
Phylogenetic tree representation of putative AGPs from *Brassica rapa* (BrAGPs) and Arabidopsis (AtAGPs). (**A**) Classical AGPs and Lys-rich AGPs. (**B**) AG-peptides. (**C**) Fasciclin-like AGPs (FLAs). (**D**) Phytocyanin-like AGPs (PLAs). (**E**) Xylogen-like proteins (XYLPs). The neighbor-joining tree was constructed using MEGA X software with 1000 bootstraps. The OmicStudio tool was used to display and manipulate trees.

Comparative sequence analysis of *AGP* genes in Arabidopsis and *B. rapa* was performed to explore their evolution and divergence. In total, 151 AGPs in Arabidopsis and 293 in *B. rapa* were aligned (Tables S2 and S3) [3,6,10,16,57]. An unrooted neighbor-joining phylogenetic tree was generated from the alignments of full-length protein sequences among BrAGPs and AtAGPs (Figure 2). Based on their sequence homology, all of the AGPs were clustered into distinct clades according to different subfamilies.

A previous study clustered 55 classical AGPs from *B. rapa* and Arabidopsis into six clades and 44 AG-peptides into three clades [55]. Similarly, our results showed that 25 classical BrAGPs, three Lys-rich BrAGPs, 23 classical AtAGPs and three Lys-rich AtAGPs can also be classified into six clades with high bootstrap support (Figure 2A). All members belonging to Clade I contained motif 1, motif 2 and motif 3, except for AtAGP1 and AtAGP10, which only had motif 1 and motif 2. BrAGP54.1, AtAGP54 and AtAGP57 in Clade II possessed motif 1 and motif 3, while AtAGP52 and AtAGP53 in Clade VI only had motif 2. Clade III could be further mainly classified into three types: a type with motif 1, motif 2 and motif 5 (BrAGP6, BrAGP11.1, BrAGP11.2, AtAGP6 and AtAGP11), another type with motif 1, motif 2 and motif 4 (BrAGP50.1, BrAGP50.2, BrAGP50.3 and AtAGP50) and At3g27416 that only included motif 1. All Lys-rich AGPs are divided into Clade IV, most members of which contained motif 1, motif 2 and motif 3. Nine of ten AGPs in Clade V had motif 1. All predicted locations of motifs are presented in Figure S1.

Phylogenetic analysis of 30 AG-peptides from *B. rapa* together with 16 AG-peptides from Arabidopsis led to five clades, among which Clade IV and Clade V were new (Figure 2B). Two members (BrAGP24 and AtAGP24) in Clade III, five members (BrAGP44, BrAGP45, AtAGP42, AtAGP44 and AtAGP45) in Clade IV and all four members (BrAGP15.1, BrAGP15.2, BrAGP15.3 and AtAGP15) in Clade V were previously defined as orphan genes in [55]. Motif analysis showed that almost all AG-peptides possessed motif 6 and motif 8, and most of them additionally had motif 7 (Figure S2).

Protein-based phylogenetic analysis and pair-wise sequence comparison of 36 BrFLAs and 22 AtFLAs were conducted. Four clades could be distinguished (Figure 2C), which was consistent with previous studies in Arabidopsis and Chinese cabbage [4,56]. Newly identified BrFLA34 was placed in Clade II, whereas BrFLA35 and BrFLA36 both belonged to Clade III. Depending on the motif analysis, all BrFLAs and AtFLAs possessed motif 11, and all members in Clade IV additionally included four motifs (motif 12–motif 15) (Figure S3).

A previous study indicated that the 48 BrPCs, which were CAGPs with putative AG glycomodules, can be divided into seven groups [51]. Here, we found that 59 BrPLAs and 28 AtPLAs can also be classified into seven clades, named Clade I–Clade VII (Figure 2D). There were 12, 10, 6, 8, 4, 6 and 13 BrPLAs in each clade, respectively (Figure 2D). All BrPLAs in Clade I, Clade II and Clade III belonged to the ENODL subfamily except an unknown PCNL-containing protein Bra019044 (Figure 2D). Almost all of them (41 in 43) had motif 16–motif 19, the majority of which also contained motif 20 (Figure S4). All BrUCLs were clustered into Clade IV and Clade VI, where Clade IV had only UCL members and Clade VI contained an additional ENODL (Figure 2D). Motif constitution analysis showed the presence of motif 16–motif 18 in most of the UCLs in Clade IV (Figure S4). All members in Clade VI possessed motif 16–motif 19 except for BrENODL52, which only had motif 19 (Figure S4). There were three BrSCLs with motif 16–motif 19 and one BrENODL in Clade V (Figure 2D and Figure S4). Clade VII consisted of 18 members, including 12 BrENODLs and one unknown PCNL-containing protein Bra018282. Except for BrSCL2, all members in Clade VII contained motif 19 (Figure S4).

Previous studies indicated that the 11 AtXYLPs belonging to the AGP family can be divided into four clades [7,9]. Here, manual analysis of the phylogenetic tree revealed seven distinct clades of 33 BrXYLPs, two nsLTP2-containing BrCAGPs and 22 AtXYLPs (Figure 2E). Clade I was composed of 14 members, each of which had motif 21, motif 22 and motif 23. Clade II consisted of six members with motif 22 and motif 23, and three members (BrXYLP31, AtXYLP10 and AtXYLP13) also had motif 21. All eight members in Clade III and all eleven members in Clade VII had motif 21 and motif 22. All BrXYLPs in Clade V and Clade VI contained motif 21, motif 22 and motif 24 except BrCAGP1 that lacked motif 22. All predicted locations of motifs are shown in Figure S5.

Phylogenetic analysis of the remaining BrCAGPs was also conducted and contributed to the study of evolutionary relationships among these proteins with other conservative domains, such as the PKinase-like domain, GH-like domain, FH2-like domain and GDPD-like domain (Figure S6A,B). In addition, a protein-based phylogenetic tree of seven BrCHAEs, three BrHAEs and four AtHAEs was generated to aid the relationship investigation of putative AGPs with characteristics of EXTs (Figure S6C).

### 2.3. Chromosomal Distribution of BrAGP Genes and Gene Duplication

**Figure 3:**
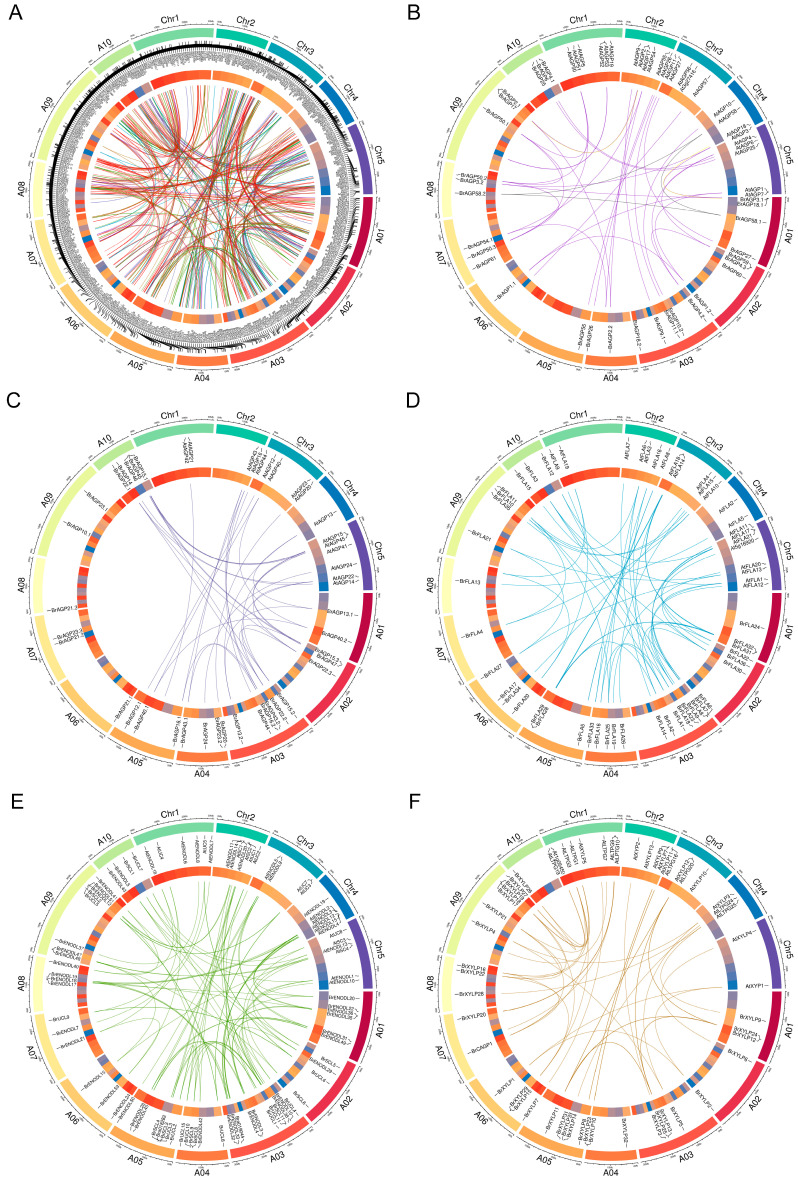
Circos plots showing *AGP* genes on chromosomes of the Arabidopsis genome and the *Brassica rapa* genome. (**A**) All AGPs. (**B**) Classical AGPs and Lys-rich AGPs. (**C**) AG-peptides. (**D**) Fasciclin-like AGPs (FLAs). (**E**) Phytocyanin-like AGPs (PLAs). (**F**) Xylogen-like proteins (XYLPs). From the outside in, the first layer of the Circos plot is a chromosome map of the Arabidopsis genome and the *B. rapa* genome. The 24 conserved collinear blocks on the ten chromosomes of the *B. rapa* genome and the five chromosomes of the Arabidopsis genome were color coded in the second circle. The network in the center of the plot represents the syntenic relationships between *BrAGPs* and *AtAGPs* according to the syntenic gene search in the Brassica database. Classical AGPs were linked in dark violet lines, Lys-rich AGPs in yellow lines, non-classical AGPs in gray lines, AG-peptides in dark slate blue lines, FLAs in dodger blue lines, PLAs in green lines, XYLPs in sandy brown lines, other chimeric AGPs (CAGPs) in red lines and AGP/extensin hybrids (HAEs) in navy blue lines.

According to the information of the approximate position obtained from the BRAD, each *BrAGP* gene was marked on the physical map of *B. rapa* chromosomes. A total of 286 *BrAGP* genes were randomly distributed among the 10 chromosomes of *B. rapa*, except for seven *BrAGP* genes (*BrAGP9.2*, *BrAGP11.2*, *BrAGP12.2*, *BrUCL16*, *BrENODL52*, *BrXYLP30* and *BrCAGP2*), for which only scaffold information was available (Figure 3). In total, 38 genes were located on chromosome A01, 20 genes on chromosome A02, 53 genes on chromosome A03, 26 genes on chromosome A04, 27 genes on chromosome A05, 21 genes each on chromosomes A06 and A08, 18 genes on chromosome A07, 40 genes on chromosome A09 and 22 genes on chromosome A10 (Table S3 and Figure 3).

The *BrAGP* genes were distributed across the three sub-genomes, with 120 *BrAGP* genes in LF (the least fractionated blocks of *B. rapa*), 92 in MF1 (the medium fractionated blocks of *B. rapa*) and 78 in MF2 (the most fractionated blocks of *B. rapa*), while three members, *BrENODL52*, *BrXYLP30* and *BrCAGP2*, had no hits in BRAD (Table S5). Among these *BrAGPs*, 42 genes had no syntenic orthologs in the Arabidopsis genome (Table S5), which could have been originated by gene transposition in *B. rapa* after its divergence from Arabidopsis [58]. In total, there were 244 *BrAGPs* syntenic to 156 genes in Arabidopsis (Table S5), suggesting that most *BrAGP* genes were retained in *B. rapa* after whole-genome triplication and fractionation. Furthermore, the retained homologous *BrAGP* gene copies in the three *B. rapa* sub-genomes had the same conserved collinear blocks (Figure 3 and Tables S3 and S5). The occurrence of genome triplication in *B. rapa* was clearly observed and well supported, as 24 *AtAGP* genes in the Arabidopsis genome had three syntenic copies in *B. rapa* after removing the redundancy of duplicated tandem genes (keeping one gene from each tandem array), such as *AtAGP50*, *AtAGP15*, *AtFLA14*, *AtENOLD1* and *AtXYLP12* (Figure 3 and Table S5). A total of 138 Arabidopsis genes, including 131 *AtAGPs*, had one or two syntenic orthologs in *B. rapa*. Additionally, there were 24 *AtAGPs* with no syntenic counterparts in *B. rapa*, such as *AtAGP7*, *AtAGP19*, *AtFLA2*, *AtENODL4* and *AtHAE3* (Table S5).

In the light of the large size of the *BrAGP* gene family in *B. rapa*, the importance of segmental duplication of chromosomal regions and tandem duplication generating nearby gene copies to the size and evolution of the *BrAGP* gene family was investigated. We found that 194 (66.21%) of the 293 *BrAGP* genes resulted from duplications, of which 183 were located in the duplicated chromosomal segments of the *B. rapa* chromosomes, such as *BrAGP1.1* and *BrAGP1.2*; *BrFLA2*, *BrFLA28* and *BrFLA32*; *BrXYLP4* and *BrXYLP5* (Table S5 and Figure 3). These results showed that segmental gene duplication was the preferential reason for the expansion of this gene family. Additionally, 15 *BrAGP* genes were found to be tandemly duplicated: eight tandemly duplicated genes were distributed on chromosome A09, two genes each on A04 and A05 and five genes on A03 (Tables S3 and S5). In all cases of tandem duplications, the amino acid similarities were ≥70%, except for two pairs (*BrSCL3* and *BrSCL4*, *BrCAGP27* and *BrCAGP28*) that only shared 37.11% and 44.38% amino acid similarities. Interestingly, four groups of *BrAGP* genes (group I: *BrXYLP15*, *BrXYLP16*, *BrXYLP17*, *BrXYLP18* and *BrXYLP19*; group II: *BrCAGP6*, *BrCAGP7* and *BrCAGP8*; group III: *BrCAGP27*, *BrCAGP28* and *BrCAGP29*; group IV: *BrCAGP81*, *BrCAGP82*, *BrCAGP83*, *BrCAGP84* and *BrCAGP85*) were expanded through both segmental and tandem duplications (Table S5).

### 2.4. BrAGP Genes Are Developmentally Regulated and Male Fertility-Related

In view of our previous focus on pollen development and function and pollen tube growth, here we were more concerned with AGPs that may be candidates for these unique processes. To investigate whether the identified AGP-encoding genes are male fertility-related, we estimated the expression levels of each *BrAGP* gene in floral buds at five developmental stages by calculating fragments per kilobase of transcript per million mapped reads (FPKMs) based on the previous RNA sequencing datasets. We found that 73 *BrAGP* genes had at least differential expression in at least one stage of the fertile floral buds compared with the sterile ones (Table 1). These AGPs include nine classical AGPs, one Lys-rich AGP, eight AG-peptides, ten FLAs, 12 PLAs, 12 XYLPs, 17 CAGPs, two HAEs and two non-classical AGPs. These data show the dynamic changes of AGP-encoding gene expression in response to floral bud development and suggest their roles in pollen ontogenesis.

### 2.5. Expression of Three BrFLA Genes Is Tissue-Specific and Developmentally Controlled

**Figure 4:**
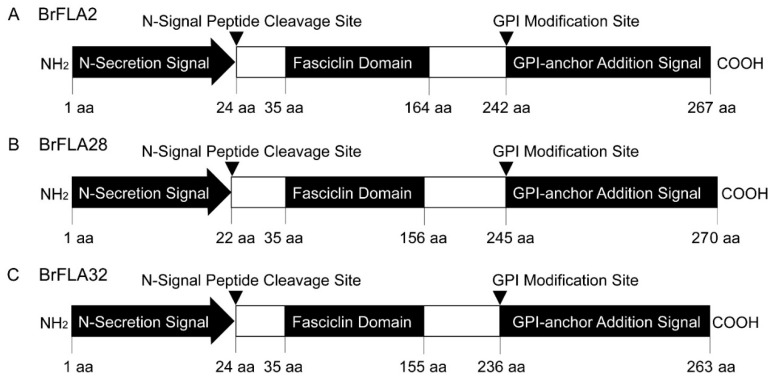
Structure schematic representation of three BrFLA proteins deduced from DNA sequences. (**A**) BrFLA2. (**B**) BrFLA28. (**C**) BrFLA32.

The finding that some AGP-encoding genes are dysregulated in the sterile floral buds relative to the fertile ones of some specific periods prompted us to study the biological importance of such a family. We focused on three FLA-encoding genes, *BrFLA2* (Bra001464), *BrFLA28* (Bra034746) and *BrFLA32* (Bra038741). The full-length DNA and cDNA sequences were obtained by PCR amplification (Figure S7A–F). The result of the corresponding cDNA and DNA product sequencing confirmed that *BrFLA2*, *BrFLA28* and *BrFLA32* are 804 bp, 813 bp and 792 bp in length, respectively, with no introns in their sequences. Their protein sequences deduced from the DNA sequence are 267, 270 and 263 amino acids in length, with an amino acid similarity ≥73.04% to each other and high homology (≥82.07%) with Arabidopsis *AtFLA14* (Figure S7G). *BrFLA2*, *BrFLA28* and *BrFLA32* are expected to have an N-terminal signal peptide sequence and a C-terminal GPI anchor signal sequence (Figure 4). The PAST content of their core protein backbone is about 40%. They all possess a FAS domain with lengths of 130 aa, 122 aa and 121 aa, which contain H1 and H2 conserved regions (Figure S7G and Figure 4).

**Figure 5:**
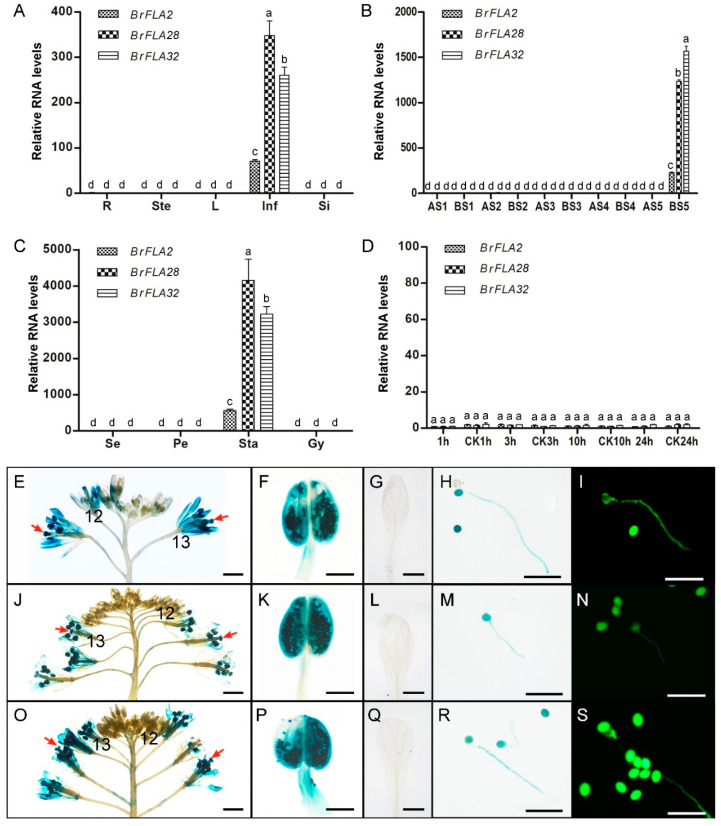
Expression analysis of *BrFLA2*, *BrFLA28* and *BrFLA32* in genic male-sterile system ‘*Bcajh97-01A/B*’ of *Brassica rapa*. (**A**–**D**) Quantitative real-time PCR analysis of *BrFLA2*, *BrFLA28* and *BrFLA32*. The values are the mean ± standard deviation (SD). Different letters indicate statistical significance (*p* < 0.01) as determined by a one-way ANOVA test. R, root; Ste, stem; L, young leaf; Inf, inflorescence; Si, germinal silique; A, homozygous male-sterile line ‘*Bcajh97-01A*’; B, heterozygous male-fertile line ‘*Bcajh97-01B*’; S1–S5, pollen mother cell stage, tetrad stage, uninucleate stage, binucleate stage and mature pollen stage; Se, sepal; Pe, petal; sta, stamen; Gy, gynoecium; 1, 3 and 10 h, the corresponding time after pollination; CK, the corresponding un-pollinated pistils. (**E**–**H**, **J**–**M**, **O**–**R**) GUS staining in *P*_*BrFLA*2_::GUS (**E**–**H**), *P*_*BrFLA*28_::GUS (**J**–**M**) and *P*_*BrFLA*32_::GUS (**O**–**R**) transgenic plants. (**E**,**J**,**O**) Stained inflorescences. (**F**,**K**,**P**) Magnified images of an anther as shown in (**E**,**J**,**O**). (**G**,**L**,**Q**) Stained petals. (**H**,**M**,**R**) Stained pollen grains and pollen tubes germinated in vitro. (**I**,**N**,**S**) GFP expression in *P*_*BrFLA*2_::GUS (**I**), *P*_*BrFLA*28_::GUS (**N**) and *P*_*BrFLA*32_::GUS (**S**) transgenic pollen grains and pollen tubes. Scale bars = 1 mm (**E**,**J**,**O**); 250 μm (**F**,**K**,**P**); 500 μm (**G**,**L**,**Q**); 50 μm (**H**,**I**,**M**,**N**,**R**,**S**).

According to the above analysis, these three *BrFLA* genes were specially expressed in floral buds at late microspore developmental stages and down-regulated in the floral buds of sterile plants (Table 1), suggesting their potential involvement in reproductive development. We validated the expression pattern of these three *BrFLA* genes in floral buds at different developmental stages using quantitative real-time PCR. We found that *BrFLA2*, *BrFLA28* and *BrFLA32* showed similar patterns of expression, and they were only abundant in floral buds at the mature pollen stage, particularly in stamens (Figure 5B,C). The determination of the expression pattern in various vegetative tissues such as roots, leaves, stems and other reproductive tissues such as germinal siliques, pollinated and un-pollinated pistils 1, 3 and 10 h after pollination (HAP) further revealed that the expression of *BrFLA2*, *BrFLA28* and *BrFLA32* was stamen-specific (Figure 5A–D). Overall, *BrFLA2* expression levels were lower than those of *BrFLA28* and *BrFLA32*.

Additionally, to investigate the expression of these three *BrFLA* genes at the tissue level, the expression of the GUS gene under the control of the *BrFLA2*, *BrFLA28* and *BrFLA32* promoter, respectively, was examined in transgenic Arabidopsis plants. A concordant expression was assessed and only floral buds at the anthesis stage displayed GUS activity (Figure 5E,J,O). The expanded GUS signal in the petals was determined as a result of GUS diffusion, as no GUS activity was observed when the petals were stained alone (Figure 5G,L,Q). GUS staining was restricted to the anthers (Figure 5F,K,P), stigma and transmitting tract (Figure 5E,J,O). Pollen tubes germinated in vitro also demonstrated GUS staining (Figure 5H,M,R), which was consistent with the result of GFP fluorescence signal observation (Figure 5I,N,S), indicating that the GUS staining in the pistils of floral buds at the anthesis stage might be from pollen tubes rather than female organs. These results revealed that similar expression patterns of *BrFLA2*, *BrFLA28* and *BrFLA32* are developmentally controlled, with floral buds being solely stained, and tissue-specific, with anthers, pollen grains and pollen tubes displaying GUS staining.

### 2.6. BrFLA2, BrFLA28 and BrFLA32 Proteins Are Localized at the Plasma Membrane and in the Hechtian Strands

**Figure 6:**
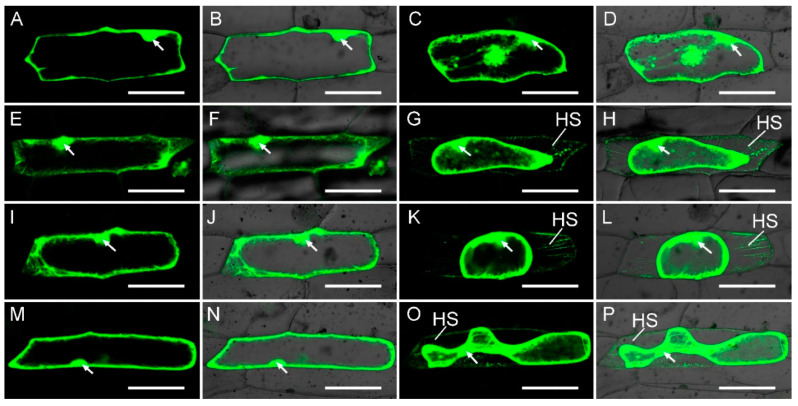
Expression of GFP–BrFLA fusion constructs in transgenic onion epidermal cells. Detection of GFP fluorescence in onion epidermal cells expressing pFGC–eGFP (Control) (**A**–**D**), GFP–BrFLA2 (**E**–**H**), GFP–BrFLA28 (**I**–**L**) and GFP–BrFLA32 (**M**–**P**) before (**A**,**B**,**E**,**F**,**I**,**J**,**M**,**N**) and after (**C**,**D**,**G**,**H**,**K**,**L**,**O**,**P**) treatment with 0.3 g·mL^−1^ sucrose for 3 min. White arrows indicate the nuclei of onion epidermal cells. HS, Hechtian strands. Scale bars = 100 μm.

To assess the subcellular distribution of BrFLA2, BrFLA28 and BrFLA32 proteins, these three *BrFLA* genes were truncated to form eGFP-fused constructs under the control of the constitutive CaMV 35S promoter. Onion epidermal cells harboring eGFP–BrFLA2, eGFP–BrFLA28 and eGFP–BrFLA32 showed an intense peripheral fluorescence (Figure 6E,F,I,J,M,N). In plasmolyzed eGFP–BrFLA2 cells, the eGFP fluorescence occurred only at the surface of the protoplast rather than in the cell wall (Figure 6G,H). Intriguingly, the eGFP–BrFLA2 signal was also obvious in the Hechtian strands (plasma membrane extensions connecting the cell wall and the plasma membrane) after the membrane was retracted during plasmolysis in 0.3 g·mL^–1^ sucrose. The cases in onion epidermal cells expressing eGFP–BrFLA28 and eGFP–BrFLA32 displayed a similar and clearly visible localization of eGFP at the plasma membrane and in Hechtian strands that remained attached to the cell wall (Figure 6I–P), while the pFGC–eGFP (Control) was exclusively membrane-localized (Figure 6A–D).

### 2.7. Suppression of BrFLA2, BrFLA28 and BrFLA32 Genes’ Expression Promotes Pollen Grain Germination under High Humidity

**Figure 7:**
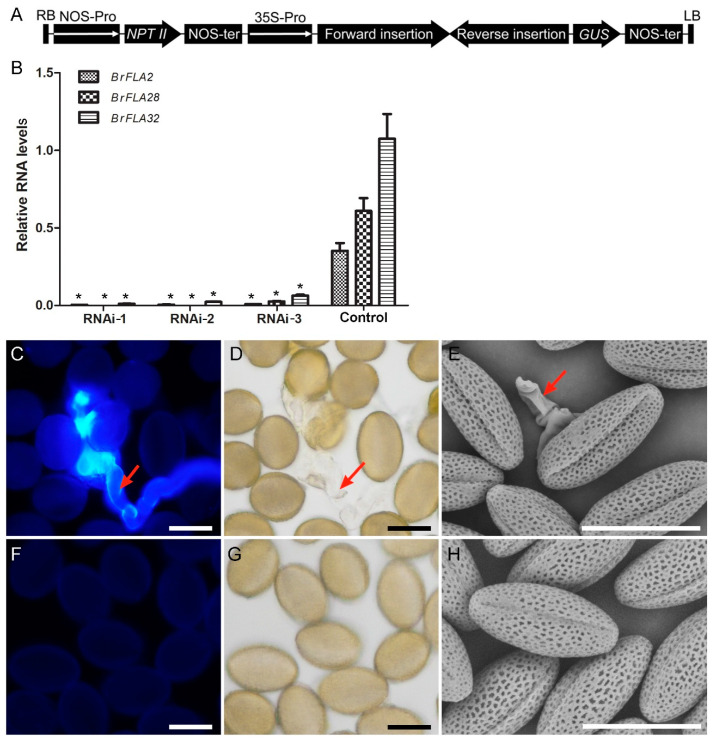
Effects of 85% relative humidity on dehiscent pollen of *BrFLA2/28/32*–RNAi transgenic plants in *Brassica rapa*. (**A**) Schematic representation of the T-DNA portion of RNAi recombinant plasmid. (**B**) Expression of *BrFLA2*, *BrFLA28* and *BrFLA32* in RNAi plants by quantitative real-time PCR. RNA was extracted from the inflorescence. The values are the mean ± standard deviation (SD). * indicates significantly different means (*p* < 0.05) using one-way ANOVA. (**C**–**H**) Effects of 85% relative humidity on dehiscent pollen after 3d. (**C**,**D**) Pollen from a *BrFLA2/28/32*–RNAi plant stained with aniline blue. (**D**) The corresponding bright field image. (**E**) Pollen from a *BrFLA2/28/32*–RNAi plant observed under scanning electron microscopy. (**F**,**G**) Pollen from a control plant stained with aniline blue. (**G**) The corresponding bright field image. (**H**) Pollen from a control plant observed under scanning electron microscopy. Scale bars = 30 μm (**C**–**H**).

Since *BrFLA2*, *BrFLA28* and *BrFLA32* share a high homology and a similar expression pattern with each other, to study the exact biological function of these three *BrFLA* genes, we generated RNA interference (RNAi) transgenic plants (Figure 7A). We selected three lines (1, 2 and 3) that revealed marked down-regulation of *BrFLA2*, *BrFLA28* and *BrFLA32* expression in the transgenic plants (Figure 7B and Figure S8) for further investigation. The *BrFLA2/28/32*–RNAi transgenic plants and control plants showed no differences in vegetative tissues (data not shown). Additionally, RNAi transgenic plants did not show abnormal phenotypes in reproductive organs, such as stamens and gynoecium (Figure S9). In standard growth conditions under long-day light (16 h of light/8 h of dark) with 50% relative humidity, dehiscent pollen (Figure S10A–D,I–L) from RNAi plants had normal morphological appearance for microscopy, showed active pollen vitality and pollen germination frequencies in vitro and displayed normal fluorescence in the three nuclei detected by 4′,6-diamidino-1-phenylindole (DAPI) solution and normal cellulose distribution seen by calcofluor fluorescent white stain, compared with control pollen (Figure S10E–H,M–P).

A case in AGP40, a pollen-specific AG-peptide, in which the null mutant shows no alteration in pollen grain development but illustrates a reduction in pollen grain fitness [59], and an early pollen germination in *agp6 agp11* null mutants inside the anther [40] gave us a clue and encouraged anther experiments to further investigate this phenotype. We placed the *BrFLA2/28/32*–RNAi transgenic plants and control plants in chambers with an 85% relative humidity. After 3 d, dehiscent pollen was gathered and stained with aniline blue. Light microscopy revealed that approximately 9.5% of pollen grains germinated precociously in the anthers of *BrFLA2/28/32*–RNAi transgenic plants, with prominent callose fluorescence signals in the pollen tubes detected by aniline blue (Figure 7C,D), whereas dehiscent pollen from control plants had negligible callose staining within the grains (Figure 7F,G). Scanning electron microscopy further confirmed the result of aniline blue staining that early germinated pollen tubes from *BrFLA2/28/32*–RNAi transgenic plants were discernible (Figure 7E) and there was no pollen tube outgrowth in the anthers of control plants (Figure 7H). This dramatic phenotype suggests that high humidity can affect the *BrFLA2/28/32*–RNAi phenotype, enhancing early pollen germination in the anthers.

## 3. Discussion

As a first step in research on gene families and as a useful strategy in general, a genome-wide analysis of the AGP gene family has been performed in a variety of higher plants, such as Arabidopsis, tobacco (*Nicotiana tabacum*), rice (*Oryza sativa*), tomato (*Lycopersicon esculentum*), as well as mosses, ferns and even brown algae [3,4,5,6,8,9,10,11,21,22,23,24,25,26,27]. Although some special subfamilies of AGPs in *B. rapa* have been identified, such as classical AGPs, AG-peptides, FLAs and PLAs [10,51,55,56], no comprehensive study has been carried out on the whole of the *AGP* gene family in *B. rapa* and the information was fragmented. In this study, after the calculation of PAST content and the length of amino acid sequence length, confirming the presence of conserved domains and N-terminal signal peptides and manual prediction of the putative AG glycomodules, we identified 293 *AGP* genes in *B. rapa*, including 25 classical AGPs, three Lys-rich AGPs, 30 AG-peptides, 36 FLAs, 59 PLAs, 33 XYLPs, 102 other CAGPs, two non-classical AGPs and three HAEs (Figure 1 and Table S3). We classified *BrAGP* genes into several distinct clades according to different subfamilies based on their sequence homology (Figure 2 and Figures S1–S6).

Tandem and segmental duplications during evolution represent the major mechanism for gene family expansion [60]. *BrAGP* genes are randomly located on ten *B. rapa* chromosomes. The large size of members of the *BrAGP* gene family indicated that it was retained from frequent duplication events during evolution. Gene duplication analysis revealed that 183 (62.46%) of the 293 *BrAGP* genes were derived from segmental duplication and six clusters of tandemly duplicated genes (17 *BrAGP* genes) were present on chromosomes A03, A04, A05 and A09 (Table S5 and Figure 3). Our analyses indicated that chromosomal segment duplications contributed most to the expansion of this gene family.

Several studies have suggested that AGPs participate in a multitude of vital activities related to plant growth and development [18,61,62,63,64,65,66,67,68,69,70]. Expression analysis of all *BrAGP* genes based on our previous RNA sequencing data revealed that 73 (24.91%) of the 293 *BrAGP* genes were differentially expressed in floral buds between the sterile and fertile plants at least at one developmental stage, including nine classical AGPs, one Lys-rich AGP, eight AG-peptides, ten FLAs, 12 PLAs, 12 XYLPs, 17 CAGPs, two HAEs and two non-classical AGPs (Table 1). Thus, it is possible that these AGP members are involved in pollen ontogenesis.

The two sequential phases of angiosperm pollen ontogenesis, a developmental phase leading to mature pollen grain formation and a functional phase beginning with the activation of pollen grain by rehydration on the stigma surface and ending at double fertilization, have been the focus of substantial research because of their biological importance and uniqueness [71]. Among the previous studies on the molecules involved in pollen ontogenesis, several members in AGPs have been highlighted, especially during the crucial and complex process of male reproductive development and function [35,40,44,72]. For example, six AGPs in Arabidopsis, namely two classical AGPs (AGP6 and AGP11), two FLAs (FLA3 and FLA14) and two AG-peptides (AGP23 and AGP40), are specifically expressed in pollen and/or pollen tubes and found to be involved in microspore development as well as subsequent pollen germination [35,39,40,59,72,73]. This is also the case for several pollen-preferentially expressed AGPs derived from *B. rapa*, an AG-peptide (BAN102) and two classical AGPs (BcMF8 and BcMF18) [44,74]. In rice, several AGP-encoding genes are also dominantly expressed in anthers, among which *OsAGP7*, *OsAGP10* and *MTR1* are highly expressed in pollen and a mutation in *MTR1* results in defects in both tapetum and microspore development, eventually causing complete male sterility [5,75]. In our study, we characterized three anther, pollen grain and pollen tube-specific *BrFLA* genes, *BrFLA2*, *BrFLA28* and *BrFLA32*, and identified *BrFLA2/28/32*–RNAi transgenic plants that have an increased ratio of pollen grains germinated precociously in the anthers under high humidity compared with control plants (Figure 5 and Figure 7). These results suggested that *BrFLA2*, *BrFLA28* and *BrFLA32* are required during the late stage of pollen ontogenesis.

## 4. Materials and Methods

### 4.1. Plant Materials and Growth Conditions

Homozygous male-sterile plants ‘*Bcajh97-01A*’ and heterozygous male-fertile plants ‘*Bcajh97-01B*’ were cultivated in the experimental farm of Zhejiang University, Hangzhou, China. Arabidopsis, ecotype Columbia-0, was grown on the potting mix at 22 ± 2 °C under long-day conditions with a 16 h light and 8 h dark cycle.

### 4.2. Identification of BrAGP Genes and Bioinformatics Analysis

All the annotated putative AGPs in Arabidopsis and *B. rapa* referred to previous studies [3,6,16,51,55,56,57]. Orthologous genes and syntenic region search were performed in the BRAD. Additional BLASTP searches using AGP-like sequences (part of whole protein sequences) filtered by [10] were conducted. The characteristics of AGPs have been widely described, but the only controversy is whether a signal peptide sequence at the N-terminal should be optional. Some special FLA members in wheat (*Triticum aestivum*) and rice (*O. sativa*) lack signal peptides [76]. In addition, the identification of AGP32I, a CAGP, lacking signal peptides in Arabidopsis is also challenging our conventional concept to define an AGP [6]. Because of this reason, the candidate chimeric proteins that have been excluded from the AGP category for lacking an N-terminal signal peptide, such as some BrENODLs and BrFLAs [51,56], were reconsidered. However, the presence of an N-terminal secretion signal is constantly thought to be a prerequisite for classical AGPs and AG-peptides in this study.

PAST content, PVKCYT content and the length of the protein were calculated for each putative AGP. All AGP candidates were first subjected to HMMER (https://www.ebi.ac.uk/Tools/hmmer/, accessed on 1 December 2021) to confirm the existence of conserved domains. Proteins with FAS domains were then identified using Pfam (http://pfam.xfam.org/, PF02469, accessed on 1 December 2021), SMART (http://smart.embl-heidelberg.de/, SM00554, accessed on 1 December 2021) and InterPro (http://www.ebi.ac.uk/interpro/, IPR000782, accessed on 1 December 2021) databases [77,78,79]. The PCNL domain was extracted using prediction algorithms (Pfam, PF02298, and InterPro, IPR003245) [77,78]. Pfam (PF14368), SMART (SM00499) and InterPro (IPR016140) searches were performed to confirm the existence of nsLTP2 domains. In addition, the conserved eight-cysteine motif as C_1_-X-C_2_-X-C_3_C_4_-X-C_5_-L/I/V-C_6_-X-C_7_-X-C_8_ was manually scanned for all BrXYLPs [7,9]. Other conserved domains were also verified using the InterPro database. Subsequently, putative AG glycomodules were manually predicted following the criteria described before [3,8,16,80]. In brief, contiguous Pro residues, such as [Ala/Ser/Thr/Gly]-Pro_2-4_, are hydroxylated and mainly glycosylated by arabino-oligosaccharides (arabinosides), while non-contiguous Pro residues as [Ala/Ser/Thr/Gly]-Pro-X(0,10)-[Ala/Ser/Thr/Gly]-Pro (two consecutive Pros are not separated by more than 11 amino acid residues) are sites of galactosylation. Those candidates that had putative AG glycomodules were finally defined as AGPs. Deduced amino acid sequences of putative AGPs were submitted to SignalP 5.0 Server (https://services.healthtech.dtu.dk/service.php?SignalP-5.0, accessed on 1 December 2021) to predict the presence of N-terminal signal peptides. The prediction of transmembrane helices in proteins was based on TMHMM Server v. 2.0 (https://services.healthtech.dtu.dk/service.php?TMHMM-2.0, accessed on 1 December 2021). GPI anchor addition sequences were predicted using the GPI-SOM (http://gpi.unibe.ch, accessed on 1 December 2021) and the BIG-PI Plant Predictor (http://mendel.imp.ac.at/gpi/plant_server.html, accessed on 1 December 2021). NetNGlyc 1.0 Server (https://services.healthtech.dtu.dk/service.php?NetNGlyc-1.0, accessed on 1 December 2021) was used to check the presence of N-glycosylation sites. ProtParam tool (http://web.expasy.org/protparam/, accessed on 1 December 2021) was utilized to compute the amino acid composition of BrAGP protein sequences. Pair-wise sequence alignment of full-length sequences was performed using the Align tool (http://www.ebi.ac.uk/, accessed on 1 December 2021). Identification of the *cis*-acting elements in the promoter sequence was performed using PlantCare (http://bioinformatics.psb.ugent.be/webtools/plantcare/html, accessed on 1 December 2021). The protein subcellular localization was assumed by WoLF PSORT (https://psort.hgc.jp/, accessed on 1 December 2021). Additional information on AGPs was obtained from the Arabidopsis Information Resource (TAIR, https://www.arabidopsis.org/, accessed on 1 December 2021), BRAD and National Center for Biotechnology Information Resource (NCBI, http://www.ncbi.nlm.nih.gov, accessed on 1 December 2021).

### 4.3. Phylogenetic Analysis

Multiple alignments of AGP protein sequences in Arabidopsis and *B. rapa* were generated using Clustal X version 2.0 [81]. Phylogenetic trees were constructed using a neighbor-joining algorithm and drawn using MEGA X software. Bootstrapping was implemented with 1000 replicates. Then, the phylogenetic trees were visualized with the OmicStudio tools at https://www.omicstudio.cn/tool/ (accessed on 1 December 2021). Multiple Em for Motif Elicitation (MEME) (https://meme-suite.org/meme/tools/meme, accessed on 1 December 2021) was used for motif discovery and searching [82].

### 4.4. Chromosomal Localization

The 24 conserved collinear blocks on the ten chromosomes of the *B. rapa* genome and the five chromosomes of the Arabidopsis genome were based on previous studies [83,84]. These blocks are labeled A to X and are color coded by inferred ancestral chromosomes following established convention [85]. The position on the blocks and the chromosomal location of each *AGP* gene were obtained from TAIR and BRAD databases. The tool for syntenic gene analysis, SynOrths, embedded in the BRAD database (http://brassicadb.cn/#/syntenic-gene/, accessed on 1 December 2021), was used to check the duplication of *BrAGP* genes by searching for homologous genes between Arabidopsis and three *B. rapa* subgenomes (LF, MF1 and MF2) [58]. The syntenic relationships were illustrated by the OmicStudio tools at https://www.omicstudio.cn/tool/ (accessed on 1 December 2021).

### 4.5. Expression Analysis of BrAGP Genes

Gene expression abundance of 72,169 unigenes (uploaded to the Sequence Read Archive of NCBI; accession number SRP149066) showed by FPKMs was generated from our previous study using RNA sequencing [54]. Differentially expressed *BrAGP* genes were screened out using the DESeq (2010) R Package. A Benjamini–Hochberg false discovery rate (FDR) <0.05 and a log_2_ fold change (FC) ≥ 1 or ≤−1 in each pairwise comparison were assigned as criteria.

### 4.6. Amplification of Three BrFLA Genes

For PCR analysis, genomic DNA was extracted from fresh young leaves of ‘*Bcajh97-01B*’ according to Cetyl Trimethyl Ammonium Bromide [86]. Total RNA extracted from the whole inflorescence of ‘*Bcajh97-01B*’ using Trizol^®^ Reagent (Invitrogen, Shanghai, China) was transcribed into cDNA. DNA and cDNA fragments of *BrFLA2* (Bra001464), *BrFLA28* (Bra034746) and *BrFLA32* (Bra038741) were amplified by specific primers (Table S6) using Phanta^®^ Max Super-Fidelity DNA Polymerase (Vazyme, Nanjing, China) and verified by sequencing.

### 4.7. Expression Analysis of BrFLA2, BrFLA28 and BrFLA32

The promoter sequences of 1962 bp, 1957 bp and 1962 bp upstream from the first ATG of the coding open reading frame fragments of *BrFLA2*, *BrFLA28* and *BrFLA32* were cloned for the eGFP–GUS fusion with gene-specific primers (Table S6), respectively. The PCR product was cloned into the pBI101 vector with enhanced green fluorescent protein (GFP) and *β*-glucoronidase (GUS) and verified by sequencing. The plasmids were then transformed into Arabidopsis using the floral dip method via *Agrobacterium tumefaciens* [87]. Transformants were selected using 50 mg·L^−1^ kanamycin. The backgrounds of the transformants were verified by primers for *NPT II* (Table S6). GFP fluorescence and GUS signal were detected under an ECLIPSE 90i fluorescent microscope (Nikon, Tokyo, Japan).

For quantitative real-time PCR, RNA was extracted from floral buds at five developmental stages (stage 1: longitudinal diameter ≤1 mm, stage 2–5: longitudinal diameter approximately at 2.2, 2.6, 3.8 and 5.9 mm, respectively; stage 1–5 represent pollen mother cell stage, tetrad stage, uninucleate stage, binucleate stage and mature pollen stage) [88], roots, stems, young leaves, whole inflorescences, siliques, sepals, petals, stamens, gynoeciums and pistils at 1, 3, 10 and 24 HAP. First-strand cDNA was then synthesized using a PrimeScript^®^ RT reagent Kit with gDNA Eraser (TaKaRa, Beijing, China) as the template. *UBC10* was used as the normalization control. Quantitative real-time PCR was performed to analyze the expression level of *BrFLA2*, *BrFLA28* and *BrFLA32* with specific primers (Table S6) on a CFX96 Real-time RT-PCR Detection System (Bio-Rad, Hercules, CA, USA). Three technical repeats and three biological replicates were carried out. The values represent means ± standard deviation (SD) of three biological replicates. The relative expression levels were calculated by the 2^−ΔΔCt^ method [89].

For subcellular localization, signal peptide sequences and the rest fragments of *BrFLA2*, *BrFLA28* and *BrFLA32* full-length complementary DNA were cloned for the GFP fusion with specific primers (Table S6). The PCR products were cloned into the 3′- and 5′-terminuses of *GFP* in the pFGC vector and transiently transformed into onion epidermal cells by particle bombardment [90]. Twenty-four hours after introduction, onion epidermal cells were plasmolyzed in 0.3 g·mL^−1^ sucrose for 3 min; the GFP fluorescence of transgenic onion epidermal cells was observed under a LSM780 confocal microscope (ZEISS, Oberkochen, Germany).

### 4.8. Construction of RNAi Recombinant Vector and Plant Transformation

For the RNAi construct, a 518-bp specific sequence of *BrFLA32* was amplified and cloned in the sense orientation followed by a 357-bp inverted repeat to create a hairpin transgene into the pBI121 vector with the *NPT II* reporter gene. The recombinant vector was verified by sequencing and then transformed into Chinese cabbage by the *Agrobacterium tumefaciens*-mediated method [91]. A binary vector pBI121 was introduced into Chinese cabbage as a control. PCR screening approach was performed with *NPT II*-specific primers to confirm the RNAi-positive transformants. Quantitative real-time PCR was performed to detect the mRNA levels of *BrFLA2*, *BrFLA28* and *BrFLA32* in RNAi transformants and control plants according to the method mentioned above. All primers used are listed in Table S6. Afterward, positive transformants were cultivated in a growth chamber at 22 ± 2 °C under long-day conditions (16 h of light/8 h of dark) with 50% relative humidity. A parallel experiment was carried out at 85% relative humidity with other culture conditions unchanged.

### 4.9. Microscopy

Flowers and floral organs were photographed with a MZ16FA stereoscopic microscope (Leica, Wetzlar, Germany). Mature pollen grains were stained with Alexander’s solution (95% ethanol, 10 mL; 1% acid fuchsin, 5 mL; 1% orange G, 0.5 mL; glacial acetic acid, 2 mL; glycerol, 25 mL; phenol, 5g; distilled water, 50 mL) overnight [92], 1 μg·L^−1^ DAPI solution for 5 min [93] and calcofluor white solution for 5 min [94], respectively. Images of stained pollen grains were taken using an ECLIPSE 90i fluorescent microscope (Nikon, Tokyo, Japan). Pollen germination in vitro was performed in a medium containing 15% sucrose, 0.4 mmol·L^−1^ HBO_3_, 0.4 mmol·L^−1^ Ca(NO_3_)_2_, 0.1% agar, and the pH adjusted to 5.8. The pollen grains were grown at 22 °C with 100% relative humidity in the dark. Pollen tubes were photographed with an ECLIPSE 90i fluorescent microscope (Nikon, Tokyo, Japan). For scanning electron microscopic examination, fresh anthers and pollen grains were coated with gold–palladium in a Model IB5 ion coater (Eiko Engineering Company, Nagoya, Japan) and observed under a Model TM-1000 microscope (Hitachi, Tokyo, Japan).

## 5. Conclusions

We identified 293 *AGP* genes from the *B. rapa* genome and divided them into different subfamilies based on the differences in the domain constituents of their core protein backbone sequences: 25 classical AGPs, three Lys-rich AGPs, 30 AG-peptides, 36 FLAs, 59 PLAs, 33 XYLPs, 102 other CAGPs, two non-classical AGPs and three HAEs. On this basis, we further classified *BrAGP* genes into several distinct clades based on their evolutionary relationships, and elucidated their genomic characteristics, protein structures, duplication status and expression patterns during five different microspore developmental stages in the ‘*Bcajh97-01A/B*’ system. Alterations in *BrAGP* gene expression levels in at least one stage of the fertile floral buds compared with the sterile ones indicated that *BrAGP* genes may be involved in floral bud development. RNAi transgenic plants with marked down-regulation of *BrFLA2*, *BrFLA28* and *BrFLA32* expression showed early germinated pollen tubes in the anthers under high humidity, suggesting the implication of *BrFLAs* in pollen ontogenesis. In conclusion, our study has provided insights into the characteristics of *BrAGP* genes and is a first step in the functional research of *BrAGP* genes.

## Figures and Tables

**Table 1:** List of differentially expressed *BrAGP* genes in floral buds from male-sterile ‘*Bcajh97-01A*’and male-fertile ‘*Bcajh97-01B*’ of *Brassica rapa*.

Subfamily	Gene Name	BRAD Locus	DEGs ID in [54]	BS1/AS1	BS2/AS2	BS3/AS3	BS4/AS4	BS5/AS5
AG-peptide	*BrAGP12.2*	Bra039397	c42093.graph_c0	.	.	.	−3.2	1.4
AG-peptide	*BrAGP16.1*	Bra004546	c59251.graph_c1	.	.	.	−2.2	.
AG-peptide	*BrAGP16.2*	Bra000419	c65782.graph_c0	1.2	.	1.9	1.1	2.8
AG-peptide	*BrAGP22.1*	Bra003071	c48812.graph_c0	.	.	.	−2.7	2.7
AG-peptide	*BrAGP23.2*	Bra014611	c27422.graph_c1	1.5	.	F	9.0	F
AG-peptide	*BrAGP24*	Bra025551	c49943.graph_c0	F	F	F	4.5	3.4
AG-peptide	*BrAGP40.2*	Bra023919	c42971.graph_c0	1.1	F	F	5.8	F
AG-peptide	*BrAGP46*	Bra008765	c37395.graph_c0	F	F	2.5	2.6	F
Classical	*BrAGP1.1*	Bra024284	c48286.graph_c0	.	−1.0	−1.1	.	−2.4
Classical	*BrAGP1.2*	Bra031924	c64744.graph_c0	.	.	.	−1.3	−2.3
Classical	*BrAGP2.1*	Bra038521	c26555.graph_c0	−1.3	.	.	1.2	−1.5
Classical	*BrAGP6*	Bra008762	c23263.graph_c0	.	F	F	2.6	6.7
Classical	*BrAGP10.2*	Bra000670	c42352.graph_c0	.	.	.	−1.8	−3.1
Classical	*BrAGP11.2*	Bra040548	c24412.graph_c1	1.1	S	.	7.5	9.4
Classical	*BrAGP27*	Bra040224	c23415.graph_c0	.	1.4	.	2.3	−2.0
Classical	*BrAGP50.3*	Bra012505	c54741.graph_c0	.	.	S	.	F
Classical	*BrAGP54.1*	Bra011948	c46468.graph_c0	.	.	1.6	F	F
Lys-rich	*BrAGP17*	Bra039184	c44058.graph_c0	1.7	.	2.6	5.6	.
Non-classical	*BrAGP58.1*	Bra040103	c50741.graph_c1	.	.	.	1.8	−1.8
Non-classical	*BrAGP58.2*	Bra021074	c48178.graph_c0	.	.	.	1.0	−2.0
FLA	*BrFLA1*	Bra000566	c48483.graph_c1	.	.	F	1.4	8.7
FLA	*BrFLA2*	Bra001464	c51893.graph_c0	F	F	F	6.5	12.1
FLA	*BrFLA8*	Bra005920	c62616.graph_c0	1.1	F	F	.	S
FLA	*BrFLA13*	Bra010241	c28700.graph_c0	F	F	6.1	F	F
FLA	*BrFLA19*	Bra025535	c23062.graph_c0	.	1.7	.	.	.
FLA	*BrFLA24*	Bra029925	c47364.graph_c0	.	.	1.1	.	−2.2
FLA	*BrFLA25*	Bra032093	c48483.graph_c0	.	S	F	F	F
FLA	*BrFLA28*	Bra034746	c56550.graph_c0	.	.	8.2	8.4	11.4
FLA	*BrFLA32*	Bra038741	c56550.graph_c0	.	.	8.2	8.4	11.4
FLA	*BrFLA36*	Bra023589	c28344.graph_c0	−2.9	2.2	.	−3.6	1.5
PLA	*BrENODL3*	Bra001712	c42235.graph_c0	F	1.1	8.9	F	F
PLA	*BrENODL7*	Bra003532	c57139.graph_c0	S	.	3.3	6.0	.
PLA	*BrENODL28*	Bra022335	c27217.graph_c0	F	F	F	F	F
PLA	*BrENODL32*	Bra023981	c26115.graph_c0	.	.	.	.	−3.7
PLA	*BrENODL42*	Bra032131	c23242.graph_c0	S	5.6	−4.3	F	S
PLA	*BrENODL43*	Bra033326	c59532.graph_c0	.	1.0	−4.0	F	F
PLA	*BrENODL49*	Bra037575	c40690.graph_c0	F	F	F	F	F
PLA	*BrSCL1*	Bra002283	c25660.graph_c0	−1.4	−1.7	.	−1.7	6.3
PLA	*BrSCL5*	Bra020092	c48577.graph_c1	F	F	F	3.4	F
PLA	*BrUCL1*	Bra000364	c40349.graph_c0	−2.4	.	−2.2	−4.7	−3.9
PLA	*BrUCL7*	Bra009259	c37087.graph_c0	2.3	−1.9	F	S	S
PLA	*/*	Bra019044	c24852.graph_c0	.	.	.	−2.3	.
XYLP	*BrXYLP3*	Bra013135	c53024.graph_c0	.	−1.4	.	2.0	−2.1
XYLP	*BrXYLP5*	Bra000652	c27389.graph_c0	F	2.3	F	7.1	8.9
XYLP	*BrXYLP9*	Bra036905	c24892.graph_c0	1.1	F	1.9	.	−5.7
XYLP	*BrXYLP13*	Bra001874	c46072.graph_c1	F	F	S	S	S
XYLP	*BrXYLP15*	Bra025919	c28506.graph_c0	F	2.5	7.7	7.4	F
XYLP	*BrXYLP16*	Bra016563	c64352.graph_c0	.	2.7	.	10.4	10.5
XYLP	*BrXYLP17*	Bra031024	c41112.graph_c0	F	F	F	F	10.4
XYLP	*BrXYLP21*	Bra032857	c64539.graph_c0	.	.	.	−3.3	1.9
XYLP	*BrXYLP23*	Bra021455	c51308.graph_c0	.	4.8	−7.0	−1.7	−3.0
XYLP	*BrXYLP25*	Bra001875	c36844.graph_c0	F	1.7	.	−6.0	−5.2
XYLP	*BrXYLP27*	Bra032462	c64575.graph_c0	F	F	3.0	−3.9	−2.2
XYLP	*BrXYLP29*	Bra025907	c23808.graph_c0	.	−1.8	F	1.6	2.0
CAGP	*BrCAGP2*	Bra039574	c59222.graph_c0	F	F	F	F	F
CAGP	*BrCAGP7*	Bra014398	c54415.graph_c0	F	2.7	−2.0	1.0	S
CAGP	*BrCAGP9*	Bra001983	c55708.graph_c0	.	1.1	−2.4	1.0	−1.2
CAGP	*BrCAGP13*	Bra010330	c62315.graph_c3	.	.	.	1.4	1.7
CAGP	*BrCAGP24*	Bra040283	c67220.graph_c0	F	1.1	−2.4	F	8.7
CAGP	*BrCAGP28*	Bra036815	c9352.graph_c0	F	F	F	F	F
CAGP	*BrCAGP33*	Bra039561	c60389.graph_c0	.	.	1.5	1.4	3.7
CAGP	*BrCAGP37*	Bra037087	c53995.graph_c0	S	F	F	.	7.2
CAGP	*BrCAGP55*	Bra011048	c56297.graph_c0	2.1	3.7	S	−2.5	2.3
CAGP	*BrCAGP67*	Bra022410	c58601.graph_c0	.	2.1	.	.	.
CAGP	*BrCAGP71*	Bra035635	c50787.graph_c0	1.6	.	1.2	−2.6	2.9
CAGP	*BrCAGP73*	Bra021861	c52042.graph_c0	.	.	.	−1.9	−1.7
CAGP	*BrCAGP81*	Bra026880	c47549.graph_c0	.	F	.	−1.8	−2.6
CAGP	*BrCAGP88*	Bra036147	c55619.graph_c0	F	F	F	5.1	4.1
CAGP	*BrCAGP92*	Bra001249	c53604.graph_c0	.	.	1.3	1.6	.
CAGP	*BrCAGP94*	Bra006651	c55481.graph_c0	2.0	.	−3.4	.	−1.7
CHAE	*BrCHAE1*	Bra013339	c43369.graph_c0	.	1.3	1.4	.	.
HAE	*BrHAE1*	Bra030020	c64968.graph_c0	S	F	2.2	1.0	F
HAE	*BrHAE3*	Bra014023	c56215.graph_c0	−1.5	3.1	1.7	5.1	.

All values are expressed in terms of the ratio of male-fertile floral buds to male-sterile floral buds, so that positive values indicate depression of gene expression in male-sterile floral buds. Dots represent either no difference or no expression. *A*, homozygous male-sterile line ‘*Bcajh97-01A*’; *B*, heterozygous male-fertile line ‘*Bcajh97-01B*’; *S1*–*S5*, pollen mother cell stage, tetrad stage, uninucleate stage, binucleate stage and mature pollen stage; *S*, specifically expressed genes in the floral buds of homozygous male-sterile line ‘*Bcajh97-01A*’; *F*, specifically expressed genes in the floral buds of heterozygous male-fertile line ‘*Bcajh97-01B*’.

## Data Availability

The data presented in this study are openly available in the Sequence Read Archive of NCBI, accession number SRP149066 [54].

## Supplementary Materials

The following are available online at https://www.mdpi.com/article/10.3390/ijms222313142/s1.
