# Supplementary material for: Comprehensive Analysis of Arabinogalactan Protein-Encoding Genes Reveals the Involvement of Three BrFLA Genes in Pollen Germination in Brassica rapa
Source: Int J Mol Sci. 2021 Dec 5;22(23):13142. doi: 10.3390/ijms222313142 (PMC8658186; doi:10.3390/ijms222313142)
Supplement: Supplementary file 1 [file ijms-22-13142-s001.zip › ijms-1482427-supplementary/Figures S1-S10.pdf]

International Journal of
Molecular Sciences

MDPI

Supplementary Material

Comprehensive Analysis of Arabinogalactan Protein-Encoding Genes Reveals the Involvement of Three *BrFLA* Genes in Pollen Germination in *Brassica rapa*

Huiting Huang [1,†], Yingjing Miao [1,2,†], Yuting Zhang [1], Li Huang [2], Jiashu Cao [2,*] and Sue Lin [1,3,*]

[1] Institute of Life Sciences, College of Life and Environmental Science, Wenzhou University, Wenzhou 325000, Zhejiang, China
[2] Laboratory of Cell & Molecular Biology, Institute of Vegetable Science, Zhejiang University, Hangzhou 310058, Zhejiang, China
[3] Biomedicine Collaborative Innovation Center of Zhejiang Province, Wenzhou University, Wenzhou 325000, Zhejiang, China
* Correspondence: jshcao@zju.edu.cn (J.C.); iamkari@163.com (S. L.)
† The first two authors contributed equally to this work.

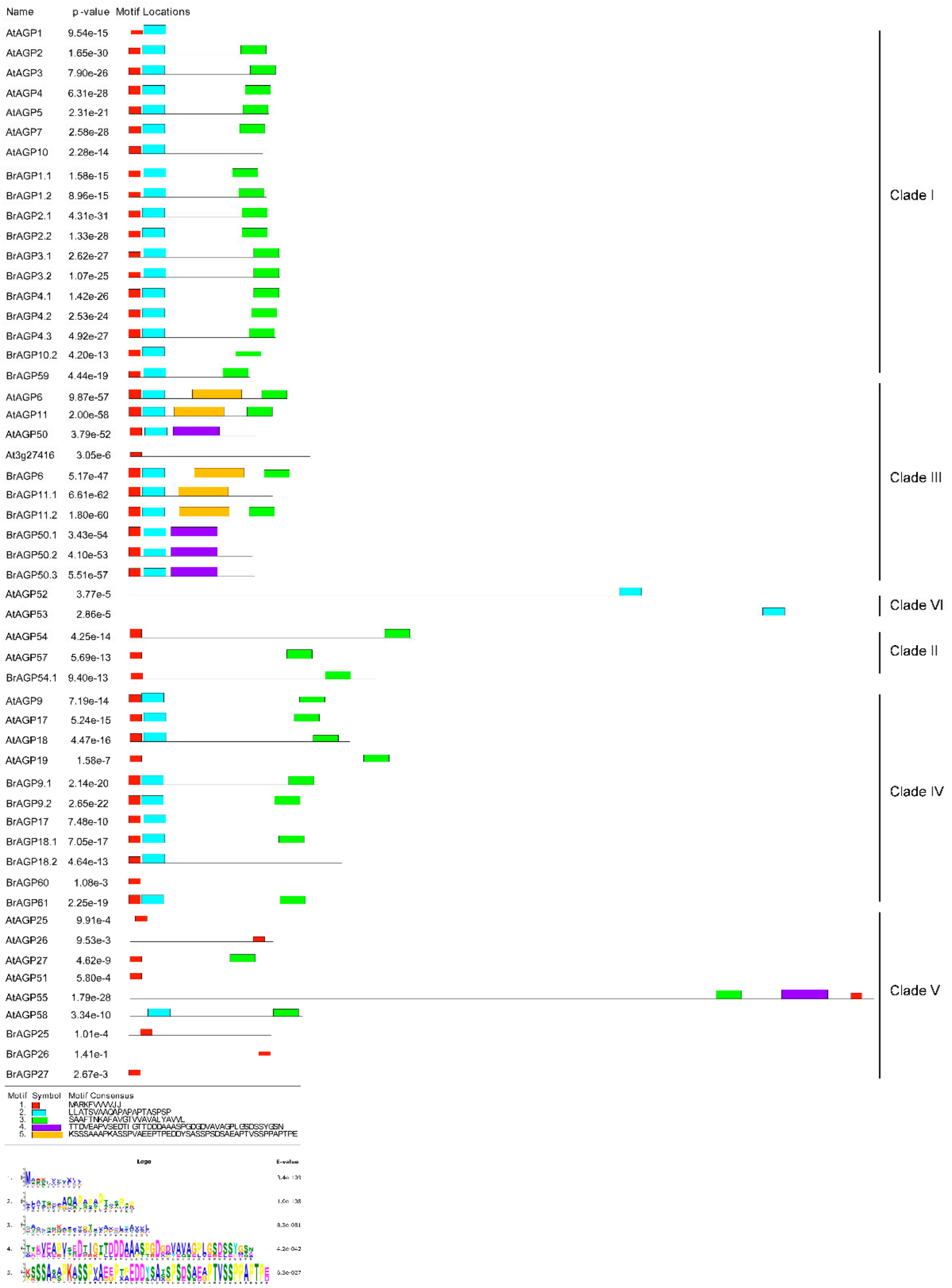

Figure S1. Motif analysis of classical AGPs and Lys-rich AGPs from *Brassica rapa* and Arabidopsis. Motif discovery and searching was performed by Multiple Em for Motif Elicitation (MEME) software.

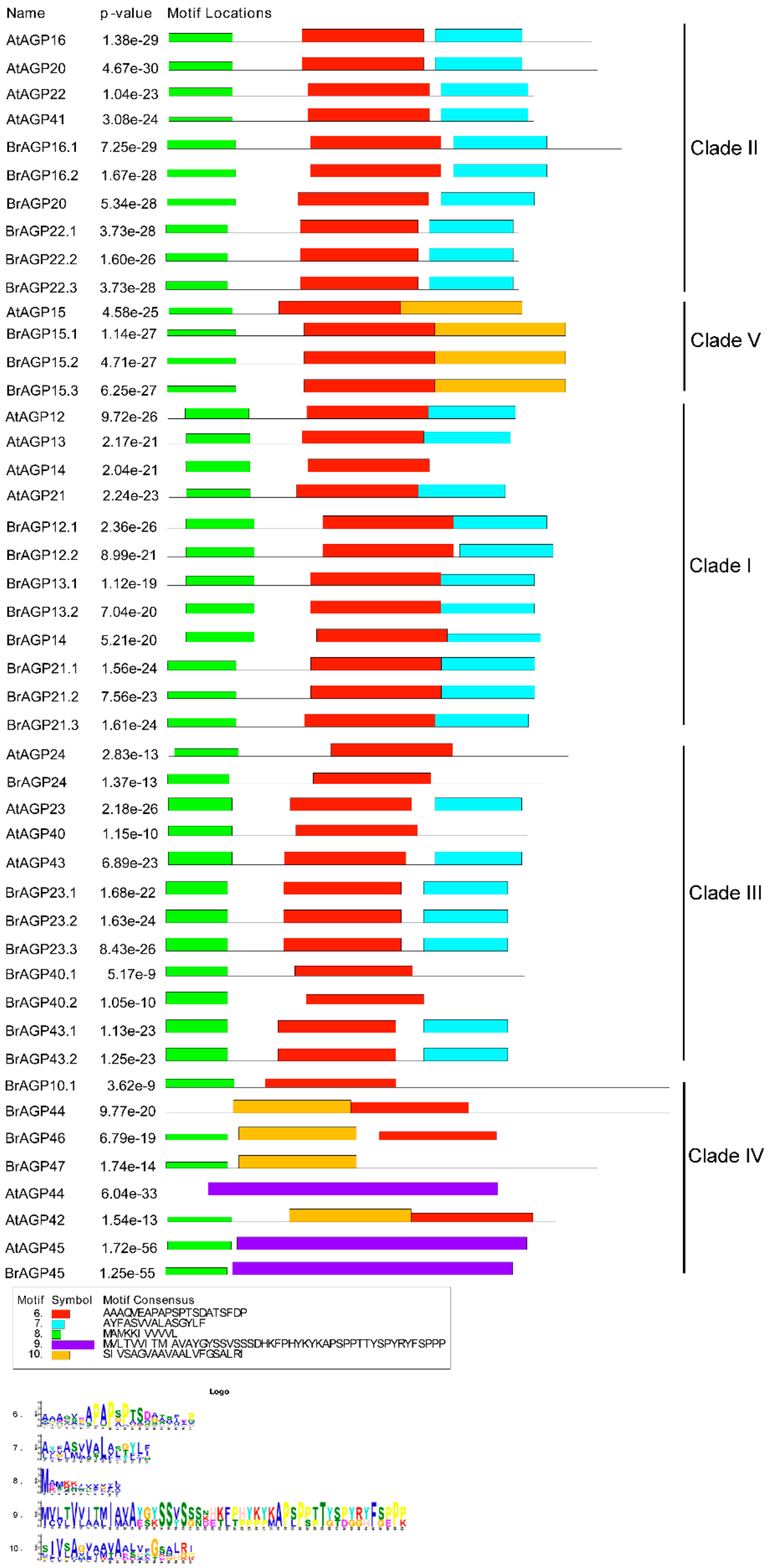

Figure S2. Motif analysis of AG-peptides from *Brassica rapa* and Arabidopsis. Motif discovery and searching was performed by Multiple Em for Motif Elicitation (MEME) software.

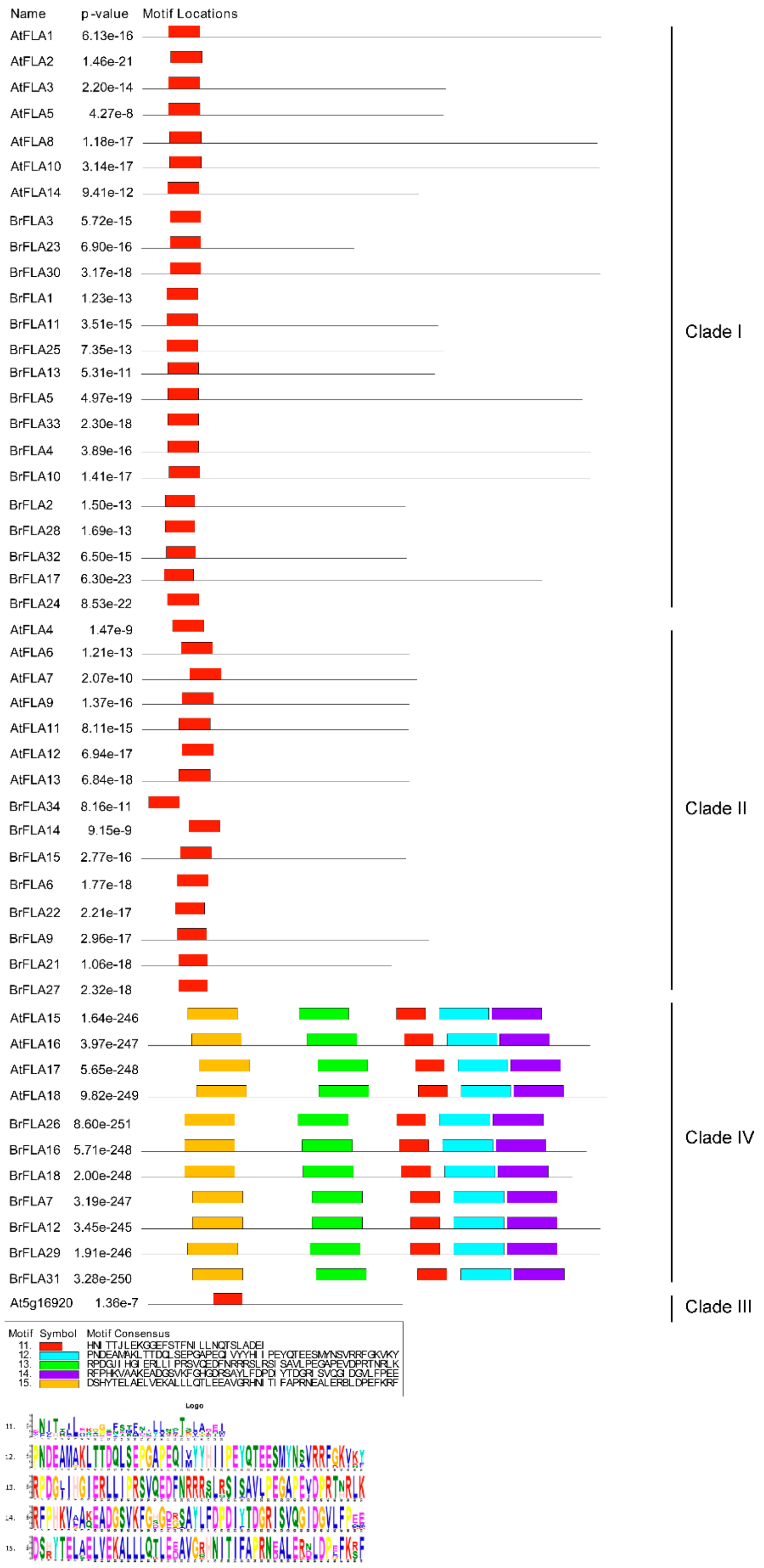

Figure S3. Motif analysis of fasciclin-like AGPs (FLAs) from *Brassica rapa* and Arabidopsis. Motif discovery and searching was performed by Multiple Em for Motif Elicitation (MEME) software.

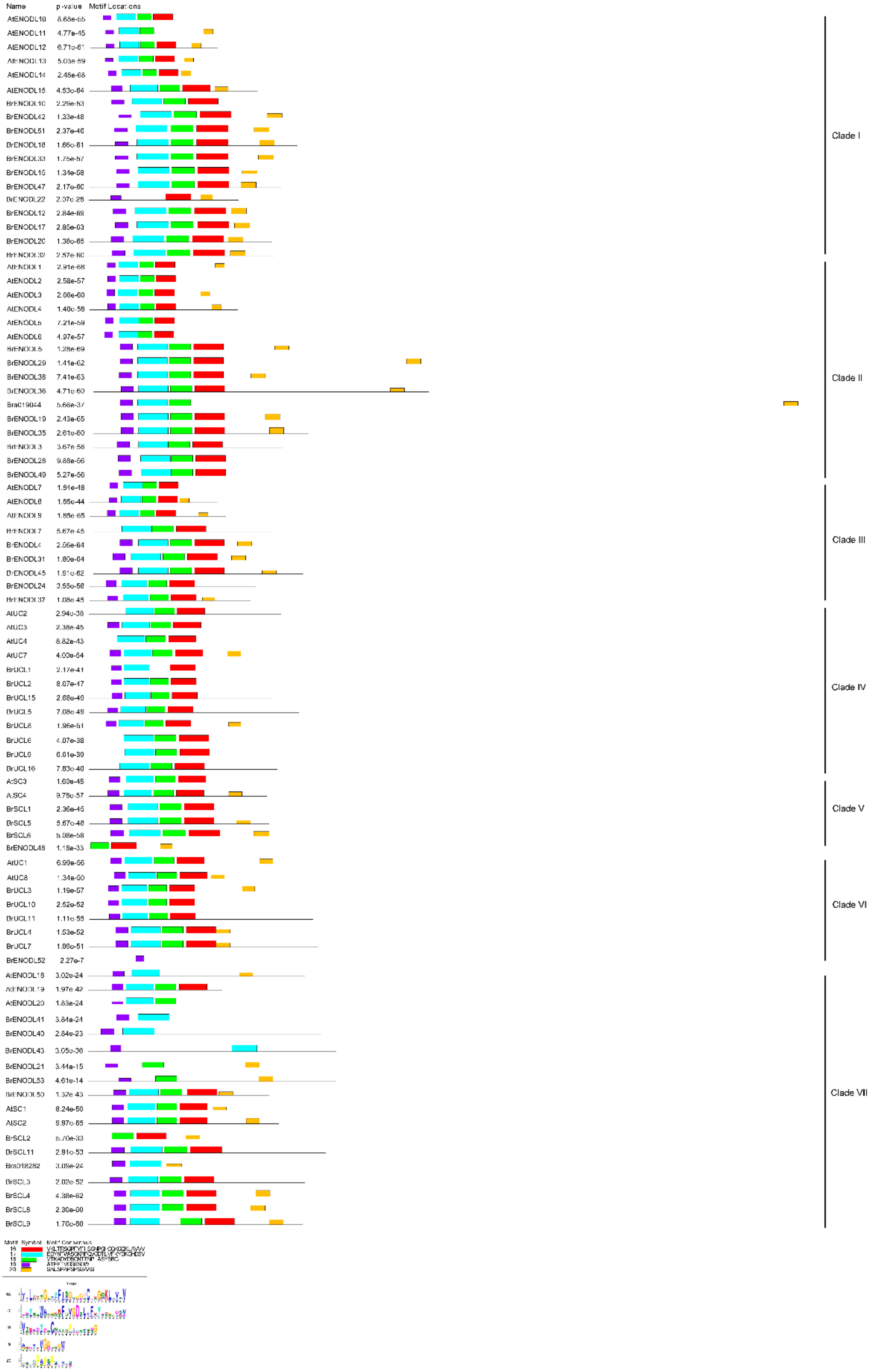

Figure S4. Motif analysis of phytocyanin-like AGPs (PLAs) from *Brassica rapa* and Arabidopsis. Motif discovery and searching was performed by Multiple Em for Motif Elicitation (MEME) software.

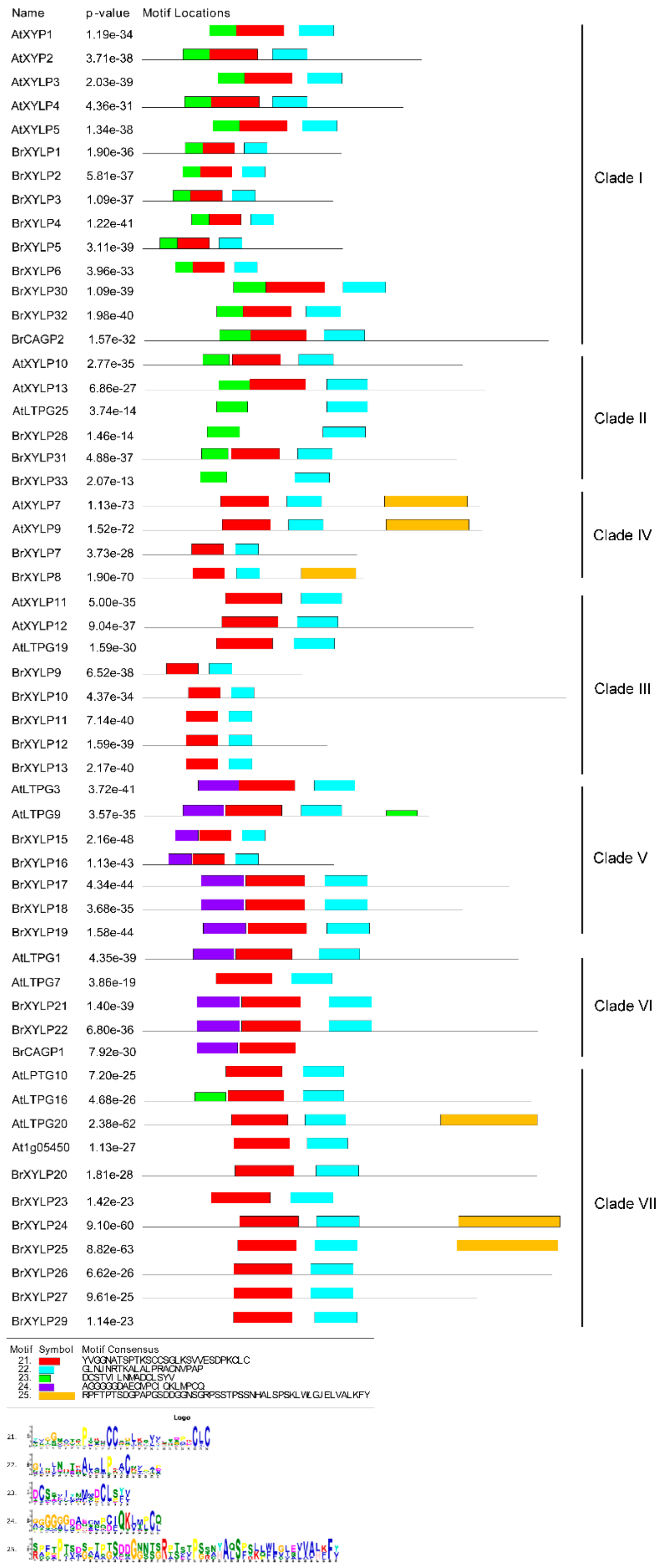

Figure S5. Motif analysis of xylogen-like proteins (XYLPs) from *Brassica rapa* and Arabidopsis. Motif discovery and searching was performed by Multiple Em for Motif Elicitation (MEME) software.

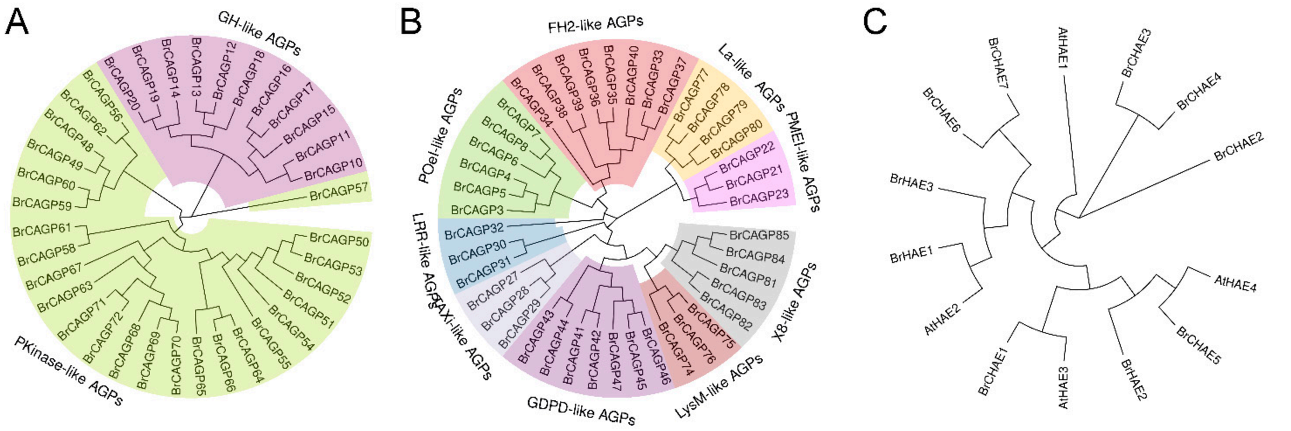

Figure S6. Phylogenetic tree representation of chimeric AGPs (CAGPs) and AGP/extensins hybrids (HAE) from *Brassica rapa* (BrAGPs) and Arabidopsis (AtAGPs). (**A**,**B**) BrCAGPs with conservative domains. (**C**) Chimeric HAEs (CHAEs) and HAEs. The Neighbor-Joining tree was constructed using MEGA X software with 1,000 bootstrap. The OmicStudio tools was used to display and manipulate trees.

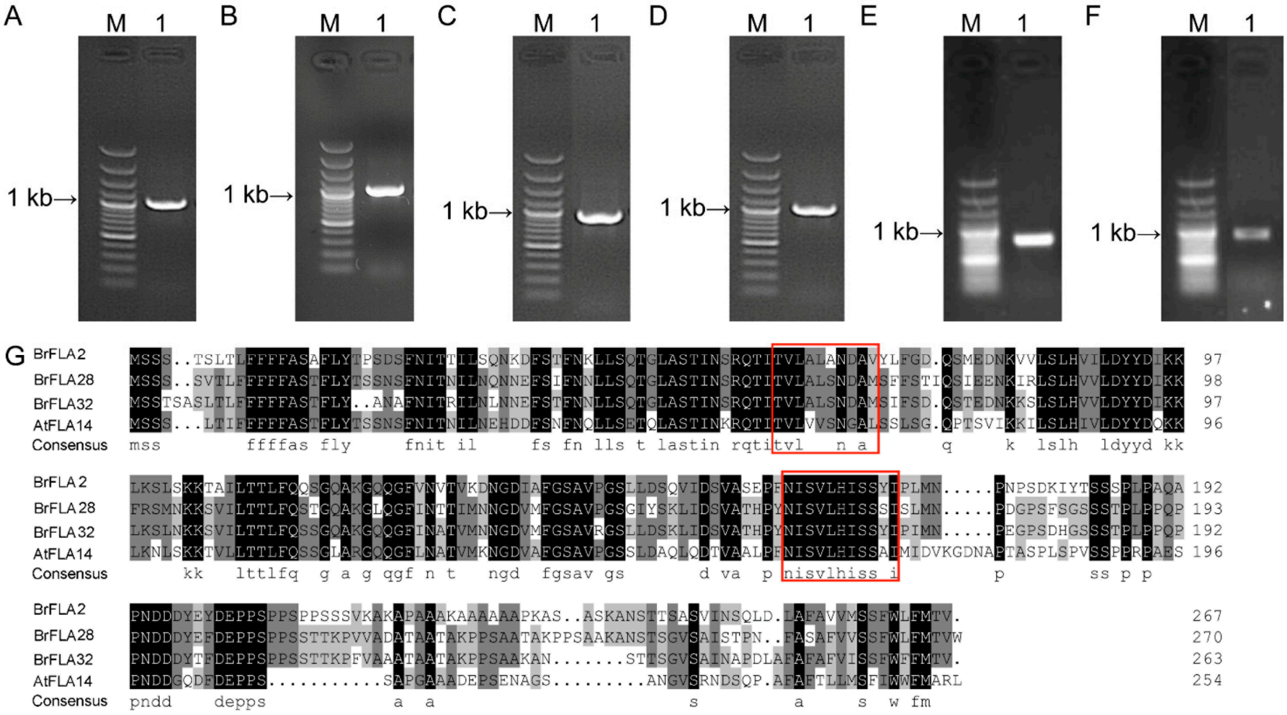

Figure S7. PCR amplification the full-length DNA and cDNA sequences of *BrFLA2*, *BrFLA28* and *BrFLA32*. (**A**,**C**,**E**) Full-length DNA amplification of *BrFLA2* (**A**), *BrFLA28* (**C**) and *BrFLA32* (**E**). (**B**,**D**,**F**) cDNA amplification of *BrFLA2* (**B**), *BrFLA28* (**D**) and *BrFLA32* (**F**). Lane M indicates the molecular marker. (**G**) Multiple alignments of BrFLA2, BrFLA28, BrFLA32 and AtFLA14 protein sequences. Red squares indicate the conserved motifs H1 and H2 of fasciclin domains.

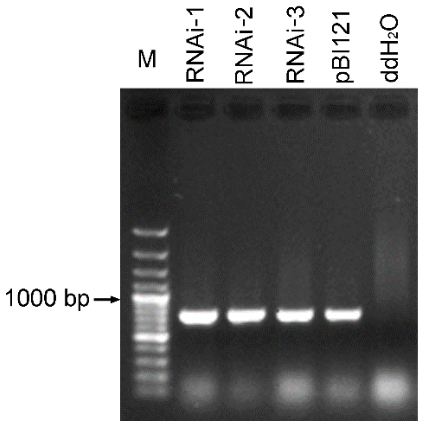

Figure S8. Genomic DNA PCR analysis of *BrFLA2/28/32*–RNAi transgenic plants in *Brassica rapa*. A 740-bp expected band was amplified in three *BrFLA2/28/32*–RNAi transgenic lines and the binary vector pBI121. Lane M indicates the molecular marker.

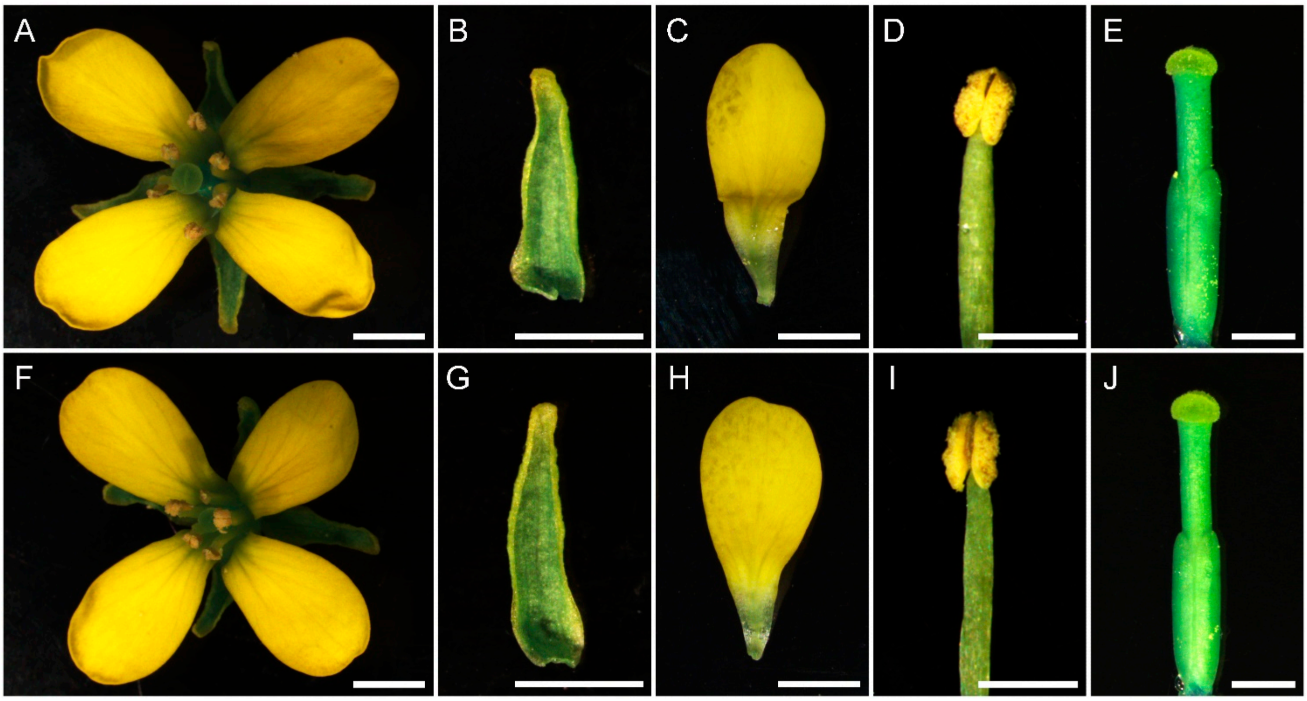

Figure S9. Morphological features of flowers from *BrFLA2/28/32*–RNAi transgenic plants in *Brassica rapa*. (**A**–**E**) A floret (**A**), a sepal (**B**), a petal (**C**), a stamen (**D**) and a pistil (**E**) at anthesis stage in a *BrFLA2/28/32*–RNAi plant. (**F**–**J**) A floret (**F**), a sepal (**G**), a petal (H), a stamen (**I**) and a pistil (**J**) at anthesis stage in a control plant. Scale bars = 2 mm.

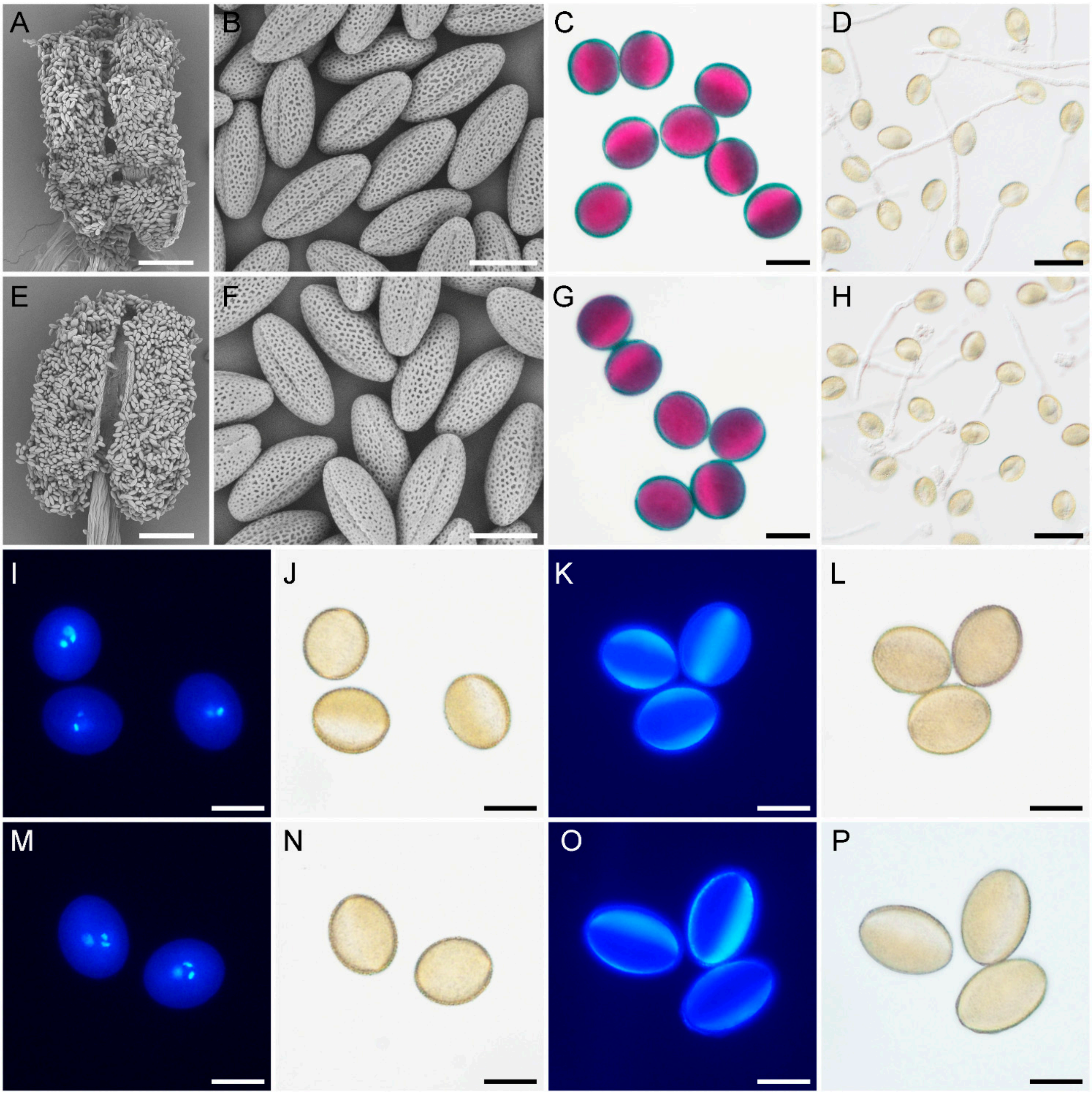

Figure S10. Mature pollen analysis in *BrFLA2/28/32*–RNAi transgenic plants in *Brassica rapa*. (**A**,**B**,**E**,**F**) Scanning electron microscopy observation of dehiscent anthers (**A**,**E**) and mature pollens (**B**,**F**) in *BrFLA2/28/32*–RNAi (**A**,**B**) and control plants (**E**,**F**). (**C**,**G**) Alexander staining of mature pollens in *BrFLA2/28/32*–RNAi (**C**) and control plants (**G**). (**D**,**H**) *BrFLA2/28/32*–RNAi (**D**) and control (**H**) pollen grains germinated *in vitro* for 4 h. (**I**,**M**) DAPI staining of mature pollens in *BrFLA2/28/32*–RNAi (**I**) and control plants (**M**). (**J**,**N**) The corresponding bright field images of (**I**,**M**). (**K**,**O**) Calcofluor fluorescent white staining of mature pollens in *BrFLA2/28/32*–RNAi (**K**) and control plants (**O**). (**L**,**P**) The corresponding bright field images of (**K**,**O**). Scale bars = 200 μm (**A**,**E**); 50 μm (**D**,**H**); 20 μm (**B**,**C**,**F**,**G**,**I**–**P**).
