# Supplementary material for: Comprehensive Analysis of Arabinogalactan Protein-Encoding Genes Reveals the Involvement of Three BrFLA Genes in Pollen Germination in Brassica rapa
Source: Int J Mol Sci. 2021 Dec 5;22(23):13142. doi: 10.3390/ijms222313142 (PMC8658186; doi:10.3390/ijms222313142)
Supplement: Supplementary file 1 [file ijms-22-13142-s001.zip › ijms-1482427-supplementary/Table S4.pdf]

Table S4 The protein backbones of newly identified BrAGPs.

Gene name	BRAD locus [a]	Domains	Protein backbones
BrAGP46	*Bra008765*	-	MAARSSLIRFAFVCIVLAVLVMTAESHNGVNHGPAKSPSSHDPKAHAPAPSAATFSAYPQ LIATALVGALSFVF
BrAGP47	Bra023457	-	MAARSSLVRFAFVCIVLAVLVMTAESHAGHHHGPAMAPGMAPMPHHHTPAPAPSGATFS AYPQLIATALVGALSFVF
BrFLA35	Bra007193	FAS	MRRLLIKKNPVFFLFLLITTSSLTILIFSLLRLPDSPPIIFTGKLRADLSDELGFFGNMIIEML PEDLVFTAFVPSEKAFDRDLGMTKPNNTRKSKSLEDEENTYAVVSRILGFAVVPYRVEAG DVGKDETASYESLSGFTLKIWRKSGGLVVNGVETEKMGLKRGKIMVHIMDGVIMDSDF AQSVASTDTTPQDEEEPKP
BrFLA34	Bra018207	FAS	MSPPPAGINLTQILINGHNFNVALSLLVASGVITELENDDHGAGITVFVPTDSAFSDLPENQ NLQSLPADKKAIVLKFHVLNSYYTLGSLESITNPVNPTLATELMGAGSYTLNISRVNGSIV TINSGLVLALVTQTAFDQNPVSVFGVSKVLLPKELFPKSGQPVSTPATTTPPRQVSLSPEG SDDQPSRLVAPPGEVVSSSTVKRTRVFFYLCWCIAFWCAFLV
BrFLA36	*Bra023589*	FAS	MRAIQTYVTLFFILVIITTTSTGAQKSPHHGNNHDLSIAIEEMEKANYFSFVMLINMLHST NPRLLANITFLMPRDKPLSRSNIIQQDSISDFLLRHSIPSPLLFEHLNLIPNGSIVPSSLPHYT LKITNGGRLNYFLNNVKIISRNICSLGSIKCHGIDGILPSPSAINDDSPRDNHTSPFISCPSSH NNSEHDSPHDNSEHDSPLNNSSNHNSSRPDDHTHTHVAPSPTSSPTLVPKSDSSTIREGDT

			SGFVVLLGLLSCVMGIAMAM
BrENODL7	*Bra003532*	plastocyanin-like (PCNL) domain	MVVVASEGPRVFKVGDEFEWRVPLQNDTSVYSRWANTNRFHIGDSLSFVYDKDSVMEVDKWGFYHCNGSDPITAFDNGNSTFYLDRPGLFYFISGSNAHCTSGQRLIVEVMHIHHHHNHHDVSLPPSMSPLSASSPSESAFDSHDPASSAAASSLLASFFPPFAALLVALFSCQP
BrENODL19	*Bra010371*	PCNL	MGLVKSFDAYLMIVMLTGLVFALGLSNGYKFYVGGKDGWVLTPSEDYSHWSHRNRFQVNDTLYFKYPKGKDSVLEVSEEEYNTCNTTHPITSLSDGDSLFVLSRSGPFFFVSGNSENCLKGQKLAVNVMSTAHHSRSPRQPSPSPSPTLSPIAWSSPAPSPGVVLSDSEALAPAPEPAKARNSASLVGPGMVSLGLVLVVFIRSMV
BrENODL35	*Bra026261*	PCNL	MGLIKRFDAYLMTVMLMSLGFAIGFSNGYKFYVGGRDGWVLTPSEDYSHWSYRSRFQVNDTLYFKYPKGKDSVLEVSEEEFNICNTTHPITSLTDGDSLYVLSRSGPFFFVSGNSENCLKGQKLPVKVMSAAHHSHSPRQPSPSPSPSPTLSPSHQALSSPAPSPRVVLSESEALAPAPGPAKAHNSAGLVSPRAVSLGLVLVVIISFVL
BrENODL40	Bra030730	PCNL	MSLMVLSLSADAYKNYTVGESKGWFDIQERPSVNYQKWADSKSFSLGDFLIFNTDSNHSVVQTYDYKTYKSCDYNNNEDNSTREWSAAKPSATSPVPVSVKVPLVKEGSNYFFSGNYDGEQCKFGQHFMINVTHGQGLPSPDEDDETAPGPGQSSQSGDDDEVAPDTIVPANFDHPKDIESDDDDSLVKGRKNSSCIAKYNLVCLVFMGFLASFF
BrENODL48	Bra036574	PCNL	MRVTHAMYRSCNNSNPISTFTTGNDSVTLTNHGHHFFCGVPGHCMAGQKLDLNVIHPISSTPLSDPPISSSSPPSTTTIPAAGVPGPSPSHAASLPSAAAAVVYLLVSLIFANSAS
BrENODL52	Bra041119	PCNL	MGSNHSSGSGSSPWSGWGPNNGQNTGSGGSGSGWGWGPKNTNNSGSGSSGSGWGWG

			WGSHSKGYNATYNAPRKFIVGGDKEWTYGFNYSDWASKTAPFFLNDILVFKYNPPAPFTHSVYLFSNPLSYEKCDVKKGKMIASPKQGAGNGFELVLTKMKPYYISCGEHDGAHCSNGTMKFTVMPILARW
BrUCL4	*Bra005941*	PCNL	MEKTSKKLFIFNLCINFGILVTRRCNATTYFVGDTSGWDISSDLESWPLGKRFSVGDVLMFQYSSSHSVYEVAKDNFQSCNTTDPIRTFTNGNTTVALSKPGDRFFVCGNRLHCFAGMRLQVNVQGNGPSPAPVGAPRAAPAGILQPSSKKNNPATGVASSAAHIGGRGLRGSMNYFVYLMVFTFPLILYFINN
BrUCL7	*Bra009259*	PCNL	MGKTSTILFLFYLCIIFGISVITRCNATTYFVGDTSGWDISSDLESWTLGKRFSVGDVLMFQYSSTHSVYEVAKDNFQSCNSTDPIRTFTNGNTTVALSKPGDRFFLCGNRLHCFAGMRLQVNVEGNGPSPSPVGAPGAAPVGILQPSSKKNNPPTGVATSSAPHVGGCGGRVSIVTFVYLMVFGFPLLWTHILVTTKIRVYSIQCMHTYMYWFDYVKLWFGLV
BrSCL2	*Bra007734*	PCNL	MAMGMFGESLASSSLHKFHNVKQVSRRDFLSCNATSALATYNSGSDTVALKNPGHYYFLCGFPGHCQAGQKLHVLVVATSTASPSLSPDLSPAPSPSIGSSPSPAQATASDDSAQNGALSVSLSTAMSGVVVFVVAFNLY
- (divided into unknown PCNL-containing protein	Bra018282	insufficiently long PCNL	MALIKNKSLFASFMIFVALFVGGTVHNVGDTKGWTMMGVDYEAWASSRTFQVRDSLIFENNKDYHDDKTSVPASLGPAAAPVPRQVRSPSLSLSPLPDSTVNNGPRYQKKPSRVPHSAASNSSVWIGSYILISLFPFFILF

subfamily)			
BrENODL53<br><br>(divided into BrENODL subfamily)	*Bra025873*	PCNL	MVSSQQRLSFSSSKLVFFFCILSLFSRPSLSASFLVDGVSVWKTPVVHVGDSVIFRHKYGNDLYIFRTKDAFNVCDFTQATLLTKSNSTSFTWYPSRPGSYYFSFTNNTSLPKTCQLSQKLTVQVLLAAASWPSQPPTPAIAPGPVSEGGDVSSSPSYPWPLGPREGSALSPGPSPSEITSVTVPGKDGVPFINSNPAVPLPTGEVDSTSINPLPTSTNSAHQVMMTVTVKLVLCCVAMFLLL
BrXYLP1	*Bra024265*	non-specific lipid transfer protein 2 (nsLTP2) domain	MATSFSAATPFVFILLLSISSVTVHGASHHHTAAPAPAVDCSTLIINMADCLDFVMAGGTSAKPKSSCCAGLKTVLKADAECLCEAFKNSAAFGITLNMTKAATLPTACKLHAPSISNCGLSMTPTMAPGLAPGGAVAAGPGAAGTTLAPTPSQGNDGSSLIPISFTTLFSALFFVLFLSRV
BrXYLP2	*Bra031935*	nsLTP2	MATSFSTLTPFLFILLLSISSVLEAAHHHTAAPAPAVDCSMLILNMADCLSFVSAGGTEAKPASSCCNGLKTVLKTDAECLCEGFKSSASLGVTLNMTKAATLPAACKLHAPSMAACGLSAAPTMAPGLAPGGGAVAAGPDLSFLAPNPSPGNHGSSLLPFSFTTILSTMFFVLFLSRV
BrXYLP3	*Bra013135*	nsLTP2	MAPRTMETSILMIFTVVALMSGERAIAVDCSSLILNMADCLSFVTNGSTVEKPEGTCCSGLKTVVRSGPECICEGFKNSASLGVTLDLAKAASLPSACKVAAPPSARCGLAVSASPPASSPEISPTAGAGAPSSSSEANAATPVPVPAGSSDASLVSVSFAFALFIALISSSFY
BrXYLP4	*Bra037870*	nsLTP2	MKQSLLSILIFLLSSSFAPIHARNKSQPANSPSSVAAPAPGPSNSDCSSVIYDMMDCLSYITPGSNDTKPTKVCCGGILSVLQYNPTCVCVGLESSKTMGFAVNNTRARAMPTTCKLPIVATHCPMLDEVTPAASTPVSQSAGTPMTSPSSVASPTSSPSLAESPVMTAPSPSSSGTNHLSAS

			TLTLVVIKVSFVAYISFFFSN
BrXYLP5	*Bra000652*	nsLTP2	MSPSPVATPAPGPSNSDCSSVIYGMMDCLSYITPGSNDTIPTKVCCGGILSVLQYNPKCVCVGLASSKDMGIEINSTRAHSMPTTCKLPIAAPHCAIPGASAPGASTPVSPSAGTPMTSPSSDESPTSPSSDESPTSSLSLAESPGMTAPSPSSSGTNQLSVSTLTLFAIIVSSITYILGFSN
BrXYLP6	*Bra028639*	nsLTP2	MALFSAALPLLLLFLFVSSLSVNGNTAQSVECSAMIMTLTKCLPFVTIGSQVEKPETACCSVIKTVLDTKAECLCEGLKSSAAAGINLNLTKAGTLPDACQLKAPPMPACALFAKPPASAPAPVPAARPLNGSGPGSNSAPAPSPSHSNHGSSISVLSLAISGALVIMFTRI
BrXYLP7	Bra037988	nsLTP2	MEQSTRSLIITIVITSMLVGFGSSDLDQDREECTNQLVVLSPCLTYVGGNAKAPTKDCCGGFGQVITQSQKCVCILVKDKDDPNLGLKFNASLAAHLPTACHITAPNITKCISLLHLSPNSTLAREFESLGRLEASANSAPPLQNVKDGAGGGKAESVKSDGGKKKKSWLAVELLIFALFSHLLLVISSFTSSSFI
BrXYLP8	*Bra004824*	nsLTP2	MESWRISLVAIAIALLMATLVSAGEDKAKDKEECTEQLVGMATCLPYVQGQAKTPTPDCCSGLKQVLKSDKKCLCVIIQDRNDPDLGLQINVSLALALPSVCHAVADVTKCPALLHMDPKSQEAQVFYQLANGLNKTGPASASTISAPAPTSVSPTAGSEDGNNSGRATSLPSKNHAQSFRKPWLVLEVAAHLFIISFTITL
BrXYLP9	Bra036905	nsLTP2	MSTVSAQSTCTNVLISMAPCLGYITGNSSSPSQQCCSQLAHVLRSSGECLCEVLKGGGPRLGINFNKTQALALPKACNVQTPPVSLCNDDSSVKSPTGPSNTSEHGNGSKTVPGHRSTSRGSSIKVSFSLLAVLSAVSYIINYSRY
BrXYLP10	Bra021454	nsLTP2	MGYTSIYAITFVALVGALLGVSKAQPSGSCVSTLTTLSPCLGYITGNSTTPSQTCCSQLDS

			VIKSSPQCICSAVNSPIPNIGLNINRTQALQLPNACNIQAPPLSQCNVATGPTTPLGALSPVESPADKNPGVALTPTSSPGARSGVVVGARGGSKTIPSTGAGSSSGSVDRVPPHLFIFICEFYYFKEENDLSDLVSCVLRVGIKCCKGCQTNAKRKLLSVSGVSAVEYNAEQGLLRVSGDPNPAKLLRKLAKWDKNAELVSLPGEVSAPAPRTPQLYQTKRMGKRTPKCFLLRCFGTKEKVEPYGVAGDENGSATPFINTVAPPMVYPPPQPTPGFATPIPYPPPCFGANQPPYTYTSGGMYQSPPPTFQLRKTQFPQMVNYPHH
BrXYLP11	*Bra040833*	nsLTP2	MKMGMGLMLLTVFMAVMSSTRVLAQSTCTSALISMSPCLNYITGNTTTPSQQCCSQLGNVVRSSPDCLCQVLNGGGSQLGINVNQTQALALPRACNVQTPPVSRCNNGGGSTADSPADSPNSSGPGNGSKTVPVGEGEGEGPSSDGSSIKFSYPLLAFLSVASYMAIFLKY
BrXYLP12	*Bra023817*	nsLTP2	MKMGRVLVLLTVFMAVMSSTRVSAQSSCTTALISMSPCLNYITGNTTSPSQQCCSQLGNVVRSSPDCLCQALNGGGSQLGINVNQTQALALPRACNVQTPPVSGCSNGGGSTADSPTDSPNSSGPGNGSKTVPVGEGDGSFSDGSSIEISYRLLTFLSVASYIAIFLKY
BrXYLP13	*Bra001874*	nsLTP2	MKTGMGLMFLTIFMAVMSSTRVSAQSSCTSVLISMAPCLNYITGNTTSPSQQCCSQLSSVVQSSPDCLCQALNGGRSQLGLNINQTQALALPRACNVQTPPISRCNGGGSNADSPADSPKSSGPGNGSKTVPVGEGDGSSSDGSSIKFSYPLLAFLIAASYMAVFLKY
BrXYLP14	*Bra018473*	nsLTP2	MAQTTTVIFILATLLVPATVVSGQTPPSPVAPSPTINEAMNCAAGLTVCLPAFAQGGTPSKECCTAVKTQQSCLCGFIKAPVLVVPFNITAFSALISKSCGINTNLNLCSETPAQAPLPHMTAPPSGAPKTDKDAASKPAETGLVGIVLIMISALFY
BrXYLP15	*Bra025919*	nsLTP2	MEVVRFAVAVVFVLFSVSSSKAEPTPAMGGGGGGGGDAHSMPCIQKLMPCQPYLHSVTPPPPASCCLPMKEIVEKDATCLCSVFNNVDMLKSLNLTKENALVLPKACGAKADISLCKSS

			NGTTTPSTGTTTTPPASSTGSGSTGASSSSTDKPTNSAPAINFAGASFASAFMALATIFF
BrXYLP16	*Bra016563*	nsLTP2	MEILKFAVIFVLCSISSSNAATTPPSGGAGDAHSMPCIQKLMPCQPYLRSVTPPPPASCCMPLKEIVETDVNCLCSVFNNVDMLKSLNLTKENALVLPKACGANADVSQCKASTGTTTPSTSPGTTKTPPASPAESGSTGGSASSTAKPSDSAPAINFSGISFAAAFVALATIFF
BrXYLP17	*Bra031024*	nsLTP2	MEIVRFTVAVFFVLYSVSSSNAATAPPSGGGGGGGDAQAMPCIQKLMPCQPFLHSVIPPPPSCCLPLKAIVANDATCLCSVFNNVDMLKSLNLTKDNALDLPKACGANPDISLCKASPAGGTTTNSTSPATPKTPPVSSTGSGSTGASSSTTSPTSSAPAINFAGLSFASTIVALATTFF
BrXYLP18	*Bra031023*	nsLTP2	MKIVRFTVAVFFVLVSVSSSNAAVAPPSGGGSGGGDARALPCIQKLKSCQPYLHSVIPPLPASCCLSMKEMVANDAPCLCSVFNNVDMLKSLNLTRDNALDIPKACDAEPDISLCKASPADGPTTNSTSSTPTSSAPAINFAGLSFASTIVALATTFF
BrXYLP19	*Bra031022*	nsLTP2	MEIVRFAVAAVFFVLYSVSSSNAAIAPPSGGGGGGGDAQEMPCIQKLMPCQPFLHSVIPPPPPSCCLPMKAIVANDATCLCSVFNNVDMLKSLNLTKDNALDLPKACGANPDISLCKASPAGGTTTNSTSPATPKTPPVSSTGDFHCGTTGASASSTSTPTSSAPAINFAGLSFASTIVALAATFF
BrXYLP20	*Bra015955*	nsLTP2	MASSILFITLLISLSSISLQLVFAQVPGTTTATCSSMLLSLAPCGPFVQGFVQLPAQPCCDGLNQIYSQQPTCLCLFLNNTSTLSPAFPINQTLALQLPPLCNVPANASSCSSPGGEAPSDSSSVAPPPSSSTSSPVSPSAKNNSSVAGTPVAQLAPRPTSLMGLGYDLRSSGSKSKIQLIILALAVILPGTLFI
BrXYLP21	*Bra032857*	nsLTP2	MKDLHFHIFLVTMTVIASISAATPTAPAAGGALSDECNQDFQKVTLCLDFATGKAPTPSK

			KCCDAIEDIKEKDPKCLCYVIQQAKTGGQALKDLGVQEAKLIQLPTACQLRNASISNCPK LLGISPSSPDAAVFTSNATTITPEAPAGKSPATPATSSEKGGSASIRDGHAVVALAITLITVSF VSTLL
BrXYLP22	*Bra010912*	nsLTP2	MKGLYFHLFLVTMTVVASISAATPAAPSGGGSLLDECSKDIQTVSLCLDFASGKAPNPSK KCCDAIEDIKEKDPKCLCFVIQQAKSGGQTLKDLGVQEAKLIQLPTSCQLHNASISNCPK LLGISPSSPAAAIFTSNATSTTTPAAPGGTSPATPATSSEKGGSASIKDGHAVMLLAVALMSI SFLSTLPWMGLA
BrXYLP23	*Bra021455*	nsLTP2	MSSLILGGKGQQIISTPCTSSMISTFTPCLNFITGSSGGSVTPTAGCCDSLKSLTSTGMNCA CLILTANVPLPTGFINRTLSLALPRACKMTGVPVQCQAAGTPLPAPGPVPFLLAPPPPMSA FSPGSSKAAATAPGLAPDAPLDGPMGPTATPGIRPVVQPLQPTSLAQYSTSPFLPLLFFLFT LLTLLNL
BrXYLP24	*Bra023816*	nsLTP2	MSKISGITIVLVALIAVLAFPVRSQQPPLSQCTPSMMTTVGPCMSILTNSSTNGTSPSSDCC NSLKSLTTGGMGCLCLIVTGSVPFNIPINRTTAVSLPRACNMPRVPLQCKANIAPAAAPGP ASTFGPAMSPGPATTPLVPEPTPAAQTPQSVTTRPFTPTADGAAPTADNGGSTSRPSLTPSS AYALSPSLLFFGISLVALKFY
BrXYLP25	*Bra001875*	nsLTP2	MSKIPVITIAVALLAVLALPVRSQQPPLSQCTPSMMTTVGPCMSILTNSSTNGTSPSSDCCN SLRSLTTGGMGCLCLIVTGSVPFNIPINRTTAVSLPRACNMPRVPLQCNANIAPAAAPGPA GTFGPAMSPSPATTPIVPEPTPAAQTPQSDTTRPFTPTVDGAAPTSDDGGNTSRPSVTPSSS YALSPSLLFLVVSLVALKFY

BrXYLP26	*Bra015426*	nsLTP2	MDPRFCLISALIFLSLLSNSPILILAQISTPCSPTMLSSVTGCMSFLTGGGSSPTSDCCEALKSLTGTGLDCLCLIVTASVPINIPINRTLAISLPRACGMPGVPVKCKASAAPLPAPGPVSLGPTTPTETQSPQGSASFGPTTSPASSIIPDDQNIPASDKGENPTASTPSASSPSSSHSIKLPLLLLTFFAFQIISLLLS
BrXYLP27	*Bra032462*	nsLTP2	MDPKSFLISALIFSLLSNSPVLMSLAQINTPCSPTMLSSVTGCMSFLTGGATSTTSDCCRALKTLTGTSMDCLCMIVTANVPLDLPINRTLAISLPRACGVPVQCKASSALLYSPGPASVGPTTSPPTETQNPEGSASFGPATSPTSSMDPDGMPDGKAHIFFNT
BrXYLP28	*Bra039713*	nsLTP2	MAATIVFILMLAITSSTAVAETQGPSSSPPAPTCSEELVMFSPCIPYVSAPPNNISKTPDALCCSVFSTSVHSAAGKCLCYLLRQPMILGFPLDRSRLLSLSQICTEFQSSDESFESLCSPSVSPELPPLQSIQFTSPFDYGDRDSASPQSLGLPPETAKDPPISDQFSPDIDNVSPQLIINGSPMISNLLLFLTTIIMTLATCILTRI
BrXYLP29	*Bra025907*	nsLTP2	MASSSVFITVLISLVPVFLQPGLAQGQSPPASCASLLLALAPCGPFVQGFVQFPAQPCCSSLSQIYSQQPTCLCLFLNNNSTLSSAFPINQTLALKLPQLCSIPANSSVCSSGASTASPPSTNSTGSQVSMGAKNNSVAAATPVAQVAPKPSNMMGLGDGLRSSGPTFKIQVTIFVIAAILAGTLFLV
BrXYLP30	*Bra040823*	nsLTP2	MKQSLILSILILLSSSFAQIHARNKSHPANPPSPVATPAPGPSNSDCSSVIFDMMDCLSYLTPGSNDTTPTKVCCGGILSVLQYNPKCVCIGLASSKDMGIALNNTRALAMPTICKLPIAAPHCAILDASRPSASTPGMSSVSPSAVTPMTPQSSAQSPTFSPSLPESPGITAPSPSSSGTNHLSVSKFTFVAVVVSYITYISAFSN

BrXYLP31	*Bra021349*	nsLTP2	MTNVAVIAAILITVLLSASVSEQMAPSPSSGPSGAPDCMTNLLNMTDCLSYVQVGNGGGAANPDKACCPELAGLVDSSPQCLCYLLGGDMAAQYGIKIDKAKALKLPGVCGVVTPDPSLCSLFGIAVGAPEAMGKEEASPAFAPSSGAESPEGLGSGPSASRTSDAPNTPYSLFLSVIIIPLAFAFHLYS
BrXYLP32	*Bra014833*	nsLTP2	MKQSLISILILLLSSSFAPIHARNKPQPAKSPSPVAALAPGPSNSDCSSIIYDMMDCLSYLTPGSNDTKPTKVCCGGILSVLQYNPKCICVGLESSKTMGFAVNNTRARAMPTTCKLTIVAPHCAILDEATPAASIAVTPSAGTPMTSPSSGGSPTSAPSLAESPVMTAPSPSRSGTNHLSVSTLTLVSVIVSSVTYISFLF
BrXYLP33	*Bra037069*	nsLTP2	MAATSNAVVLIVILAITFSSSSAVTETQAPSPPALTCTEELVMFSPCLPYVSAPPNNISDAPDPLCCSAFSTSVNSGAGNCLCYLLRQPMILGFPLDRSRLLSLSQICSDLSSDESFESICSPSESPELPPLQSIQFTAPFVYGDKASASSPSFAISREAAGISPTSDQPSPETDSLSSTPESIINGSPKITSFCFLSTIIMTLPTFDLFLLAL
BrCAGP1	Bra030067	nsLTP2	MKGLHFHLFLVTMTVVVSVSAATPAAPAAGGALADECSQDFQKVTLCLDFATGKAPNPSKKCCDAIEDTKERDPKCLCYVIQQAKTGGQALKDLGAKKINSFNFQLLASSTTLASPTVQEMHVVDYGFEFLGISPSSPDAAVFTSNATSTTTPAAPAGKSPATPTTSMGTGGSPSIRDGHATVALAFALIMTVSFVSILPRMGLA
BrCAGP2	*Bra039574*	nsLTP2	MKHLVVLSNVLLLLSCDAAVFMSPSESPVFSPSSEPSNNDCSTVVYGMFDCLSFLTVGSTDLSPTKTCCEGIKIVLEYNSSCLCIALESSRAIGFDLINNRALAMPSTCNIPIDPHFVSPSKPPTTTLSSGSSTSITTTSPSVSSPASSHSSAAKPGSSPTIIQSPPTLAAQSPAMFAPSPSEYGMENMSLSKLFIIIMMISSFVYLLA

BrCAGP3	*Bra021876*	Pollen proteins Ole e I like (POeI-like) domain	MGFIGKSVLLTLIALCCFTSSVFSTIAQVPPVKLITPLPTLPPTKAPIKVPTFPPAKAPIKVPTLPPAKAPIKPPVVLPPVSPPKFNRTLVAVRGVVFCKACKYAGVNNLQGAKPVKGAVVRLLCKNKKNATSEATTDKNGYFLLYAPKTVSNYAIRNCRAYLVKSPDAKCSKVSKLHGGYLGSFLKPVVKPENATIIFNKLKYSLFNVGPFAFEPVCPK
BrCAGP4	*Bra032889*	POeI-like	MGFLGKSILSSLLAIWFFTSCAFTEEVNHVNQTPSSAPAPSPYHHGHHHPHPPHHHHPHPHPPAKAPVKPPVSPPSKPPVKPPVYPPTKSPVKPPTKPPVKPPVSPPAKPPVKPPVYPPTKAPVKPPVKPPVKPPVSPPAKPPIKPPVSPPAKPPVKPPVYPPTKAPVKPPTKAPAKPPVSPPAKPPVSPPAKPPVKPPVYPPKFNRSLIAVQGTVFCKSCQYASFDSLIGAKPVEGAVVRLLCKSKKNIVAETKTDKNGYFLLLGPKTVTNYGFRGCRVYLVKSKDYKCNKVSKLFGGDVGAVLKPEKRKGKSAVVINQLIYGIFNVGPFAFDPVCPK
BrCAGP5	*Bra030083*	POeI-like	MGFLGKSVLVSLIALWCFTSSAFTEEVNHVTQTPSSAPAPAPYHHGHHHPHPPHPHHPHPHPPAKAPVKPPVSPPAKPPVKPPVYPPAKAPVKPPTKPPVKPPVSPPAKPPVKPPVYPPTKAPVKPPVKPPVKPPVSPPAKPPVKPPVYPPTKAPTKPPTKPPVKPPVSPPAKPPVKPPVYPPKFNRSLVAVQGTVFCKSCKYASYDSLTGAKPVEGAKVRLVCKSKKNIVAETETDKNGYFLLLAPKTVTNFGFRGCRAYLVKSKDYKCNKVSKLFGGDVGAVLKPVKTPGKSSVVINKLTYGVFNVGPFAFDPVCPK
BrCAGP6	*Bra003506*	POeI-like	MAVTRAALAICFLLSLATIATADYYSPSTPPVYTPPAYKPTHPPPVYTRPVHKPTLPPPVYTTPAHKPTLPPPVYTPPTYKPTLPPPVYTPPTYKPKPTLPPPVYKPTLSPPVYTKPTIPPPVYTPPVYKPTLSPPVYTKPTLPPPVYTPPAYKPTLPPPVYTPPVYKPTLSPPVYKPTLSPPVYKKYTSYSHTTPYVPKPTYTPPTKPYVPEILKVVDGIILCKNGYETYPIQGAKAKIVCSEPGSYGQSKKDVVIYSDPTDSKGYFHVSLTSIKDLLHCRVKLYTSPVETCNNPTNVNKGLTGVP

			LSMYGYRYHSDKNLKIFSVGPFYFTGHKAAPTTPKY
BrCAGP7	Bra014398	POeI-like	MLSTMAIKSTSLAICLFFSPKPYVSKPTYTPPTKPYVSKPTYTPPTKPYVSKPTYTPPTKPYVSKPTYTPPTKPYVSKPTYTPPTKPYVSKPTYTPPTKPYVSKPTYTPPTKPYVSKPTYTPPTKPYVSKPTYTPPTKPYVSKPTYTPPTKPYVSKPTYTPPTKPYVSKPTYTPPTKPYVPEILKVVDGIILCKNGYETYPIQGAKAMIVCSEPGSYGKKDVVIYSDPTGSKGYFHVALTDIIKNLLHCRVKLYTSPVETCKNPTNVNKGLTGVPLSMYGHRYHSDKNLKIFSVGPFYFTGPKAAPTTPKY
BrCAGP8	Bra014399	POeI-like	MAIKSTSLAICLLFSLATIATAYYSPSSPPVHQSPEYKPTLPPPVYIPKPTLPPPVYTPPVYKPTLPPPVYTKPTLPPPVYTPPTKPYVPKPTYTPPTKSYVPKPTYTPPTKPYVSKPTYTPPTKPYVSKPTYTPPTKPYVSKPTYTPPTKPYVSKPTYTPPTKPYVSKPTYTPPTKPYVSKPTYTPPTKPYVSKPTYTPPTKPYVSKPTYTPPTKPYVSKPTYTPPTKPYVSKPTYTPPTKPYVSKPTYTPPTKPYVSKPTYTPPTKPYVSKPTYTPPTKPYVPEILKVVDGIILCKNGYETYPIQGAKAKIVCSEPGSYGKKDVVIYSDPTDSKGYFHVALTDIIKNLLHCRVKLYTSPVETCKNPTNVNKGLTGVPLSMYGYRYHSDKNLKIFSVGPFYFTGPKAAPTIPKY
BrCAGP9	*Bra001983*	POeI-like	MDPNHNLLFVSFLVLCLAVNGVTGYATVTGSVFCDQCKDGERSLFDFPVSGIKVSVTCADESGQVYMSREETTNWLGGYVMRFDGTPDLSNCYAQVSDNGAQQQGSSSSCSIASGPAQKLKLLFSFFGFETFAADVLLTQPVRPMSYCPKPPPAPVMSPPHQAPPPPEVKLPPSPPKPQVPVLPPPQAPVTSPPPATSPPQFKLPPLPPIPPVPFVDPSACSHQLWIRPEYRCYWRVIGPDTKVAVAFGLIAGRRYGTDMTVREALDGRGEAYKTLLREATTALLNSYNSLGFPYNSIAVITHTNLALLGNSQHDVLMTAIRFIKANSGTCKFTVCK

BrCAGP10	Bra033328	glycosyl hydrolase domain (GH)	MLVSSYTRNHILFFIALILTLTSLTESRYHHHKEKHKHNSHNHHSSKPEPPSSSISQPPTPPPGPNDSPSPSLPPSPSDEPEEDNNGFYNVRKFGAVGDGVTDDTEAFKTAWDSSCSNQNDTVSVLFVPYGYTFMIHSTIFTGPCHSYQILQVDGTIITPDGPESWPSNISKRQWLVFYRVNGMALKGAGVIDGRGQKWWDLPCKPHRSVNKSAIFAGPCDSPIALRFFMSSNLTVEGLMIKNSPQFNFRFDGCQGVHVESLHITAPPLSPNTDGIHIENSNSVTIYNSVISNGDDCVSIGSGSYDVDIRNLTCGPGGHGISIGSLGNHNSHACVSNITVRDSIIKYSDNGVRIKTWQGGSGSVSGVTFNNIHVESVRNPIIIDQYYCMTKDCSNKTSAVFVSDIAYQGIKGTYDIRSPPMHFGCSDAIPCTNLTLSGIELLPAKGDIVLDPFCWNAYGLAEELSIPPVWCPMSDPPTALPGALVDKCGSP
BrCAGP11	Bra032600	GH	MLISSYTRNQILCFIALIITLSSLTESRYHHHKEKHKHNSHNHHSSKPEPPSSSISQPPTPPPGPDDSPSPSLPPSPSDDPEEDDNGVYDVRKFGAVGDGVADDTEAFKTAWDSSCSNGNDTVSVLLVPYGYTFMIQSTIFTGPCHSYQLFQVDGTIVTPDGPESWPSNISKRQWLVFYRVNGMALKGAGVIDGRGQKWWDLPCKPHRTVNISAIVAGPCDSPIALRFFMSSNLTVEGLQIKNSPQFHFRFDGCQGVHVESLHITAPPLSPNTDGIHIENSNSVTIYNSVISNGDDCVSIGSGSYDVDIRNLTCGPGGHGISIGSLGNHNSHACVSNITVRDSVIKYSDNGVRIKTWQGGFGSVSGVTFNNIHVESVRNPIIIDQYYCMTKDCANKTSAVFVSDITYQGIKGTYDIRSPPMHFGCSDAVPCTNLTLSGIELLPEKGEIVVDPFCWNAYGIVEELSIPPVWCLMSDPPTALQGALVDKCGSP
BrCAGP12	Bra011100	GH and X8	MSERLKLILWICLSILAFLDFGGAASKIGICYGRNADNLPTPNKVSELIQHLNIKFVRIYDANIDVLKAFANTGIELMIGVPNADLLAFAQFQSNVDTWLHNNILPYYPTTKITSISVGLEVTEAPDNATGLLLPAMRNIHTALKKSGLDKKIKISSSHSLAVLSRSFPPSSATFSKKHLPFLKP

			MLEFLVENESPFMIDLYPYYAYRDSSEKLDAIYFALTAMNFKTVKVMVTESGWPSRGSP<br>KETAATPDNALAYNTNLIRHVVGDPGTPAKPGEEIDVYLFSLFNENRKPGIESERNWGMF<br>YANGTSVYALDFTGESSVPVSPSNSSTTSPGPSSSPGNSTVIIGGGGGGARKWCVASSQAS<br>VTELQTALDWACGPGSVDCSAVQPDQPCFEPDTVLSHASYAFNTYYQQSGANSSDCSFG<br>GVSVEVDKDPSYGNCLYMIAPSTDGMNRTMAGNITGNITAIDSPMASPSSSSDGYRQMV<br>VSVAVSVLLPLFVVSLSLW
BrCAGP13	Bra010330	GH and X8	MSLFKTVAELGAASNIGICYGRNADNLPSPNKVSELIQHLNIKFVRIYDYNIDVLKAFANT<br>NIELMIGVPNADLLAFAQFQSNVDTWLRNNILPYYPTTKITSISVGLEVTEAPDNATGLVL<br>PAMQNIHTALKKAGLDKKIKISSSHSLAILSRSFPPSSATFSKKHSAFLKPMLEFLVENDSP<br>FMIDLYPYYAYRDSAEKVQLEYALFESSSQVVDPATGLLYSNMFDAQLDAVYFALTAMN<br>FKSVKVMVTESGWPSKGSPKETAATPDNALAYNTNLIRHVIGDPGTPAKPGEEIDVYLFS<br>LFNENRKPGMESERNWGMFYANGTSVYALDFTGESAVPGPVSPSNSTTGVSPSPGDNGN<br>STVTIGGGGGGAKKWCVASSQASVTELQSALDWACGPGNVDCSAVQPNQPCFEPDTVL<br>SHASYAFNTYYQQSGGSSLDCSFGGVSVEVDKDPSYGNCLYMIAPSTDGMNRTMAGNI<br>TGNITAIDSPLASPSTSNEGIRQMVVSVAVSALLPCFVVSLSLLW
BrCAGP14	*Bra000732*	GH and carbohydrate binding domain (CBM49)	MHPCKVLISWDVKYAGVQTLVAKILMQGKAGEHTAVFERYQEKAEQFMCSMLGKSTK<br>NIQKTPGGLIFRQRWNNMQFVTSASFLAAVYSDYLSSSKRNLRCSQGNVSPSQLLDFSKS<br>QVDYILGDNPRGTSYMVGYGHNYPRQVHHRGSSIVSYKVDQKFVTCRGGYATWYSRK<br>ASDPNVLTGALVGGPDAYDNFADNRDNYEQTEPTTYNNAPLLGVLARLISGPTGFDQRL<br>PGVSPTPSPVIIKPAPIPKRKPTTPPAPASSPSPITISQKMTSSWINEGKVYYRYSTKLTNRST<br>KRLKNLKISITKLYGPIWGVTKTGNSYGFPSWMKSLPAGKSMEFVYIHSAAPANVLVSN

			YSLE
BrCAGP15	*Bra001454*	GH and STAS domain	MKTYLLLLLIFSLLLSYSSGQQCGRQAQGALCPNGLCCSEYGWCGTTEAYCGRGCQSQ<br>CTPTPPTPTPTPPSPTPPRPTPPGPSGDLSGIISRDQFYKMLKHMNDNACPARGFFTYDAFI<br>TAAKFFPSFGNTGDLATRKKEIAAFFGQTSHETTGGWTDAPDGANTWGYCFKDEIGKSN<br>PYCDSNNLEWPCAPGQFYYGRGPMMLSWNYNYGQCGRDLGLDLLRRPAIASSDPVIAF<br>ETAIWFWMTPQAPKPSCHDVITDQWQPSAADISAGRLPGYGVITNIINGGLECAGRNVA<br>QVEDRISFYTRYCGMFGVDPGTVLGRLPGTTVYRNMKQYPEAYTYNGIVIVRVDAPIYF<br>ANISYIKDSCHIHRLKRRGSLERTLAVSNPNKEVLLTLARSGIVELIGKEWYFVRVHDAVQ<br>VCLHYVESKNQTPTNVEESSSSSLWRRCNAKNSSHTEVEPDSKLVLKEPLLFNDK
BrCAGP16	Bra038726	GH	MKSLLLLLLNFLFLLSFSSAEQCGRQAGGALCPNNLCCSEYGWCGSTEAYCALPGCQSQ<br>CTPSGPPPPGPPPPDPTGGLTDIITRSQFDDMLKHRNDAACPARGFYTYDAFITAAKYFPS<br>FCNNGDTVARKKELSAFFGQTSHETTGGWPTAPDGPYAWGYCFKEEVSPSSDYCQPSGQ<br>WPCVPGKRYYGRGPMQLSWNYNYGQCGAAIGEDLLNNPDLVSNDPVISLKAAIWFWM<br>TPQSPKPSCHAVINGQWQPSPADIAAGRVPGYGVTTNIINGGLECGHGPDTRVYDRIGFY<br>QRYCGIFGVNTGDNLDCYNQRSFASFKSFLDAAM
BrCAGP17	Bra034754	GH	MKTCLLLFLIFSLLLSFSSAEQCGRQAGGALCPNGLCCSEFGWCGNTEPYCKQPGCQSQ<br>CGGTPPGPTGDLSGIISRSQFDDMLKHRNDNACPARGFYTYDAFINAAKSFPGFGTTGDT<br>ATRKKEIAAFFGQTSHETTGGWATAPDGPYSWGYCFKQEQNPSSNYCSPSAEWPCASGK<br>SYYGRGPMQLSWNYNYGQCGRAIGSDLLNNPDLVSNDPVIAFKAAIWFWMTPQSPKPS<br>CHAVIVGQWQPSDADRAAGRVPGYGVITNIINGGLECGRGQDATRVADRIGFYQRYCNI

			LGVNPGGNLDCYNQRSFASVNFFLDAAI
BrCAGP18	*Bra033641*	GH	MEPSVYRLFLLFFLCLYGLHQTKSQPFIGVNYGQTADNLPPPSATAKLLQSTSIQKVRLYG SDPAIIKALANTGIEIVIGTGDVPGLASDPSFARSWVETNVVPYYPASKITLIDVGNEATTF GDRNFMLQLLPAMKNVQSALEAASLGGKIKVSTVHTMSILSQSDPPSAGVFAADHADIL KGLLEFNRETGSPFAVNPYPFFAYQSDPRPETLAFCLFQPNPGRVDANSKIKYMNMFDAQ VDAVYSALSSFGFKDVEIVVAETGWPYKGDPDEVGTTIENAKAYNKNLIAHLKSMAGTP LMPGKVIETYLFALYDENLKPGKGSERAFGLFKPDLTMTYDIGLTKTTNQTSMAPQSPTP RLPPAAAPTSQTLPAPPQMILPSPTSPSDKNSGQTDVHNSTPR
BrCAGP19	*Bra027699*	GH and CBM49	MEIKFASVAALLLLLSFPVAFSGHDYGQALSKSLLFFEAQRSGVLPRNQRVTWRSHSGLT DGKSSGVNLVGGYYDAGDNVKFGLPMAFTVTMMAWSVIEYGNQLAANGELGHSIDAI KWGTDYFIKAHPEPNVLYGEVGDGNTDHYCWQRPEEMTTDRKAYRIDPSNPGSDLAGE TAAAMAAASIVFRRSNPAYSRLLLTHAYQLFDFADKYRGKYDSSITVARKYYGSVSGYN DELLWAAAWLYQASNNQFYLDYLGRNGDSMGGTGWSMTEFGWDVKYAGVQTLVAKF LMQGKAGRHTAVFQKFQQKADFFMCSLLGKGSRNIQKTPGGLIFRQRWNNMQFVTSAS FLTTVYSDYLTSSRSNLRCSAGNVAPSQLLSFAKSQVDYILGDNPRATSYMVGYGNNFPQ RVHHRGSSIVSYKVDRSFVTCRGGYATWFSRKGSDPNLLTGAIVGGPDAYDNFADRRDN YKQTEPATYNNAPLLGVLARLSSGHSGYSQLLPVVPAPVVVRRPMPIRKPRVTSPVRASG PVAIVQKMTGSWVSKGRTYYRYSTTVINKSPRALKSLNLSIKNLYGPIWGLSRSGNSFGL PSWMQSLQSGKSLEFVYIHSTSPANVAVSSYTLA
BrCAGP20	Bra027735	GH and	MKNFASVAALLLLLCFPVAFSGHDYGQALSKSLLFFEAQRSGVLPRNQRVTWRSHSGLT DGKSSGVNLVGGYYDAGDNVKFGLPMAFTVTMMAWSVIEYGNQLAANGELGHSIDAI

		CBM49	KWGTDYFIKAHPEPNVLYGEVGDGNTDHYCWQRPEEMTTDRKAYRIDPSNPGSDLAGE<br>TAAAMAAASIVFRRSNPAYSRLLLTHAYQLFDFADKYRGKYDSSITVARKYYGSVSGYN<br>DELLWAAAWLYQASNNQFYLDYLGRNGDSMGGTGWSMTEFGWDVKYAGVQTLVAKF<br>LMQGKGGRHTAVFQKFQQKADFFMCSLLGKGSRNIQKTPGGLIFRQRWNNMQFVTSAS<br>FLTTVYSDYLTSSRSNLRCSAGNVAPSQLLSFAKSQVDYILGDNPRATSYMVGYGNNFPQ<br>RVHHRGSSIVSYKVDRSFVTCRGGYATWFSRKGSDPNLLTGAIVGGPDAYDNFADRRDN<br>YEQTEPATYNNAPLLGVLARLSSGHSGYSQLLPAVPAPVVVRRPMPIRKPRVTSPVRASG<br>PVAIVQKMSGSWVSKGRTYYRYSTTVINKSPRALKSLNLSIKNLYGPIWGLSRSGNSFGL<br>PSWMHSLQSGKSLEFVYIHSTTPANVAVSSYTLA
BrCAGP21	*Bra018152*	Plant invertase/pectin methylesterase inhibitor (PMEI) domain and Pectinesterase	MLRGNGIFHICLLASFLLLPFSSAVHDGDFSGGANAPSSWDHNIAPSQETAPSPTPTTSPP<br>TTSPPSPGPAAAPSPINNDSISGDMTWWCNKTPHAETCNYYFQRSPHNNINRPPRFRSEFL<br>RLLVQVALDQAVITHAQTVKFGPSCTNNQRKAAWSDCVKLFENTVAQLNLTLKGLNPA<br>ASSDVKCSNFDAQTWLSTAQTNIETCRSGSEDLKVSDFVMPAISNKNLSDLIGNCLAVNG<br>VLMKQHNHTTANHKEYFPSWVSRHERRLLVSASLAKSRPHLVVSQDRSGHFRSIQSAIN<br>FAGRRRIKSRFIIYVKKGVYRENIEVGNDNHNIMLVGDGARKTIITSGRSVKNGYTTYNS<br>ATAGFGGQRFVAKDMTFINTAGPLRGQAVSVRSSSDLSVFYRVGIHGFQDTLYIHSQRQF<br>FRECYISGTIDFIFGNAAVVFQNCMILVRRPLRGQANVITAQGRGDPFQNTGITIHSSRIIA<br>ASDLRPVIGAYKTYLGRPWQAYSRVTIMKTYIDNSISPLGWSPWLRGSNFALNTVFYGE<br>YKNFGPGSSTRWRVRWKGFHAITSAAVASRFTVGSLIAGGSWLPSTGVPFKTGL
BrCAGP22	*Bra033792*	PMEI and Pectinesterase	MVRGIFHICLLASFLLLPQFSSTVNYRGFTVGANVPSPWDHNIPPPPETTPFASPTTSPPTT<br>SAQSPGPAAASSPINNSSISGDMTWWCNKTPHAKTCTYYFQKSPDRNISRPPRFRSEFLR

			MLVHVALDQAVIAHAQTVKLGQSCTNNQQKGAWSDCVTLFENTVTQLNQTFNGLNPGASSDVKCSDFDAQTWLSTAQTNIQTCRSGAEDLKVSNFVMPAISNKNLSDLIGNCLAVNGVLMKQHNHKTANHKEYFPSWVSRHERRLLVSASLAKSRPHLVVAQDRSGHFRSIQAAINFAGRRRIKSRFVIYVKKGVYRENIEVGNDNHNIMLVGDGERKTIITSARSVKGGYTTYNSATAGFGGQRFVAKDMTFINTAGPLRGQAVSVRSSSDLSVFYRVGIHGFQDTLFIHSQRQFFRECYISGTIDFIFGNAAVVFQNCMILVRRPLHGQANVITAQGRGDPFQNTGITIHSSRIVAASDLRPVIRAYKTYLGRPWQAYSRVTIMKTYIDNSISPLGWSPWLRGSNFALNTVFYGEYKNFGPGSSTRWRVRWKGFHAITNAAVASRFTVGSLIAGGSWLPSTGVPFKTGL
BrCAGP23	*Bra034140*	PMEI and Pectinesterase	MKTLHFSSSLLFLSVLFLSCAFLISPQAPSPSPSELSSQPPSLPPSQSPSLPPSQPPSLPPSKPPSLPPSQSTSDACKSTPYPKLCRTILSAVKSSPSDPYSYGKFTIKQCLKQASRLSKVINGYVRRVRSKPGSMTAEEIGAVADCGELAELSVSYLETVAAELKMADMMTAALVEHVNSLLSGVVTNQQTCLDGLVEAKSGFAAAIGSPMGNLTRLYSVSLGLVSHALNRNLKRFKASKGKILGGRNSTYREPLETLIKVLRKTCDNDKDCRKAARKLGELGVTSGGSILVSQAVIVGPYKSDNFTTITDAIAAAPNNARPEDGYFVIYAREGVYEEYIVVPINKKNLLLMGDGINKTIITGNHNVVDGWTTYNCSSFAVTGERFMAVDVTFRNTAGPEKHQAVALRNNAEGSTFYRCSFEGYQDTLYVHSLRQFYRECDIYGTIDFIFGNAAAIFQNCNIYARKPMAKQKNAITAHGRTEPNQNTGISIINCTIKAAPDLAADPTSTMTFLGRPWKPYSRTVFMQSYISDIVQPVGWLEWNGTTGLDTIYYGEYDNFGPGAKTDRRVQWLGYNLLDMAQAMNFTVYNFTLGDTWLPQTDIPFYGGLVRKE
BrCAGP24	*Bra040283*	PMEI	MESNKTFWIIILIVTVSTFLTPMVESKSVPLRDSYVARSLLSVSPPSESPSSSPAPGPEVENTIAVPASSPTEIDIDSPSPSPGAPADSISPTNAPTTSSPSPSPEAPANAPATDSPSPSPEVDMDSP

			SPSSEAPVDSTSPANPPTEMDIISPSPSPEAPEDSASPANPPTEMDIDSPSPSPETPEEIPGAPSGKTLISSATTLLKQTLLSPEIKTICGKTDNPELCESSISPLLTAAIKPDASSVLVLAIQASINATKAVMPTVNKVAAADCQELYDDAVSNLEDAINAVNESDIATVNSNLSAAMTDYGTCNDGFEESGEPNPLADVADKLHKMVSNCLAISTLIK
BrCAGP25	*Bra024570*	PMEI	MKKPSLHQPILFFFLATLLPLILTVHSQPSPSPSPSPSPSPSPSDDSDFIQRSCNTTLYPDVCVSSLSNFSTYVHNDPALLARAAISVTLSNALELGKYLSNVSTLLEIHEDGGHHPTAAAVFHDCFENLKDAVDEMRDSMKQMRDLVTTGSLESFRFQMSNVQTWLSAALTNEETCTDEFKDVQDEPRKDEVCARVDGVKKLTSNALALVNRCVDNAIH
BrCAGP26	*Bra033660*	Xylanase inhibitor N-terminal (TAXi-N) and TAXi C-terminal (TAXi-C)	MDRASLLALLLLLLIIFDLTAADKIPDLAAESGMIFPLSYSSLPPRVEDLRLRRRILHQSQQLPNAHMKLYDDLLANGYYTTRLLIGTPPQEFALIVDTGSTVTYVPCSTCKHCGKHQDPKFQPELSTTYEAVKCNPDCNCDDDGKLCIYERRYAEMSSSSGILSEDLISFGNESQLSPQRAVFGCENVETGDLFSQRADGIMGLGRGKLSIVDQLVDKGVIEDSFSLCYGGMEVGGGAMVLGKISPPAGMVFARSDPFRSPYYNIDLKQMHVAGKSLKLNPKVFNGKHGTVLDSGTTYAYFPKEAFNAIKDAVIKEIPSLKRIHGPDPNYDDICFSGAGRDVAEIHNFFPEIAMEFGNHQKLILSPENYLFRHTKVRGAYCLGIFPDRDSTTLLGGIVVRNTLVTYDRENDKLGFLKTNCSDLWRRLASPPDSPAPTSPVTQNKSLNNISPSRSPSPAPSKAPTVDLPGVFRIGVITFQVSISVNNASMKPNFSEIADFIAHELEIQSSQASLNMHFPLVRLLSITTSGNEYRLKWGIYPPQSSEYISNNTALNIMSLLKENKLRLPGQFGSYKLLEWKAEQKRKQSWLEKHLLGVVVGVMISLLVTSVIVKLALVWRRRQQEEATYEPVSAAVKEQELQPLSSSETSNA
BrCAGP27	Bra036814	TAXi-N and	MLQFGFCVMSLGCASVSGSFSFQIHHRFSDQVKTVLGSHGLPEMGTLEYYETLVHRDRGRRLTSNNNQTTVSFAQGNSTQEISFLHYANVTVGTPAQWFLVALDTGSDLFWLPCNCKSS

		TAXi-C	CIRSMETDQGERIKLNIYDPTISTSSSKVPCNSTLCALRNRCVSPLSDCPYRIRYLSPGSRSTGVLVDDVIHMRTEEGEPRDARITFGCSESQVGLFEKTAVNGIMGLAIANIAVPNMLAKAGVASNSFSMCFGLKGKGTISFGDKGSSDQLETPLSGTLSPPFYDVTITEFKVGSVTVETEFTAIFDSGTAVTWLIEPYYTAVTTNYHLQVADRRLPARVKSPFEFCYIITSATDEEKIPSISFEMQGGATYNVFSPVLVFDTSDGGQVYCLAVLKEVTAGFNIIGQNFMTNYRIVHDRERMILGWKESDCNDKNGFTGPTASANPPSLPPTPSPRARSPSTRLNPLAASSLLILCFFSFICL
BrCAGP28	Bra036815	TAXi-N and TAXi-C	MAIGRHVFVLLSALVLSWGLERCEATGKFSFEVHHMFSDAVKQNLGFDNLVPEEGSLEYFKVLAQRDLFRGRGLASNNEDSPLTSAEGNLTVFVSFLGSLHYANVSVGTPATWFLVALDTGSDLFWLPCNCGVTCISDLKDAGFPQSVPLNLYSPNTSSTSSSIRCSDDRCFESSRCSSPSSSVCPYQVSESTTTTSTGTLLQDVFHLVTEDVDLKPVEANVTLGCGQRQTGLFQNFQAVNGVLGLGVKDYSVPSLLAKAKLAANSFSICFGRVIGVVGRISFGDKGYTDQSETPFISVEPSTAYGVNVTGLSVGKKAVGFSMFAQFDTGSSYTHLREPAYSAFTKAFNSRALDIRTPSDPQFPFEFCYNLSPNATNITFPPIDMTFEGGSVMSIKNPFVTIKTEGGGRMYCLSIVKIEDLSPNIIGQNLMAGYRIVFDRERMVLGWKRSNCFEDESLTSAPPAEFGDTPPPPSESEGPSPPESEAPSPPTESNPLLRSPPPPPLVFTTTPPSDSTESPGSSGVANVSPLGSLLLLSLLAFL
BrCAGP29	*Bra012857*	TAXi-C	MAVARHVFVFLSVLVVVSWGLERCEATGKFSFEVHHMFSDAVKQTLGLDNLVPEKGSMEYFKVLAHRDQLIRGRGLASNNEKPSVTFMRENLTIGVDVLGSLHYANVSVGTPATWFFVALDTGSDLFWLPCNCGTTCIRDLKDIGLPQNQTGLFGEGIALNGLLGLGLEDYSVPSVLAKANITANSFSMCIGNVIDVIGRISFGDKGYTDQQETPLVPVGPSPTYAVDVTEVSVGGEALGIKLLALVDTGTSFTHLLEAEYDLVTKTFDDQVKDKRRPIDPKIPFEFCYDLSPNSTTIYFPKIIMTFGGGSQMILRNPLFSVFNEDGTAMYCLGILKSVNFKLNIFGQNFLSGYRIVFD

			RERMVLGWKRSNCYEDESLEATPPPPEIEAPSPRLSAPLPSPPPPPLVSIATPPPFDPRSSTG NGSGGAASLSPLSSQLLLLLSLLAFLSFT
BrCAGP30	Bra013345	Leucine rich repeat (LRR)	MKLPLPLLLLLLLLSPTTSAAPSLSPTPSPTISPVPRTSPTPPRTSSSSSPLDPKQLKALESLN IPTLRNPCDHHPSSSSKPPTTVVTCDTGSPFRLVTSLSFTNCSSDLSISSTALKALSPSLPSLS FHNCPSLSPPPHLPDSLHSFSAVSSFPRLSGLSLARLVNLTDITVSSVPVSTSGLFVILGNM HDIVSLTISHANLSGNIPKSFHSNLTFIDLSDNLIKGPIPTSITLLSNLKALNLSSNSISGEIPD SIGDLISLKNLSLSSNKLSGPIPDSISSIPDLTHLDLSGNQLNGTVPRFISKMKSLKYLNLAN NAFHGVLPFNASFLKKLEVFKVGGNSDLCYNHTVLSSKMKLGIAPCDKHGLPLSPPPRK EDSSSDDDYSEDDSSEKKKEEHHGPNKAVLGVAIGLSSLVFLIIFMILLAKWCG
BrCAGP31	*Bra020993*	LRR	MKPLSPSLPLLLLLLLSSTTFAAPSLSPTPSPTISPIPRTSPRTSSSPLDPKPLKALESLNIPTA KNPCDHRPTSKPPSTVVTCDAGSPFRLVTSLSFTNCSSDLSISSAALRALSPSLASLSFLNC PSLSPPPRLPTSLRSFTATSSFLRRRKGLSGVYLARLVNLTDLTVSSVPVSTSGLFVILGNM DKIVSLTVSHANLSGNIPKSLHSNLTFIDLSDNLIKGSIPTSITQLSNLKSLNLSSNSISGEIP DSIGDLISLKNMSLSSNKLSGPIPDSISSLPDLTHLDLSGNQLNGTVPRFITKMKSLKHLNL ANNNFRGVLPFNASFLKKLEVFKVGGNSDLCYNRTVLSSKMKLGIAQCDKHGLPLSPPP QKEDSSSDYDYGSEDETSVKKKEESRGPNKVVLGVSIGLASLVFLIIFLILCAKWCG
BrCAGP32	Bra026260	LRR	MALSITFHALTSLLLFHVSLVSTATHTPHHHTSHSSNHLQKAYRALQAWKKVIYSDPKNL TGDWIGPSVCSYTGIFCAPSPSDQNTLVVAGIDLNHGDIAGFLPEPIGLLSDLALIHLNSNR FCGILPRSLSQLSLLYELDLSNNRFVGPFPDVVLSLPSLKYLDLRYNEFEGPLPPKLFSNPL DAIFVNNNRLTSLIPRDFTGTTASVVVFANNDFSGCLPPTIAHFADTLEELLLINSSLSGCL PPEVGYLYKLRVLDMSYNSLVGPVPYSLAGLGHLELLNLDHNMFTGAVPLGVCVLPSLQ

			NLTVSDNYFSEEEGTCRNLTSRGIVFDDSNNCLPDKPHQRSHKVCEDVLDHPVDCYDHE CSAVAPQASPFAAGPSLAPASAPAYT
BrCAGP33	*Bra039561*	Formin Homology 2 domain (FH2)	MGRLRTAFLAVSLVFLVCVSEEIISRRGANRGEAHGGDDVAEQTWIHCRKELKDKNRDC LIYIPPRVAAANDTYQKLSVLTGWFSNWFGPLLDSTTSYPTRKLIGKQKRKKKKRKKFR VSAPNFALGPAPGFAPGPSPRFAPGPAPTTPQSYDLVAPSSSPSYSPAEAPDESSFGGPTKK RAKSIVAPSQSVPGPPPPPPPEKKNDILMDLIIAVASTAVLTFFLVALLFLCCFRRNNRKNAV GPRNGPRDEGPLLHLSDLSAGSNENSPKVAATSRRFFTATSKKRSFLSRVSLKRNHHDFPP AEASSSSGLPLPPGRSSGAPPLPPVAPPPPQAPPPPPPKSKPPPPAPPKLVRPPPAPPKRQGR SSSGDGSDVDSETGAPKTKLKPFFWDKMANPDQKMVWHEISAGSFQFNEQEMESLFGY NDVNKNKNGQRGESSRDSPVQYIQIIDPRKAQNLSILLRALNVTIEEVVDAIKEGNELPV ELLQTLLKMAPTSEEELKLRLYSGDLHLLGPAERFLKILVDIPFAFKRIESLIFMISLQEEVS GIKESLATLEVACKKLKNSRLFIKLLEAVLKTGNRMNVGTFRGDAQAFKLDTLLKLSDV KGTDGKTTLLHFVVLEIIRSEGVRALRLQRSSKSFSSVKTDDTNTDTSPQSVERYRSTGL QVVSGLTTELEDVKRAAIIDADGLASTLMNLSGSLTNAREFLKTMDEESDFEKALTGFIE RADGDIKWLKEEEERIMVLVKSSADYFHGKSAKNEGLRLFAIVRDFLIMLEKVCREVKE TTMMTSSKNHSSKKETKMIPESNQPDNIRRHLFPAIAERRADSSDDSDSD
BrCAGP34	*Bra037491*	FH2	MQTFCFSFLLLLSCAPSPLSYASSALNLGRRHLSDDAGSALLTPASPTSPPFFPLESSAPPLP PPPSPPTFAAFPTTFPANISALVLPRSPKPHSTSPAILVPAISAVLVVAAVLGVALFLYGRWK GQNRHFKDTNTTLGGGSSSSSHTSEERHVITNNFSVSASSPTSEVLYLGGEEEPDRVVTSF VKPPESPEIRPLPPLPRSFQPSYEAEIHSERNEEEEDEEEEEEFFSPLASLASSANSSPSRSGF EQSSCSSSSSGWVSPARSFSMTLSPVQQQRSFSRLSDVSLEQSLQSLSPERLRVRNNNGH

			VSSSLRMFSFFNQNLSFPRISSASTSPDRGGFVRTPLSSLYSSVSNSPDGLFRKFINSSPPIW NDFSRNVKSVLLSSESVSSRRDFVINIGGQAGAVAAPPPTRPPPLVPPSQSFVVQNDVKKP SFSDKVNQGSCQNTAWDCLKANSFKLNKEIVETLFISNSSSPNLNQRGLTYDLPTQNEVS YQNIATRLQLLNLTTKDVSNALLEGDSGALGAELLDCLSRLAPSKEEERKLRNASDDSVI IKLTPAERFLKELLQVPFVFKRVDALLSVANFYPEVEYLRRSFGVVQAACEELRNNKTFS RLLEAILKTGNKMSVGTNSHAFKFDTLLKLAEVKGLDGRSSVLHFAVQEMIKSEGSVKA LDRIRNLSSEMETVKKSADIEYGVLTNGVLKLYQGIRSIKELLILSEESGCSGDQWMKFE TKMGRFLETAGEEIVRIKAQESSTLSALEEVTELFHGGSFKEEGHTLRVFMAVRDFLSTL DEVCNDMGERFSA
BrCAGP35	*Bra039436*	FH2	MVYFRQIFLMIIVVAISLQCCSISADERYRTHIGKLVGEDGGEKKKLTVLEKFRALLDLIKP STPRRRNLAASPWPAPSPSPFPNGGPVESPAYSPAPQRPIPPHLRRPLPQRTHPPRQHEIQR RKHEKGGVFVPLVVSTACGIGFVVCVVVVLCLCSRVRKKNGKTLSFKRKQRKSQKVSIN PTLDFLYLNSVGVDLERQSSVLVKETENDDELREEEVKRSVETEILLDSDNAGSYSTKEI VSVQEKTEHSVSASEDDEESFHSVGGGSQYSNPRLSNASSSSVSGIGSSSQRFSERELDIP ECSGISHPPPPPPPPPPPQLLQFSNRGLLHTLSSPETAKPQTFSSQLSAKVSASSSKPLPPPPP PPPLPPSLQQQPQVMNKTPPPPLSLDFTQRTPLGKDGAPLPKLKPLHWDKVRATPDRTM VWDKIRTSSFEFDEEMIESLFGYTMQTSTKNEEGKCKTPSPGKHLLEPKRLQNFTILLKA LNATADQICSALGKGEGLCLQQLEALVKMVPTKEEELKLCSYKGAVDELGSAEKFLRAL VGVPFAFQRAEAMLYRETFEDEVVHLRNSFSMLEEACKELKSSRLFLKLLEAVLKTGNR MNVGTIRGGAKAFKLDALLKLSDVKGTDGKTTLLHFVVQEISRSEGIRVSDSIMGRIMN QRPNKNKTAEEKEEDYRRMGLDLVSGLNTELRNVKKTATIDLEGLVSSVSNLRDGLEQL RCLASSEENRAFVSSMSSFLRYGEKSLEELREDEKRIMERVGEIAEYFHGDVRGDDKNPL

			RIFVIVRDFLGMLDQVCRELRCVRVPNSPSPLAPFR
BrCAGP36	Bra001148	FH2	MVYFRQILLMIIVLVISLQCCNVFADTKELGEWTALMVANGERYRTQIGKLAGEDGGEKKKFPVLEKFRALLDLIKPSTLRRRSLATSASLAPWSAPSPSPFPNGGPAESPAYSPAPQRPIPPHLRRPLPHRTHHPSRQHELTRRKHENRGAFIHVVVSAACGIGFVVCVVGAFFLYARRRKKNGKTPPFQSSTRKVSINPSHSLGDDLEKQSSVSVKEVRETEKDVNDNKGVLLVEDVKTSAETEILSDSDNASSFSTKEIVSVHENDEEQQTVDSVSGPVVSNGCDSSDDNESFHSVGGGSSVGSSQRFSEREILPPPPPPPPPPPLPPGLHTLSLPKTTTLSSQLTAKVCASSSDSTLPSTPPPLRPPPPPPPPSQQPQATNKTPPPPLSLDFSQRRPLGKNGAPLPKLKPLHWDKVRATPNRTMVWDRLRASSFEFDEEMIESLFGYTMQSSTKNEEGKCKTPSPGKHLLEPKRLQNFTILLKALNANADQICSALGKGLRLQQLEALVKMVPSKEEELKLCSYKGEVDELGSAEKFLRAVVGVPFAFQRAEAMLYRETFEDEVVHLRNSFSMLEEACKELKSSRLFLKLLEAVLKTGNRMNVGTIRGGAKAFKLDALLKLSDVKGTDGKTTLLHFVVQEISRSEGIRVSDSIMGRIMNQRTNENRTAEEIDEDHRRMGLDLVSGLNTELRNVKKTATIDMEGLVSSVSNLRDGLGGLKCLASEKLKGDEENRAFVSSMSSFLRYGERSLEELREDEKRVMGRVGEIAEYFHGDVRGDEKNPLRIFVIVRDFLGMLDLVCRELRCVRVPNSPSPLAPFR
BrCAGP37	*Bra037087*	FH2	MGRLTKAFLAIFLVVLVCVSVEIIARDGANRLRSSVSEEAHGVDMAEKTKIRCRKELKDENKDCLVYISREAAANGYVKLSVLTGYGHWFGPLLDSTPRRKLISKKKKFTVSAPNFALGPAPRLTPGPAPSTSPSQPSSHSPPDESNSAPVKRKPSVVAPSPSVVLSPAKKYDILMQLIIAVASTAVLTFFLVTLLFLCCFRRNRSPRDGPRDEGQFLHLVDLSPGSNETSPAAANPSRRFFSASSKKKSFLSRMSLKRSGHDQFSTAQASTSSGHPPPLKLPPGRTAAPPPPPPPPAAPAPPPPQPPPPPKSKPPPPPKLVRPPPAPPKGAAGKRQGHHSSSGDASDVDSETGAPKTKLKPFFW

			DKMANPDQKMVWHEISAGSFQFNEEAMESLFGYNDGNKNKSGQRGESSRDSHVQYIQIIDPRKAQNLSILLRALNVTTEEVVEAIKEGNELPVELLQTLLKMAPTTEEELKLRLYSGDVNLLGPAERFLKILVDIPFAFKRIESLLFMISLQEEVSGIKESLSTLEVACKKLRNSRLFLKLLEAVLKTGNRMNVGTFRGDAQAFKLDTLLKLSDVKGTDGKTTLLNFVVLEIIRSEGVRALRLQSKSFSSVRTEEDTNNNNTDSSPQSVERYRSTGLQVVSGLTTELEDVKRAAVIDADGLAATLTNLSDSLTNAREFLKSMEEESDFEKALAGFIERADADIKWLKEEEERIMALVKSSADYFHGKSAKNEGLRLFAIVRDFLIMLEKVCRHVKETTTSATKTRTHSGKKETQVTMQEDSHQPSTDNIQQRLFPAIAERRDDSSDDSDDE
BrCAGP38	*Bra004786*	FH2	MSRIPSLLRFATILFLFSSSSSSADQRLHSRHLLHQPFFPIPTAAPPPYQPPPPSSEPPSPSPRSHHHHKNHPETPPPHEKHPFSSASNPPPPPPPPPSPPHPNTFFPSSDPSSPTSHPPPSPSPPHRAPVPTFPANISSLVFPTHNKPHNSNPHVAKLLAITIAVVSAAILLSLLAVATIFLRRRRRRASPAKSTKTDARHLFNAAPSDVPLKHKQPPKYTSSNTSSEFLYLGTLVNSGLVDQQKSPGSVAGVLELPPAQGSSSSTPSYSQYQKLGSPELRPLPPLPKLPVYTPNYRSTELLNPKGQDFDGDDNENDEFYSPRGSPVRKQSPPRVKNSGSPGTSLKPPKSSLSPRLSLNSSNGSVSKKPVPTRPPPPPPPQIHVLPVTNSVSEEKDETFKPKLKPLHWDKVRASSSRVTVWDQIKSNSFQVNEEMIETLFRANDPTSRTKESVTTDVVQSTSHQFLDPRKSHNIAILLRALNVTADEVCEALVEGNSDALGSELLQCLLKMAPTKEEEDKLKELKEDDDDESSPSKTGPAERFLRALLNIPLAFKRINAMLYIVNFESETEYLKRSFHTLEAACGELRNTRMFLKLLEAVLKTGNRMNIGTNRGDAHAFKLDTLLKLVDIKGADGKTTLLHFVVQEIIKSEGARVSTTPTQSPVGVNMAEQSAFQDDLELKKLGLQVVSTLSSQLTNVKKAAAMDSTSLSDEAAELSKGITKVKEVIMELKQETGVERFLDSMNSFLNKAEKEITEVQSHGDSVMKMVKEVTEYFHGNSESHPSRIFTVVRDFLTVLDQVCKEVGRVNERTVYGSVPRASVSNQVVTPLFPVANNDNNSEKSHSGSL

			GNQEDEDLF
BrCAGP39	*Bra000328*	FH2	MSKIPFLLRFAVIVFFIFSSYSSGDQRLNSRHLLHQPFYPIVTAAPPPLSSKPPSPSPDKQPPP PPPPPPLSSNPPSPSPDKPHHHKKHPAAAPPPQDKHLFSSVTPPPPPPAPHSNPFFPSNAVHP SPPPPPPASIPTFPANISSLFFPPHNSSKPHTNNHHVAKLVSITVSVVSSSVLLSLLVVLILYL RRTRRRRRLPAYNTKSTRSDSLQLFNASPSDGARKHKQPHKHTSSNTSSDFLYLGTLVNS RSEGFAPQKSPVSRNVAGVLELPPVPASSSSSSSYSRNQRPGSPELRPLPPLPKLPAFSPTY LSPEHLFPKRQDFDGDDDEFFSPRGSSGRKQSPPRVVEDSGVVQSVNDSNSCSPTSFNDS PATSLKPTSLSPLSLHSETSSNGSVLKKTGPARPPPPPPPPPPPPQFSEVPAATSPSPPSDPDK KEETLKPKLKALHWDKVRASSSRVMVWDQIKSNSFQVNEEMIETLFRANDPSSRARDV GNAGVVQSANQENQFLDPRKSHNIEILLRALNVTADEVCEALVEGNADMLGPELLECLL KMAPTKEEEDKLKELKDDDESTTSKIGPAEKFLRALLNIPLAFKRIDAMLYIVNFDSETE YLKRSFHTLEIACGELKNTRMFLKLLEAVLKTGNRMNIGTNRGDAHAFKLDTLLKLVDI KGADGKTTLLHFVVQEIIKSEGARVSSTPIQSPIGDDDIAEQSAFQDEIELKKLGLQVVSG LSSQLINVKKAAAMDSTSLSKEIAEISSGITKVKEVIMELKQETGVERFLESMNSFLDKGE KVITEIQSHGINVMKMVKEVTEYFHGNLESHPFRIFTVVRDFLTILDQVCKEVGRVNERT VYGSRPSPSNQTVTPLFPVVNINHAELSHDDDSFQVITIG
BrCAGP40	*Bra029012*	FH2	MILGGGMCNQNWARLVLLLVLLSGFFLVIALAESSEKDETFISQFMAPSTEQANEQMVET LWAHRCWQDPDCVKEAVTVFNLCFPGSKDNNLELFGFTPSHLKQTLLMCIQKQGELNG HNLNYLKLLPSILDNAPRRNLASTPSPSPPPKRSSRRPPPAASKKSVSEKLTSKPAPAAKG KEDHQKTIIIAVVTTAVSTFLLAACLFLCCTKVCGKGSGGRINDERPLLSLSSNEYSLGSS NNYGGSGKGHQSFNGNNSDNFVTLEERMSMDGMFNNINNSHGIPPLKAPPGRKSSKIS

			WKPPSGKVEPLPPEPPKFLKVSSSKKSSSAPSPSPPPPPMPSSAGPPRPPPPAPPPGSGGPKPPPPPGPKGPRPPPPMSLGKKAPPASPGPASSGDDDAPKTKLKPFFWDKVQANPEQSMVWNDIRSGSFQFNEEMIESLFGYAAADKNKNDKKGGSGQAALPQFIQILEPKKGQNLSILLRALNATTEEVCDALLEGNELPVEFIQTLLKMAPTPEEELKLRLYSGEIAQLGTAERFLKAVVDIPFAFKRLEALLFMCTLHEEMAFVKESFQTLEVACQELRGSRLFLKLLEAVLKTGNRMNDGTFRGAAQAFKLDTLLKLADVKGTDGKTTLLHFVVQEIIRTEGRRAARTIRESQSFSSVKTEDLMAEETSEEMEENYRGLGLQKVSGLSSELEHVKKSANVDADSLTGTVLKMGHALAKAREFVNSEMKSSDEESGFREALEDFIQNAEGSVVEILEEEKRIMALVKATGDYFHGKAGKDEGLRLFVIVRDFLIILDKSCKEVREAKGKQVKMARKQGSTASSVASEIPRAPSLDPREKLFPAITERRVDQSSSDSD
BrCAGP41	*Bra019092*	Glycerophosphoryl diester phosphodiesterase domain (GDPD)	MRGLLLLCGVVLIQLLAAQTDAQGSKSKWQTLTGFSPRVIARGGFSGLFPDSSIDAYNFAMLTSVEDVVLWCDLQLTKDGAGICFPGLTMSNASNIEAAYPNRTNTYLVNGVSTQGWFTIDFSLKDLNKVNLIRGILSRSERFDGNGYSILTVQDVNTELKPQGLWLNVQHEAFYAQHNLSMTTFLTTASKTVIIDFISSPEVNFFKKIAGRFGRILVPKSYVLPLDDKQYLLPSTSLVQDAHKAGLEVFVSGFANDVDIAHDYSYDPVSEYLSFVDNGNFSVDGVLSDFPISASASVECFSHMGRNATKQVDFLVISKNGASGDYPGCTDLAYAKAIKDGADVIDCSVQMSSDGTPFCSSSIDLGKTTMVAQTPLRNRSTNVPEISSLGGIYTFSLTWPEIQTLTPAISNPYRTYNMFRNPNERNAGKLVSLSDFLNLAKNSTSLSGVLISVENAAYLRENQGLDVVKAVLDTLTETGYTNITTKKVMIQSTNSSVLLDFKKQSRYETVYKVEETIRDILDSAIEDIKRFASAVVIVKSSVFPDSEGFVTGQTNVVERLQKSQLPVYVELFQNEFVSQPYDFFSDANVEINSYVTGAGVNGTVTEFPFTAARYRRNRCLGSKETPPYMAPVQPGGLLQVVNHASLPPAEAPNPVFTDAD

			VTEPPLPPVTAKAPTSTTPGTPSKPSTNAPAPSGQTRLTLSLLLSVFPMAIASLLLL
BrCAGP42	*Bra026409*	GDPD	MRGLRASSLLLCGVVLIQLLAVQIDAQRPKTQWKTLSGFSPRVIARGGFSGLFPDSSLDA<br>YNFAMQTSVLDAVLWCDLQLTKDGAGICFPDLTMSNASNVESVYPKGQSTYPVNGVPT<br>PGWFTIDLSLRALTNVSLIRGILSRSDKFDGNGYTISTVQTVNKEMKPQGFWLNVQHDA<br>FYAQHNLSMSNFLVSVTKTVIVDFISSPEVNFFRKIAGRFGREGPSFVFRFLEKEQFEPTTN<br>RTYGSILSNLTFVKTFASGILVPKSYVLPLDDKQYLLPSTSLVQDAHKAGLEVFVSGFAND<br>VDIAHDYSFDPVSEYLSFMDNGNFSVDGVLSDFPITASASVECFSHVARNATKQVDFLVI<br>SKNGASGDYPGCTNLAYEKAINDGADVIDCSVQMSSDGKPFCSSSIDLGNTTMVAQSPL<br>RNRSTSVPEISSVNGIYTFTLTWPEIQTLTPAISNPYSRRFNMFRNPNERNSGKLLSLSEFL<br>NLAKNSTTLSGVLISVENAAYLREKQGLDVVKAVLDALTETGYSNRTTTKVMIQSTNSS<br>VLVDFKKQSQYETVYKVEETIRDILDSAIEDIKTFASAVVIGKSSVFPVVDGFVTGQTNVV<br>ERLQKSQLPVYVELFQNEFVSQPFDFFSDATVEINSYVTGPGINGTITEFPFTAARYRRNR<br>CLASKETLSYMAPVQPGGLLEVVSPGSLPPAEAPNPVFTDADVTEPPLPPVTAKAPTPTP<br>GTPSTTAPAPSGQTQLTLSLLLSVFAMVLASLLLM
BrCAGP43	Bra002904	GDPD	MLGSRASKFLLSALILIHLLPTQLLAQRSKSPWQTLTGSAPLIIARGGFSGLLPDSSVDAYS<br>IVSQTSVSGAVLWCDVQLTKDGVGICFPDVKMMNASSIQDAYPKRKNSYLVNGVPTQD<br>WFTIDFTLKDLKSVFLIRGILSRSDAFDNNQYAISTVQDIAMELKPKSFWLNVQHDAFYA<br>QHNLSISKFMLSLPKTVTINYLSSPEVTFLRSIGGRFGKAGPKFVFRFLEKDDVEVSTNQT<br>YGTLLGNLTFIKTFASGVLVPKSYIWPLEDQYLSPHTSFVQDAHKAGLEVYASGFANDFD<br>MAYNYSFDPLAEYLSFMDNGDFSVDGFLSDFPLTASSAVDCFSHLGSNASTQVDFLVISK<br>NGASGDYPGCTDLAYSKAIKDGADIIDCSVQMSLDGIPFCLNSVDLGESTNIVQSPFRNR

			SATVPEIAPLGGLYSFSLTWSEIQTLRPAITNPYNRDFNLFRNPKERSSGKLVSLSDFLNLA<br>KSSTSLAGVLISVENAAYLREKQGLDVVKAVLDTLTKAGYSNATTTTKKVMIQSTNSSV<br>LVDFKKQSRYETVYQVEETIRDILDSAIQDIKKFADAVVVRKNSVFPVSESFTTGQTNLVE<br>RLQRFQLPVYVELFRNEFVSQPWDFLSDATVEINSHVTGAGINGTITEFPLTAARYKRNK<br>CLTRKDLPPYMSPVQPAGLLSIMSPTSLPPAEAPNPVFTDADVTEPPLPPVIAKAPTSSPGP<br>LSTDEKAPNGQTRVTLSLLLSAFAMVLASLLLL
BrCAGP44	Bra028979	GDPD	MITSYMQDNPSMFVSRASKFLLSVLVLIQLLPTQLLAQRSKSPWQTLTGEAPLVIARGGF<br>SGLFPDSSFNAYSFVASTSAPDAVLWCDVQLTKDGVGICFPYVTMYNDSNVQEAYPKKK<br>NSYLLNGVPTQDWFTVDFTSRDLNTVFLTRGVLSRSNAFDNTQNVISTVQEVASEFKPA<br>GFWLNVQHDAFYTQHNLSMSSFLLTVSKTVIIDYLSSPEVSFFRNIGGRFGKTGPKFVFR<br>FLDKDDVEVSTNQTYGSLMKNLTFIKTFASGVLVPKSYIWPVKDQYLLPHTSFVRDAHT<br>AGLQVYGSGFANDFDIAYNYSYDPLTEYLSFMDNGDFSVDGFLSDFPLTASSAIDCFSHL<br>GSNASTQVDFLVISKNGANGDYPGSTDLAYTKAIKDGADIIDCAVQMSSDGIPFCLNSTN<br>LGESMNIVQTPFRNRSTTVPEFNSLAGLYSFNLAWSEIQTLTPAISNPYSRNFHMFRNPRE<br>RSSGKLVSLSEFLNLANNSSSLVGVLINVEHAAYLREKQGLDVVKAVLDTLKESGYSNAT<br>KRVMIQSSNSSVLVDIKKQSRYETVYQVEETIRDIQDSAIQDIKKFADAVVVGRFSLYPISA<br>SFITGQTNLVERLQKFKLPVYVETFRNEFVSQAYDFLSDATVEINTHVTGAGVSGTITEFP<br>LTAARYKRNRCVARKDTPVYMIPVRPAGLLEIVSPTFLPPAEAPNPVITDADVTEPPLPPV<br>TAKAPTTSPGPLPTDKKAPNGQTRVTLSVFLSAFDVVLASLLLL
BrCAGP45	Bra035244	GDPD	MPTSTVLLCCVVLIQLFAGQTDARSSSPWQTLSGDAPLVIARGGFSGLFPDSSLNAYSFAV<br>QTSVPGSALWCDVQLTKDGAGICFPDLKLNNASTVEYVYPNRQKSYPVNGVSTQGWFT

			IDFSLRDLNDVSLIRGILSRTEKFDGIYPILTVEDVTTQIKPESFWLNVQHDAFFEQQNLSM SKFLISASRIVSINYISSPEVNFFTKISGRFGRNGPLFVFQFLRKEDIEPTTNQTYGSILSNLT FVKTFASGILVPKSYILPLDDKQYLVPPPKSLVQDAHKAGLQVYVSGFANDIDIAHDYSS DPVSEYLSFVDNGNFSVDGVLSDFPLTASSSIDCFSHLGQNAIKQVNFLVISKNGASGDYP GCTDLAYDKAIKDGADVIDCSVQMSSDGVPFCSRSIDLSNSTMISQTPFAQRSTLVPEISS SGGIYTFSLTWAEIRNLTPAIGNPYRSYTMFRNPNKKNSGKLILLSEFLNLANNSTLLSGV LISVENAVYLREKQGLDVVKAVLDTLTETGYGNGTTKTKVMIQSTNSSVLVEFKKQSKY EAVYKVDEKISDISDSAIEDIKKFANAVVIGKESVFSLLDSFITRQTNVVEKLQKSKLPVY VELFQNEFVSQPYDFFSDPTVEINSYILGAGINGTITEFPFTAARYKRNRCLGTQETLPPY MAPFKPGSLLQAVNAIPPAQAPNPVFTDDDVTEPPLPPVTAKSPTSSPGTSFTNAQAPRPS GQTRLTLSLRFSVFASLLLLWISSPLCS
BrCAGP46	Bra004101	GDPD	MGASRVLLCCVVLIQLFAGQADAQRSSSPWQTLSGDAPLVIARGGFSGLFPDSSFNAYDF AKQTSVAGAALWCDVQLTKDGAGICFPDLKLNNASTVEDVYPNRQKTYSVNGVSTQG WFTIDFSLRDLISNVKLNRGILSRSEKFNGIYAIMTVEDVTTQIKPESFWLNVQYDAFYAQ HNLSMSSFLISASTTVSIDYISSPEVNFFKKIAGRFGRNGPSFVFQFLGKEDFEPTTNRTYG SILSNLTFVKTFASGILVPKSYILPLNDKQYLLLPPASLVLDAHKAGLQVYVSGFVNDNDI AHDYSSDPVSEYLSFVDNGNFSVDGVLSDFPITASASIVDFLVISKNGASGDYPGCTDMA YDKAIKDGADVIDCSVQMSSDGKPFCAHSIDLSNITNIAQTPFTKRSTHVPEISSNDGMY TFSLTWSEIQTLKPAISNPYRVYNMFRNPNEKNSGKFILLSDFLNLAKTSTSLSGVLISVEK AVYLREKQGLDVVKAVLDTLTETGYSNGSTTKTKVMIQSTNSSVLVDFKKQSSKYETVY KVEEKIGDISDSAIEDIKKFANAVVIGKETVFPLFDDGFIIRQTNVVEKLHKSKLPVYVEVF QNEFVSQPFDFFSDPTVEINSYVSGAGIDGTITEFPFTAARYKRNRCLGTKESLAPPYMSP

			VRPGFFLNLVNAIPPAQAPNPVFTDDDVTEPPLPPVTAKSPTSSPGTSFTNAQAPRPSGQTRLTLSLRLSVFASLLLL
BrCAGP47	Bra034037	GDPD	MRASTVLLSSVVLIQLFAAQIDAKSSRSPWQTLSGDAPLVIARGGFSGLFPDSSSVAYKFAKQTSVADLFPNRQNFYPVNGVTSQGWFTIDFSLRDLKNASLIRGIFSRSEKFDGNGYSILTVQDVSMQIKPESFWLNIQHDAFYAQHNLSISSFLISASRTVSIDYISSPKLNFFSKVKGRFWRNEPSFVFQFLGKEEFEPTTKRTYGSILSNLTYVKTFASGILVPKSYILPLDDKQYLLPPTSLVQDAHKAGLKLYVSGFANDVDIAYNYSFDPVSEYLSFVNNGYFSVDGVLSDFPITASTSIDCFSHIGRNARKQMDFLVITKNGASGDYPGCTDLAYEKAIQDGADVIDCSVKMSSDGKPFCSSSIDLTQSTMVIQSPFRNRSTIIPDISSDPGIYTFNLTWPEIQSLTPAIMNPYRSYNMFRNPNEKNSGRIISLSEFLHLANKSTSIYGVLISVEYVVYLREKQGLDVVKAVLDTLTETGYSNGTSTTKVMIQSTHSSVLVDFKKKSKYETVYKVEETIRDISDSAIEDIKKFANAVVIGKSSVFPDVGSFVTGQTNVVERLRKSKLPVYVELFQNEFVSQPYDFCSDATVEINTYITGAGINGTITEFPLTASRYKRNQCLGRKETPSYMSPIKPGILLSLANPRSLPPAEAPYPVFTEDDVTESPQPPGTEKSPSSSTNAQTHRPSGQTRLTLSLRLSVFASLLLL
BrCAGP48	*Bra034311*	Protein kinase (Pkinase) and LRR	MAATSLVLTCFFSIFLLTHRVNSESLQEKQALLTFLQQIPHENRLQWNESDSACNWVGVECSSDRTSVHSLRLPGTGLVGQIPSGSLGKLSQLRVLSLRSNRLSGQIPPDFSNLTHLRSLYLQNNEFSGEFPASVTQLTGLVRLDISSNNLTGPIPFSVNNLTQLTGLFLGNNRFSGNLPSITVDLTDFNVSVNNLNGSIPTSLSKFPAASFAGNVNLCGGPLKPCKSFFVSPSPSPSSPDAPLSGKKSKLSTAAIIAIAVAGAVVGLLVLALLLFLCLRKRRRGKEGGTKAAETTATTRSVPSVPPAGGASSSKEVTGTSSGMGGETERNKLVFTEGGVFSFDLEDLLRASAEVLGKGSVGTSYKAVLEEGTTVVVKRLKDVAASKKEFESQMEVVGKIKHPNVFPLRAYYYSKDEKLLVFDF

			MPNGSLSALLHGSRGSGRTPLDWDNRMRIAITAARGLAHLHVSAKLVHGNIKASNILLHPNQDTCVSDYGLNQLFSNSSPPNRLAGYHAPEVLETRKVTFKSDVYSFGVLLLELLTGKSPNQASLGEEGIDLPRWVLSVVREEWTAEVFDVELMRYHNIEEEMVQLLQIAMACVSTVPDQRPVMQEVLRMIEDVNRSETTDEGLRQSSDDPSKGSEGQTPPGESRTPPRSVTP
BrCAGP49	Bra012045	Pkinase and LRR	MGAISLVLSCFVSILLLTDRVSSESPEEKQALLAFLQQTPHENRLQWNASDSACTWVGVECNSDRSSIYSLRLPGTGLVGQIPSGTLGKLTQLRVLSLRSNRLSGQIPPDFKNLTHLRSLYLQHNELSGEFPASITQLTGLVRLDISSNNLTGSIPFAVNNLTLLTGLFLGNNRFSGNLPSITVGLTDFNVSVNNLNGSIPSSLSKFPAASFAGNVNLCGGPLRPCKSFFISPSPSPDDAPSPSRLSGKKSKLSTAAIIAISVASALIGLLLLALVFFLCLRKRRRGGSRTKQTKPAETTTRNVPPEGIPPAGGASSSKDDLLRASAEVLGKGSVGTSYKAVLEEGTTVVVKRLKDVAASKKEFESQMEVVGKTKHPNVVPLRAYYYSKDEKLLVFDFMPSGSLSALLHGSRGSGRTPLDWDNRMRIAITAARGLAHLHVSAKLVHGNIKASNILLQPNQDTCVSDYGLNQLFSNSTPPNRLAGYHAPEVLETRKVTFKSDVYSFGVLLLELLTGKSPNQASLGEEGIDLPRWVLSVVREEWTAEVFDVELMRYHNIEEEMVQLLQIAMACVSTVPDQRPVMQEVLRMIEDVNRSETTDDGLRQSSDDPSKGSEGQTPPGESRTPPRSVTP
BrCAGP50	*Bra019320*	Pkinase and Gnk2-homologous (GNK2) domain	MSSCTSFIVLVLFSLLASYRAYAQNPHYTYHSCSNTTTYTRNSTYSTNLKTLLSSLSSPDASYSTGFQNATVGRDPDRVTGLFLCRGDVSPEVCRSCVAYSVNETLSRCPNEKEVVLYYEECMLRYSHRNILSTLVYEGGFFMFNGNISSNQEDRFEDLVSTTMNQAADKAANSSRKFYTIKANWTALQSLYGLVQCTPDLTRYDCLRCLHQSIDGIPLNRIGGRLFWPSCNARYELYLFFNENDTRTPLEQHAPPPPQPSPLPPPPASTSPVSSLTRTGKHHENSKVLIVAIVVGIVVAVLIFIAGYCFLAKRTKKTSDNAPAFYGDDITTIESLQLDYVIIQAATNNYSENNKIGEGGFGEV

			YKGTFANGVEVAVKRLSKSSRQGDTEFKNEVAVVAKLQHRNLVRLLGFSLEQKERILVY EYVPNKSLDYFLFDPAKHGQLNWTLRYKIIEGITRGILYLHQDSRLTIIHRDLKASNILLDR NMNPKVADFGMARIFGMDQTQDNTSKIVGTYGYMSPEYALHGQFSMKSDVYSFGVLV LEIISGMKNNSFYEIDGAHDLVTYAWKLWGNGTALNLVDPIIIDNCHNSEVVRCIHIGLLC VQEDPVDRPTFSTIFVMLTSDTVTLPVPRQPGFFVQSRPERDPLDSDQSTTTKSDPTSVDD ASITDIYSR
BrCAGP51	Bra019323	Pkinase and GNK2	MSSRASFIFLFLFLYSFLTSFRATAEIPIFVHHYCRNTTRYTANSIYFTNLKTLWSSLSSTNA SYSTGFQNATAGQARDMITGLFLCRGDVSLEVCRDCVSYSVKDIVRKCPNQREATIYYD QCMLRYSDRNIFSNVTLDDGFIMYNDHYDIPSAEQARFENLVLTTMIELAFEAANSSRHF CTRETKWNDYQDLYVLVQCTPDLTRQDCFRCLDRSINELYFSTIGERYLYPSCNSRYEFY KFYNETLLPPPPLAAPTPPVSSAPRPRDDITTIDSLQLDYRTIQAATNDYSENNKIGRGGFG EVYKGTFLNGTEVAVKRLSKSSGQGETEFKNEVVVVAKLQHRNLVKLMGFSLEREERIL VYEYVPNKSLDYFLFDPTKKGKLCWTRRYKIIEGVARGMLYLHQDSRLTIIHRDLKASNI LLDADMNPKIADFGMARIFGMDQTQENTNRIVGTYGYMSPEYAMHGQFSMKSDVYSF GVLVLEIMTGRKNSSFYERDGAHNLVTYAWRLWTNKTELDLVDPVIVYNCQKSEVVRCI HVGLLCVQEDPVERPTFSTILQMLTSNNVILPVPQQPGFVIQARPKRDLPDSDQSTMTKC ATRSVGDASVTDLYPR
BrCAGP52	Bra019322	Pkinase and GNK2	MCSWASLIFLFIFSFLTSFKASAQDPIYVYHICPNTTTYTRNSTYSTNLRTLLSSLSSSNSSY STGFQTAISGQGTDSVTGLFLCRGDVSPEVCRRCVAFVVNDTSTRCPNQREVVLYYDVC TVRYSNRNILSTLSTDGGVVLWNTQNITSNQKDQFRDLVLSTMNQAANEAADSRRKFD ARKANWTASQSLYGLVQCTPDLTRQDCLSCLQQSINQLPTDKIGGRFIMHSCGSRYELYA

			FYNESAITTPPLPPQPPVSTPPVSAPPPAGKGGSSSVLVVAIVVPIIVVVLLLIACYCFLAKRAKKTYGTASAFDGDDITTAESLQLDYRSIQTATNGFSESNKIGQGGFGEVYKGTLSDGTEVAVKRLSKSSGQGDAEFKNEVILVAKLQHRNLVRLLGFCLEGEERVLVYEYVPNESLDYFIFDLAKQTQLDWSRRYKIIGGIARGILYLHQDSRLTIIHRDLKASNVLLDSDMNPKIADFGMARIFGMNQTEENTSRIVGTYGYMSPEYAMHGQYSMKSDVYSFGVLVLEIISGKKNSSFYQTDGAHDLVSYAWRLWSNGTPLDLVDPIIVDNCQRNEVVRCVHIGLLCVQEDPVERPPFSTIVLMLSSNTVTLPVPRQPGLFFQSRLGKDPLDSDKFTTTKSLLRSVDDASITDVYPR
BrCAGP53	Bra019321	GNK2	MLRYSYRNILSTAIYNEGGVIRLNNNVSSNQEDRFQVLVSTTMNQAADKAANSSRKFYTIKANWTALETLYGLVQCTPDLSRYDCLRCLHQSIDGMPLNKIGGTLIWPSCNARYELYLFFNETGTGTPPEQQAPPPLQRLLPPPPASTSPASSLPRPGKHWNFKMVIVAIVVAIVVAVLLSIAGYCFLAKRTKKTSDNAPAFYGNDITTIESLQLDYRIIQAATNNYSENNKIGQGGFGEVYKCLGNQVFFVQSRSGRDPLDSDQSTTTKSDPTSVDDASITDIYSR
BrCAGP54	Bra019319	Pkinase and GNK2	MEPVIQSLEAEPVIQVTLSDFDNAWEALMRRVIAEATSSSSGSNTMYYGADRQQLGTSRSIYGFVQCSKDISPSKCEQCLRKNVDDYRSCCSGRQRGITERPSCFMRWDLDPFFGLFEDNIAPAPTPPPEKGDRKIPIGVVVGITGVLTFVISMLLSLGVALCIRRKAHQENGNESEQEFKNEVLLVAKLQHRNLVRLLGFSVEGAERILVYEFVPNKSLNYFLFDPVKRSQLDWRKRYNIITGIMLYLHQDSRLTVVHRDLKASNILLDVDMNPKIADFGLARNFRMNQTEANTGRVVGTVGYMPPEYVANGQFSMKSDVYSFGVLILEIIGGKKNSSFHKIDGSLRNLVTYVWRLWNNESLLELVDPAVGENYDKHEVTRCIHIGLLCVQENPADRPTMSTIFQMLTNTSITLHVPQPPGFFFRDGASPLAEGLTIGQSSIMSFACSVDDASITSVNPR

BrCAGP55	*Bra011048*	Pkinase and GNK2	MASTLLLASFAVFLTLTLTLPSPSTSTEVSRLDVLGWVCNNGSVPDQEAYRRSYQINIDKTRDDMRKLKFGTHEDGVPPQKMYFLSQCVSDLSPDECSLCWSRATDLLFNCFPSPGGRFYLDGCFVRADNYSFYREPVTRQDSKICGGDESSSSREFKGLVKEVTKSIVDTAPYSQGFSVVARKSVHGLTAYGLGICRQTLDEELCQLCLADGALSATSCSPATEAFVMNAGCYLRYSNYTFYNERELLSMSLTKEHVLRILVISMVCVLAIASGFWCGKCIYLGASSKKKLKEKESKSVSNNSNLMCFKYSTLEKATNNFNESCKLGVGGYGEVFKGTLSDGREIAIKRLHISGNKTREEIHNEIDVISRCQHKNLVRLIGCCFTNMNSFIIYEFLANSSLDHILFNPEKKKELEWKKRRAIILGTAEGLEYLHEACKIIHRDIKASNILLDLKYKPKISDFGLAKFYPEGGKDIPSSSPSPSPSPIAGTLGYMAPEYISKGILSNKIDAYSFGVLVLEITSGFRNNKFRSDNSLETLVTQVWKCYASDKMEEMIDKDMEEETDKTEVKRVMQIGLLCTQESPQLRPTMSKLVGDSIAIAVIRQNRNRLPACSLCNSSRNRITPSPTVSLHLLCRNPLGEIETVSPHLGEIETISLHLLSEHRPKRGTCSSSVV
BrCAGP56	*Bra032175*	Pkinase and LRR	MKTELVSPILFFFSFSCLLLVSSGLNSDGVLLMSFKYSVLLDPLSLLQSWSYDHDNPCSWRGVLCNNDFRVVTLSLPNSKLAGSIPSDLGFLQNLQSLDLSNNSLNGSLPVEFFAAGELRFLDLSNNLFSGEIPATVGDMHNLQTLNLSDNILAGKLPANLASLESLTEVSLKNNYFSGELPGGWRSVQFLDISSNLINGSLPPDFSGDSLRYLNVSYNQISGEVPPDVGDGFPRNATVDFSFNNLTGSIPDSPVFLNQKSISFSGNPGLCGAPTRNPCPIPSSPAAVTPTSTPALAAIPKTFGSNPESDSVGPENNKSNNRTGLRPGVIIGIIVGDIAGIGILALVFFYVYRYKKKNNMQKNTHSLEANEVKDTTSLSPSSSTTTSSSSPEQSNRFVKWSCLRKSQETDETEEEDEEEEEHQGPGETKKGTLVTIDGGEKELEVETLLKASVYILGATGSSIMYKTVLEDGTVLAVRRLGENGMSQQRRFKDLEAHVRAIGKLVHPNLVRLRGFYWGTDEKLVIYDFVPNGSLVNARYRKGGSSPCHLPWETRLKIAKGLARGLAYLHEKKHVHGHLKPSNILLGQDMEPKIGDFGLERLLA

			GDTSYNRASGSSRIFSSKRSASASTREFGPTPSPSPSSVGPVSPYCAPESLRNLKPNPKWDVFGFGVILLELLTGKIVSVDEVGIGNGLTVDDGNRALIMADVAIRSELEGKEDLLLGLFKLGYSCASQVPQKRPTMKDALVVFERFPMSSSAKSPSYRYGHY
BrCAGP57	*Bra019878*	Pkinase and LRR	MIFTSRQVFFVLSVLALTTMPFSAGVTNLRDVSAINNLYITLGAPSLHRWLAFGGDPCGEKWQGVVCDSSNITEISIRGMKVGGSLSDTLADFSSIQVMDFSDNHISGTIPQALPSTIRNLSLSSNRFTGNIPFTLSFLTELSELSLGNNVLSGEIPDYFQQLTKLTKLDLSANILEGRLPPSMGDLAALKILKDGTPFNTSIITPPPPAADPPPATHHAPPLPRVPPVSNVPPAPFAPLLPPPPPLVWSPPSDNVGGDPWNSGSGQPTLQISPPSGSGSGKFWSTQRIILVVSSVAIIVLVSGLCVTLWRCCRGKKYNRYGADARKDLQRPYFNKPPSQPTPTLGKVSREPMVKPYDGYGGGDRKYGYPMPPPRPEESRRAIPPASYYNKDVQKPLQQPPRRFQSNDDSAASKRAAHFPPGLNSSSSATVFTVASLQQYTNGFSEELIIGEGSLGNVYRAVFPHGKYLAVKKLSNTINKTQSDGEFLNLVSNVLKLKRGNILEFLGYCNEYGQRLLVYEYCPNGSLQDALHLDRKLHKKLTWNVRINIALGASKALQFLHEVCQPPVVHQNFKSSKVLLDEKLSVRVADSGLAYMLPPRPTSQVAGYAAPEVEYGSYTCQSDVYSLGVVMLELLTGRRPFDRTRPRGHQTLAQWAIPRLHDIDALTRMVDPSLHGAYPKKSLSRFADIISRSLQMEPGFRPPVSEIVQDLQHMI
BrCAGP58	Bra002639	Pkinase	MESPMRFNLRTAFSIIFLTFLPLNLKSQEVFDPSQDHSLIQSEASWNRRSLVETPPLPGKGPAVGASPPSPDQAFEGSTKPPPAPETQTPPGGDGTPSPPPSSVRTAQTPNPPSEPPPQLLSPPPRTKKTHNISMIVGIVVGVFTVSVALIIFFLIHTRKIPIKPWTNSGQLQNALITGNHTLFNVPRMQLSELRAACEDFSNIIGSFSDGTIYKGTLSTGAEIAVVSIAAGSRANWSTDMETQLLQKIRKLSKVDHKNFLNVIGYCHENEPFHRMLVFEYAPNGTLSEHLHSQHTEHLDWPTRLRIFMGIAYCLEHMHNLNPPILHTNLDSSCIYLTEDNAAKVSDFSVLNFISPSKESSSSKNLLE

			HSTLDPQTNVLNFGALVFEIITGRLPDPDSLFLEPKPARDLVDPTLKTFQEDVAERLLGVV RQCMNPYSAQRPTMRKVVVKLREIIGIEADAALPRLSPRWWSEMEIITTDGN
BrCAGP59	*Bra029710*	Pkinase	MEAVGRISPHGNVAPLRAYYFSKDEKLLVYDYYQGGNFSMLLHGNNEGGRGALDWEQ RLKICLGAAKGIAHIHSSSGAKLLHGNIKSPNVLLTQDLNACVSDYGIAPLMSHHTLLPS RSLGYRAPEAIETRKHTQKSDVYSFGVLLLEMLTGKAAGKTTGHEEVVDLPKWVQSVV REEWTGEVFDVELIKQQHNVEEEMVQMLQVAMACVSKHPDSRPSMEEVVNMMEEVR PSNGSGAGSGNRASSPEMIRSSDSPV
BrCAGP60	Bra040054	Pkinase and LRR N-terminal domain	MPLLKVIPPLSLSIYSIQRSASNATSQDSPMNPPQRSADIDSDKQALLEFASLVPHARKLN WNTTNPICTSWTGITCSKNNSRVTALRLPGSGLYGPLPDKTFEKLDALRIISLRSNNLQGN IPLTILSLPFIRSLYFHDNNFSGTIPPTLSPRLVNLDLSNNSLSGNIPASLRSLTQLTDLSLQN NSLTGPIPDLPPNLKYLNLSFNNLNGSVPSSLKSFPASSFQGNSLLCGSPLTPCPDNTTAPA KKVLSTAAIVGIAVGGSVLLFILLAVITLCCAKKRDDNGQDSTSTAPKAKTIRSDNKAEEF GSGVQEPEKNKLVFFEGSSYNFDLEDLLRASAEVLGKGSYGTTYKAILEEGTTVVVKRL REVAAGKREFEQKMEAVGRISPHVNVAPLRAYYFSKDEKLLVFDYYQGGNFSLLLHGNN EGGRAALDWETRLKICLGAAKGISHIHSASGAKLLHGNIKSPNVLLTQDLNACVSDYSIA PLMSHHTLLPSRSLGYRAPEAIETRKHTHKSDVYSFGVLLLEMLTGKAAGKTAGHEELV DLPKWVQSVVREEWTGEVFDVELIKQQHNVEEEMVQTLQIAMACVSRHPDSRPSMEE VVNMMEEIRASTGSGPGSANRASSPEMIRSSDSPV
BrCAGP61	*Bra013336*	Pkinase and LRR	MGCGFHYSYVFFLIITLQAPLSFSVNSQEYTLLKFRERVNSDPHGTLANWNISSDLCSWS GVTCVDGNVQILDLSGCSLGGTLAPEFNQLIELRSLILSKNHFFGEIPKEYESFSKLEFLDL RDNDLTGTIPPELTNILSLKHLLLSGNKFQSDMSIKILRMKLLHSPFAVLGCANRKLGHCIS

			RNHIIRVKKIEAFVFRIKATSRRFLKAFPSKFDFKRRELLEETSNLAAEPAPQAPSPSPETIT<br>EASPRSSGSFPAVTNAKKRIPPLVPPPPSPDENTSSDSSKNQPQDNKQSKGSKHVWLYVVI<br>AVASFLGLLIIVAVIFLCRKRAVKSIGPWKTGLSGQLQKAFVTGVPKLNRAELETACEDFS<br>NIIETFDGYTVYKGTLSSGVEIAVASTAVCESKEWTRAMEMAYRRTIDALSRINHKNFVN<br>LIGYCEEDEPFNRMMVFEYAPNGTLFEHLHDKEMEHLDWSARMRIIMGTAYCLQHMHE<br>MNPPMAHSDFNSSEIYLTDDYAAKVSEIPFNLEARLNPKKHVSGDLEQASLLLPPEPETN<br>VHSFGLLMLEIISGKLSFSDEYGSIEQWASKHLENDDLGEMVDPSLKTFKEEELEVICYVI<br>RECLKPDQRHRPSMKDVAEQLKQVINITPEKATPRSSPLWWAELEILSSEAT
BrCAGP62	*Bra011747*	Pkinase and LRR	MKMQLITAFFFFFFLCFVLGSSGLSPDGLLLMNFKSSVLVDPLSLLQTWNYNHETPCSW<br>RGVSCNNDSKVINLSLPNSHLLGSIHSDLGSLRSLQSLDLSNNSFNGPLPVSLFNGTELRS<br>LDLSGNMISGEVPASIGDLHSLQTLNLSDNALAGKLPANLVTLRNLTAVSLRSNYFSGEIP<br>GGWRDVQFLDLSSNLINGSLPPDFGGASLRYLNVSFNQISGEIPPEIGANFPINATVDLSFN<br>NLTGSIPDSPVFLNQKSIFFSGNPGLCGDPCPISSSPSTISDADSPTSTPAIAAIPNTISSNPVT<br>NPTTQQTNRTPRTGLRPVVITGIVIGDIAGIGILAVIFLYIYRRKKNIANNNNDKQREETTD<br>TITLSPSSSSSSSPDESRRFTKWSCLRKDPETTPSDEESGYNADQRSRDSEGTLVTVDGEK<br>EMEIETLLKASAYILGARGSSIMYKAVLEDGTVYAVRRLGETGLTQRRFKDFESNIRAIGK<br>LVHPNLVRLRGFYWGIDEKLVIYDFVPNGSLVNPRYRKGGGASSPYHLPWETRLKIARGI<br>ARGLAYLHEKKHVHGNLKPSSVLLGHDTEPRIGDLGLERLLTGETSYSRAGGSSRIFGSK<br>RSRGSSLDFSSIGPTPSPSPSSLGPLSPYCAPESFRSLKPSPKWDVFGFGMILLELLTGKVLS<br>AEEVGLGIGLTVEDGHHALRMVDVTIRGELLGKEDFLLGCLKLGYNCASPIPQKRPTMK<br>ESLAVLERFTPSSDVVKSPSFHYMNH

BrCAGP63	Bra036670	Pkinase	MSCFSCFSSKNLENEGSSLPAPYRQTGSPNNQRERTREVVANNAPSQSNNIEAQGFSFRELATATNNFKLENLIGEGGFGRVYKGKLNKTGQVVAVKQLDRNGLQGQKEFLVEVLMLSLLHHSNLVNLIGYCADGDQRLLVYEYMPLGSVEDHLLDLEPDQKPLDWNSRIKIALGAAKGLEYLHDKANPPVIYRDLKSSNLLLDQDFDTKLSDFGLAKLGPTGDTLHVSSRVMGTYGYCAPEYQRTGHLTVKSDVYSFGVVLLELITGRRVIDTMRPSHEINLVTWAQPIFRDPTRFPQLADPLLRGEFPEKSLNQAVAVAAMCLNEEPMVRPLISDVVTALSFLGASSDFSNADSSHLQQNPSEIYHDAVQWDSSPR
BrCAGP64	*Bra011439*	Pkinase and LRR N-terminal domain	MERRLMKIARFFWLVLVFDLVLRTSGNAEGDALSALKNSLSDPNKVLQSWDATLVTPCTWFHVTCNSENSVTRVDLGNANLSGQLVTQLGQLPNLQYFFANTNLTPLPASPPPPISPTPPSPAGSNRITGAIAGGVAAGAALLFAVPAIALALWRRKKPQDHFFDVPAEEDPEVHLGQLKRFSLRELQVASDNFSNRNILGRGGFWKVYKGRLADGTLVAVKRLKEERTQGGELQFQTEVEMISMAVHRNLLRLRGFCMTPTERLLVYPYMANGSVASCLRDRPESQPPLDWPKRQRIALGSARGLAYLHDHCDPKIIHRDVKAANILLDEDFEAVVGDFGLAKLMDYKDTHVTTAVRGTIGHIAPEYLSTGKSSEKTDVFGYGVMLLELITGQRAFDLARLANDDDVMLLDWVKGLLKEKKLEALVDVDLQGNYIDEEVEQLIQVALLCTQSSPMERPKMSEVVRMLEGDGVAERWEEWQKEEMFRQDFSYQNYNQPNTAWLIGDSTSHIENDYPSGPR
BrCAGP65	*Bra037006*	Pkinase	MVFCYWWFRRLNNNSLSGEIPRSLTAVSSLQVLDLSNNPLTGDIPVNGSFSLFTPISFANTKLTPLPASPPPPLSPTTPSPAGSNRITGAIAGGVAAGAALLFAVPAIALALWRRKKPQEHFFDVPAEEDPEVHLGQLKRFSLRELQVASDNFSNKNILGRGGFCKVYKGRLADGTLVAVKRLKEERTQGGELQFQTEVEMISMAVHRNLLRLRGFCMTPTERLLVYPYMANGSVASCLRERPESQPPLDWPKRQRIALGSARGLAYLHDHCDPKIIHRDVKAANILLDEDFEAVVGDFGL

			AKLMDYKDTHVTTAVRGTIGHIAPEYLSTGKSSEKTDVFGYGVMLLELITGQRAFDLAR<br>LANDDDVMLLDWVKGLLKEKKLEALVDVDLQGNYIDEEVEKLIQVALLCTQSSPMERP<br>KMSEVVRMLEGDGLAERWEEWQKEEMFRQDFNYQNYNQPNTSWLIGDSTSHIENEYP<br>SGPR
BrCAGP66	*Bra034562*	Pkinase and LRR N-terminal domain	MRGMMKPSFLWLLLVFDLVLRVAGNAEGDALSALKNSLADPNKVLQSWDATLVTPCT<br>WFHVTCNSDNSVTRVDLGNANLSGQLVMQLGQLPNLQYLELYSNNITGTIPETLGNLTE<br>LVSLDLYLNNLSGPIPKSLGRLQKLRFFFANTNLTPLPASPPPPISPTPPSPGGSNRITGAIAG<br>GVAAGAALLFAVPAIALALWRRKTPQDHFFDVPAEEDPEVHLGQLKRFSLRELQVASDN<br>FSNRNILGRGGFGKVYKGRLADSTLVAIKRLKEERTQGGELQFQTEVEMISMAVHRNLL<br>RLRGFCMTPTERLLVYPYMANGSVASCLRDRPESQPALDWPKRQGIALGSARGLAYLHD<br>HCDPKIIHRDVKAANILLDEDFEAVVGDFGLAKLMDYKDTHVTTAVRGTIGHIAPEYLST<br>GKSSEKTDVFGYGVMLLELITGQRAFDLARLANDDDVMLLDWVKGLLKEKKLEALVD<br>VDLQGNYIDEEVEKLIQVALLCTQSSPMERPKMSEVVRMLEGDGLAERWEEWQKEEMF<br>RQDFNYQNYNQPNTSWLIGDSTSHIENDYPSGPR
BrCAGP67	Bra022410	Pkinase	MVLHKQALLLTFISVLGFHQLPSPTEAECPLDLTSSNFTLVASVCSTNADRAKCCRYMNA<br>FVAVSVSRYANHTADLGVAPELTSICITTISRTMELYGIPTNATLFCGLGTKILVSYDCEGLT<br>TVTQMLQSPKFGDVSRNCELPFRCKSCLNSGITYIRSLVDRGNNIKMSTCRDATYAALAS<br>RVDSSSALELASCFFNVSELTTTPEFPLSPEASPVPVVADSPSGNDDLVLSPRRSHHGYHL<br>TVVPAIGIAVTVFSVMMLAVLIVLIQRKKRELDDDDSEGKDHNPTKTLPKVMMIHEGSSL<br>AFRKFSYREIRKATKEFSSVIGSGGFGTVHRGEFSNGLVAAVKRMKRSSEQADDEFCREIE<br>LLARLHHRHLVALKGFCTKKNERFLVYEYMANGSLTDHLHSTEKPPLSWATRMKIAIDV

			ANALEYLHLYCDPPLCHRDIKSSNILLDDNFVAKLADFGLAHASRDGSVCFEPINTDIRGTPGYVDPEYVVTQELTEKSDVYSYGVVLLEIITGRRAVDEGRNRVEMSQPLLVSESRRVDLVDPRIKDCIDGEQLETVVAVVRWCTEKEGVARPSIKQVLRLLCESCDPLHMELAMAVEEHKGRSLRGGGDSGLASSSSTTSRSHCSRSFLLETGSPPNGLSF
BrCAGP68	*Bra031115*	Pkinase	MRNFAMHLLLFLLLHSPVCFARLFPFPFSRSKSHQMRFFHPHLYPSLAPAPSPALAPKPNIIPTPRHNKGGHYHHHRLVTSASPSSSHDCQQACVEPLTSSPLGSPCGCVFPMKVQLLLSVAPFSIFPVTSELEIEVAAGTYLEQSQVKIMGASADTENQGKTVVDINLVPLGEKFDSTTATLIYQRFRHKKVPLNESVFGDYEVTHISYPGIPSSSPNDDIVDGAPTESTRGLPLTANFANRSQGIDFRTIAIIVLSGFVLALVLAGAIFIVMQWNKVGMPSTAVGPALASSMKKTPGAGFMFSSSVRSSGSDSLMSCMAMCALSVKTFTLSELEKATDKFSAKRVLGEGGFGRVYQGSMEDGAEVAVKLLTRDNQNRDREFIAEVEMLSRLHHRNLVKLIGICIEGRTRCLIYELVHNGSVESHLHEGTLDWDARLKIALGAARGLAYLHEDSNPRVIHRDFKASNVLLEDDFTPKVSDFGLAREATEGSQHISTRVMGTFGYVAPEYAMTGHLLVKSDVYSYGVVLLELLTGRRPVDMSQPSGEENLVTWARPLLANREGLEQLVDPTLAGTYDFDDMAKVAAIASMCVHLEVSHRPFMGEVVQALKLIYNDADETCGGDYCSKKESSVPDSADFKGDLAPSDSSWWNLTPRLRYGQASTFITMDYSSGPHEEMENRPHSASSIPREGGLFLENRSGPLRPVRSRRNYFRSRGSMSEHGGPSSSRHLWSGNGDWF
BrCAGP69	Bra036461	Pkinase	MRNYAMLVLLLLLLHSLASFPVCFARLFPMSFPFTRSKSHQMRFFHPHLYPPASSPAFSPNPSHIPTTRHSAHHHQHRRWHLRPNVTAAPPPSNDCQQTCVEPLTSTPFGSPCGCVFPMKVQLLLSVAPFSIFPVTSELEIEVAAGTYLEQSQVKIMGASADSENQGKTVVDFNLVPLGEKFDNTTATLIYQRFRHKKVPLNESVFGDYEVTHISYPGIPSSSPYGDIVEGVPTASTDGLPVT

			ANVANKSQGIGFRTIAIIVLSGFVLTLILAGAIFIVRKWNKVGKSSTAVGPGLPPSMNKRLGARSMFSSSARSSGSDSLMSSMATCALSVKTFTLSELHKATDKFSAKRVLGEGGFGRVYHGSMEDGTEIAVKPLTRDNQNRDREFIAEVERLSRLHHRNLVKLIGICIEGRTRCLIYELVHNGSVESHLHEGTLDWDARLKIALGAARGLAYLHEDSNPRVIHRDFKASNVLLEDDFTPKVSDFGLAREATEGSEHISTRVMGTFGYVAPEYAMTGHLLVKSDVYSYGVVLLELLAGRKPVDMSQPSGEENLVTWARPLLANREGLEQLVDPRLAGTYDFDDMAKVAAIASMCVHQEVSHRPFMGEVVQALKLIYNDADETCGDYCSQKESSVPESAGDLAFSDSSWWNLTPRLRYGQASTFITMDYSSGPPEEMENRPHSVSSIPREGGLYLPNRSGPLRPVRTRRNFFRLRGSMSEHGGPSSSRHLWSGNGDWF
BrCAGP70	Bra036687	Pkinase	MRNFAMMLLLLLVHSLASFPLCFAARLFPMSLPFTRSKSHQIHFFHPRSNPSLAPAPSPALLPNQRHRGHHHHRRWHLRRNVTAVSPSSHDCQQTCVEPLTPTPFGSPCGCVFPMKVQLLLSVAPISIFPVISELEIEVAAGTYLEQSQVKIMGASADSENQGKTVVDINLVPLGDKFDKTTATLIYQRFRHKKVPLNESVFGDYEVTHISYPGTPSSPYGDIVEGVPSASAGGLPVTAIFANKSQGIGFRTIAIIVLSGFVLALVLAGAMFIVRKWNEVGRSSTAVGPALPPSVNKRLGGGSMFSSSARSSGSESLMSSMATCALSVKTFTLTELEKATDKFSAKRVLGEGGFGRVYQGNMEDGTEIAVKLLTRDNQNRDREFIAEVEMLSRLHHRNLVKLIGICIEGRTRCLVYELVHNGSIESHLHEGTLDWDARLKIALGAARGLAYLHEDSNPRVIHRDFKASNVLLEDDFTPKVSDFGLAREATEGSQHVSTRVMGTFGYVAPEYAMTGHLLVKSDVYSYGVVLLELLTGRKPVDMSQPSGEENLVTWARPLLANREGLEQLVDPTLAGTYDFDDMAKVAAIASMCVHQEVSHRPFMGEVVQALKLIYNDADETCGGDYCSQKESSVPDPADFKGDLAPSDSSWWNLTPRLRYGQGSSFITMDYSSGPLEEMENRPHSASSIPRGGMFLPNRSGPLRPVRSRRDFFRLRGS

			MSEHGGPSSSRHLWSGNGD
BrCAGP71	*Bra035635*	Pkinase	MEILMFLVRIYLVSSVLVAASSSGLDLLSPSSSPPPPLPETSKGFGEVPISSPESHKPGNAPPPKASLPSSPPLADVAAPPPSYSSGTKAPNREPIVSVPSAPGPVSSPVSDIPPFPSVALPQPTPSIVPPRNASNKKPVAPVASPPTISVDISPPVIPKLPHSRSPDSPTSTAPSPPKFNGHSHHTSSSPPLNHLHHQEPKKIKDSPPPPPPKMSNRPISSSMHPISIAPSPSPTQGLLPPLLKLFPTTHRQNLIAKLSFISPKAFPLRSSSKPRKLPPLQALPPPPPNSDCSSTVCLDPYTNTPPGSPCGCVWPIQVELRLTMPLYDFFPMVSEFAREISAGVFMKQSQVRIMGANAATEQPDKTILLIDLVPLGDKFDNMTAMLTYQRFYRKKVYIDATTFGQYEVVYVRYPGLPVSPPSGGMTVIDHEPFSRNNNNNGMVKKPFGVDVPKKMRRKEINGGSIAVIVLSAAAFIGLCFVVVWFLAFRRGRARRRLSTRASLPSLTKPPGSVRSLTGSRFSSTSLSFESSIAPLTLSAKTFTASEIVKATSNFAESRVLGEGGFGKVYEGLFDDGTKVAVKVLKRDDQQGGREFLAEVEMLSRLHHRNLVNLIGICIEDRNRSLVYELIPNGSVESHLHGVDKESLPLDWEARLKIALGAARGLAYLHEDSNPRVIHRDFKSSNILLEQDFTPKVSDFGLARNALDDEDNRHISTRVMGTFGYVAPEYAMTGHLLVKSDVYSYGVVLLELLTGRKPVDMSQPPGQENLVSWTRSFLTSREGLEAIIDQSLGQPEIPFDSIAKVAAIASMCVQPEVSHRPFMGEVVQALKLVCNECDEAKELNSVTSLTHDDLGDDNGAESSCGGEGSRRMVRYPLLPSYDSEPGTERGLSVSEMFTGSGRLERVSNSGPLASGGGKRFWQKMRRLSTGSLSEHGSSSLMVRSGSR
BrCAGP72	Bra002786	Pkinase	MEILMFLLRICLVSSVLVAASPSASGLDLLPPLSSPPSPLPEASKGFGQAPITSQPSLPPLPNVAAPPPSISPIGDVADPPPADSAGSKAPAGEPIVSVPNAPAPATIPVKDLPGKSPPVASPPRDAPKEPPFSGRVSPGPVSSPVSDIPPLPSVALPPPIPSVVPPNNASNSHKPIAPVASPPTDISPPVHPVIPKLPSSSPVPTSSPTRKSPITHPVFPIESPAAGSPDHPPPSDNGGENKSPAPSNEAAK

			PLPIFPHKASPPSVAPLAPKFNGHSHHTSPSTTPPPDTTPSNVHRTSSSAPPPPSYHRHHQE RTKITNSPASSPPPPPTHLISPKKPKRNGSVSPLPSPHHARSPPVPSLISPVHPPVSSSMHRIS IAPSPSPTQVLPLRSSSRPSKSRKFPLGPPLPAFPPPPPNSDCTSTVCLEPYTNTPPGSPCGC VWPIQVELRLSMALYDFFPMVSEFAREISAGVFMKQSQVRIMGANAASEQPDKSIVLIDL VPLGDKFDNMTAMLTYQRFWSKKVQIFGQYDVIYVRYPGLPASPPVSGMTVIDQGPYPG GDNNGRAMKPLGVDVPKKMRKKQLTGETVAVIVLSAAAFIGLCFVIVWFLVFRRRRDQ RVSKRAPLARPSLPSLTKPSGSARSLTGSRLSSTSLSFASSIAPFTLSAKTFTASEIVKATNNF AESRVLGEGGFGKVYEGLFDDGTKVAVKVLKRDDQQGGREFLAEVEMLSRLHHRNLV NLIGICIEDRNRSLVYELIPNGSVESHLHGVDKEASPLDWEARLKIALGAARGLAYLHED SSPRVIHRDFKSSNILLEHDFTPKVSDFGLARNALDDEDNRHISTRVMGTFGYVAPEYAM TGHLLVKSDVYSYGVVLLELLTGRKPVDMTQPPGQENLVSWTRSLLTSREGLEAIIDQSL GQPEIPFDSIAKVAAIASMCVQPEVSHRPFMGEVVQALKLVCNECDEAKELNSVTSLTQD ENRAESSCGGEGSGRMARYPLLPSYDSEPDTERGLSVSEMFTGSGRLERQSNSGPLASG RGKSFWQKMRRLSTGSLSEHGSASLMLRSGSR
BrCAGP73	*Bra021861*	LysM	MAARTLHALSTSPLFLLLLFFAASSPTKAQQPYVNNHQLDCENRDFDNITNGFTCNGPRS CRSYLTFWSLPPYNTPNSIATLLNASAAEIQTLNNLTSLTTVIPTRRLVVIPTTCSCSGGGG FYQHNATYRLSGERQETYFSIANDTYQALSTCQAMMSQNPYGERNLTAGLNLLVPLRCA CPTANQTAAGFRYLLTYLVAQGDSVSAIAEMFRSSTPAVSSGNELTSDNIYFFTPLLVPLRT EPTRIVITPPSPTPPVATPPQSPPVDPPGGSSSSHKWIYIGVGIGAGLLLLISILSLYFCYYKR RSKTSSLIEQNKLTDSSTKQSLPTTSSRSPLAVYKDTTSGDQTETDKSKPEFGSWLMLGG RAERNKENNALPGKWAAFKVPQKPIVRAAASSFEVFVDEKECTDERVQKKKKSETISSS

			SNVLPLNDGREIKK
BrCAGP74	*Bra008320*	LysM	MKNPEKPILLFLILASTLASTATSKSTIEPCSTSSTCNSFLGYTLYTDLKVTELASLFQADPVSILLSNSISTSYPDVENHVLPSHLFLKIPITCSCVDGIRKSTSTRYKTRTSDTLDSIAGSVYGGLVSPEQIQVANPDIESLDVGTSLVIPLPCACFNGTDESLPAVYLSYVVRGVDTMGGIARRFSTTVADLTNVNAMGAPDINPGDILAVPLLACGSNFPKYATDYGLIIPNGSYALTADHCVQCSCALGSRSMYCEPASLEVSCSSMQCRNSKFMLGNITSQETSAGCKLTTCTYNGFANGTILTTLSRSLQPRCPGPPQLAPLIAPPDTVPKELMFAPSPSPTPSPAPASDGVVSEGPSTVAAAPRGPTVASSSSIPGYPANGPAGSISIASCLTSYHSLVVSFISFASYSSVILV
BrCAGP75	Bra017956	LysM	MRNPENHILFLILASSLLFFTATSKSTIEPCSSNDTCNSLLGYTLYTDLKVSEVASLFQVDPISVLLANAIDISYPDVENHILPSKLFLKIPLTCSCVDGIRKSLSTRYKTRPSDTLGSIADSVYGGLVSAEQIQEANSVSDPSVLDVGTSLVVPLPCACFNGTDNSLPAVYLSYVVRGVDTLAGIARRYSTTVTDLMNVNAMGAPDVSSGDILAVPLSACASNFPKYASDFGLIVPNGSYALAAGHCVQCSCALGSRSLYCEPASLAVSCSSMQCRGSNLMLGNITVQQSSAGCNVTSCDYNGFDNGTILTMLSRSLQPRCPGPQQFAPLLAPPDTLPKDIMYAPAPSPDFDGPGSVASSPRSSIIPPGGGSFPGNPANGPAGSISMATASSVSHFFVIFLISISSFSFVFSS
BrCAGP76	Bra016402	LysM	MKTPDKPIFYLFLILASSSLFFTATTAKSTIEPCSSNDTCNSLLGYTLYTDLKVSEVASLFQVDPISILLANAIDISFPDVENHILPSHLFLKIPLTCSCVDGIRKSVSTRYKTRPSDTLASIAGSVYGGLVSAEQIQEANSVTDPSVLDVGTSLLVPLPCACFNGTDNSLPAVYLSYVVKGVDTLAGIARRYETTVTDLMNVNAMGAPDVSSGDILAVPLSACASNFPKYASDYGLIVPNGSYALAAGHCVQCSCALGSRSLYCEPASLAVSCSSMQCTNSNLMLGNITVQQSSAGCNVTTCDYNGFANGTILTMLTRSLQPRCPGPQQFAPLLAPPDTVPKDLMYAPAPSPDYDGPGSIAA

			SPGSSVIPPGGGSLPGNPANGPAGSISTAATVYSFSYFFIMFLISISFVFSC
BrCAGP77	Bra002362	La domain	MMAETEGSLADDREVIGGFETKSPWKTTASPVETVDAPVMGAHSWPALADAQQPRPK NLPTAAPPSKVIPTSIPAPAQGVAGQGKSKGGGKGNPAHKNLSGRHSKPGPKSNQSGPPP PPPPYVMHGVPYHPSPFPPMVPPPHATGPDFPYAPYPPYPVPGAPVAESGNEKKAQASPL PPVLPAPQGDHPGQPWQDQRGFGPRNMPHGAAGPRNFVRPPFMGQAPGFMVGPGSGFP GPVYYLPVPPPGAIRGYPLRYAPYPVNQADSSGNFLEYFSALSPSLLVNWVAPHLAMLTI CVFLPFFSDENLQNDKYLISLMDKQEGWVPIKIIADFKRVKMMTMDVEFIVYALGYSSS VEVQGEKIRRRDEWAKWVPASKRSDSEEKVGDNDGDSPESTTSRDNSEKQSNDTSKPTA CSSEGAQPSRTDANGSDILKSSSSEQRNMDDLSTDFSNTFLLDEEIDLEHKSPRRSGLSVC KRIEDEDDDIAVDDHDHDIQKLVIVTQNSGRSDGTGISGTKAKNIPKELASTINDGLYYFE QELKKNRPGRKKNNSHLDSRDGKVKGGGLNIKLGENSAANGGEEHSIRRKQNKGTHK NQMAHVRRFFSGNTRNHGAVSESPPSSSIGFFFGSTPPDSHGHRLSKLSSSPQYSLSGSSPP VGSLPKSFPHFQHPSHQLLEENGFKQEKYLKYRKRCLNERKKLGCGCSEEMNHLYRFW SYFLRETFVPSMYEDFQKFALEDAAGNYYYGLECLFRFYSYGLEKQFDEDLYKDFEQLT LDFYHKGNLYGLEKYWAFHHYRGQKEPIKKHPELEKLLKEEYRSLDDFRAKDSATSQK ENKSH
BrCAGP78	Bra020153	La	MAETERSLADDREMIGHVTGSETKSPWKTAPIEPADAPVMGAHSWPALADAQQPRPKN PPPPPPAAAAAKSIPTSIPNHSQAVVTGHAKSKAGGKANPAHKNPPGRYSKPGSKSNQSG PTPPAAAAYPMHAVPYHPPPFPPMSYPTGPDFPYPLYPPYPIPGAPAAESGSEKPVQASPLP PPPPQGEPRQHQRGFGPRNMPHGPAGGPRNFVRPPYMGQGPGFMVGPGPGFPGPVYYL PGPPPGAIRGYPPRFGPYPGNQGPQALSPEQLDLKDRHVTFRILIPFFSDENLQNDQYLISL

			MDEQGWVPIKIIADFKRVKMMTMDVEFIVYALRFSTSVEVQGDKIRKRDEWSKWVPAS KKSASEEKIGDNDKDSSESVTSKDNFKNSLKPTAFSSEGAQSSRTKSDNRKSLSDEQRKM DVLSSDFSNTFLLDEEMDLEHKSPRKSGLSMSKRIDDEDEDIAVDDHDIQKLVIVTQNSG RSDGTGISGTKAKNIPKELASTINDGLYYFEQELKKNRSGRRKNISHLDNRDGKVKAGG GLNTKLGENSAANGGSEEHSIRRKQSKGAHKHHTAHARRFFSGNMRNHGASLSSHTSE SPPSSSIGFFFGSTPPDNPGQRLSKLSSSPQCSLSGSSPPVGSLPKSFPPFQHPSHQLLEDNG FKQEKYLKYRKRCLNDRKKLGSGCSEEMNHLYRFWSYFLRETFVPSMYEDFQKFALED AAGNYNYGLECLFRFYSYGLEKQFEEDLYKDFEQLTLDFYHKGNLYGLEKYWAFHHYR GQKEPTIRKHPDLEKLLKEEYRSIADFRAKDPITSHKENKSH
BrCAGP79	*Bra011647*	La	MASAASSDSTSSSTSENRQSRRPSPWSQIVRGESDPPTISSSAAAPSSPQHKAPIDPVPSAS TAPLAPVAGDVRSEESGAQGNAGKKPVWKRPSNGASSEVGGPVMGASSWPALSETTKA PSSKSSSDSLKSLGDVASSSSVVVVTVSQGNSNASAPAPKQGGRANPNPTPYNSRQRSFK RNNSASGSTANGTVSQGPVVETPSHNPSPRGQNQRNGSASQPHGGGSDNFSPRDSHRNQ NGNHHHHQGHGGRRNQEHGNQNWNFSRSFNGRDGNAHSQRGAPAFVRHQPPTMQSI PPQFMAAQPIQPFGGPVPFPPELASPYYPRMPFIGPLSPGPVYYQVQDPPLNVKLQKQIQ YYFSEENLIRDTYLRGLMDDQGFVPLHVIAGFKKVAELTDSIQQIVEALQGSPFVEVQGD RIRKRYNWQHWLLPEDASLQFVNAVASGVRNLSIGQSSADPIGGPSSQLQPAGAENKAA SDGQQQFSVVNPVNNLNGSNGANR
BrCAGP80	Bra010506	La	MASAATNNSTSSPSLSPRHVSDYPRHVSESTRHVSSPTAAQSRRQVSSPWTQIVRGESEPP PTIAAAATSKPPIEPIASAAPPAALLTVEAAAGEDKSEGNAGKKPAWSRPSNGASEIGPVM GASSWPALSEATKAPSNKPSSDSSIGDVPSSVSQGAASASVPAPKQAGRANNPNPTPNHS

			RQRSFKRNGASGSTANGTASQPSAQGSLVEGTSHNPSPRGQNQKNGFASQPHGGADNQ RDSHRNQNGNHHHQNHGGRRNQEHGNQNWNFHRSFNGRDGNAQSPRGAPAFVRYAP PPPPPPVQAIPPQFMAAQSFGSPVPYPPELAPPFYPGMPFVAPLSPGPVFYHVQDPPLNIKL QNQIHYYFSEENLIKDTYLRDQMDDQGFAPLHVIAGFRKVAELTDSIQEIVEALQGSPFV EVQGDGIRKRHNWQLWLIPSPQSVDAVASRVGNLSIGQSSAEPIGGSGSQLQPPEAENKA VADGQPQSSGADPVSNRNGSGGANR
BrCAGP81	*Bra026880*	X8 domain	MRMFLGVLLLLALTTSSSAIYCLCKDGIGDNGLQTSIDYACGTLADCNPIHDKGACYQP NTIKNHCDWAVNSYFQKAAQVPGSCNFSGTATTSQTPPSNLVTGCIYPSSASSAGSPPSTT PPTGTTPTTNGTGGFTPFPGTPPAFGPTGTGGFTPSKAASSLVISSVFTLCFSSLAFLM
BrCAGP82	Bra026878	X8	MRMFLALFLLLALTTSSNATYCLCKDGTEDNALQASIDYVCGKLDCNPIRDKGACYQPD TIKSHCDWAVNSYFQSQAQAPGSCVFTGTATTSQNPPSNLVTGCVYPSSPSSPGCSPSTNG ASSLVISPAFAICLSTLAFLM
BrCAGP83	Bra026879	X8	MRMFLALFLLLALTTSSNATYCLCKDGTEDNALQASIDYVCGKLDCNPILDKGACYQPN TIKSHCDWAVNSYFQNVAQAPGSCDFSGTATTSQNPSPYLVTGCVYPSSASSPGSLPSTTP PPGTKQTNGTVTPTNGASVYQH
BrCAGP84	*Bra019700*	X8	MKVFLGLLLLLALTTPSSAIYCLCNDGIGEKELQTAIDYACGTLADCNPIQEKGPCYQPIT VKSHCDWAVNSYFQNAAQVSGSCNFSGTATTNLNPPSNLATGCIFPSSPSSAGTTPPTGPT PPTGPTPPTGPAPPAGPATPVGPTPPTPTNGTNTFPGAPLAPSPPGTGGFTPSNGASSLLISS VLTLCFSSLAFL

BrCAGP85	Bra016724	X8	MRVFLGLLLLLALTKSSSAIYCLCKDGVGEKELQTAIDYACGSLADCNPIHDNGPCYQPNNIKSHCDWAVNSYFQKASQVSGSCNFSGTATTNQNPPSNLVTGCIYPSSASTTSPTGTTLTNGTTPAFGPTGTGGFQGNGASSLVISHVLTHCFSSLVFLWGSDVRLGFSHA
BrCAGP86	Bra013621	Hydrophobic seed protein domain	MAPHCSTKTIVFVLALISIFFLSETEAQGRSPPRQPPAPRRPPPPRRPPPPLRPPPPPPFVCPPCVCPPPVFPPNIPPPEITPLEIQPPGVTPPETEPITPQPEIPPPEIQPPEITPPEISPPETEPKTPPPEITPLEIQPPGVTPPETEPITPLPEIPPPEIQPPEITPPEISPPETEPKTPPPEITPSQVPPPEITPPEIQPPKIEPPEITPPESPPPKISPPQIEPSEITPPEITPPEITPPEITPPEIPPPKISPPQIEPPEITSPEIPPPKISPPDTPPPSGTPPKQSPPLPPPNFQPPPPPLPTCPRNAAQQRACANVVRRYGNFLDFGRAQPCCSLIRDLSDREAAACLCGFVQPPGQRRSPPPRNIFVLCRACGRRVPRGFMCP
BrCAGP87	Bra016093	Ring finger domain and PA domain	MKMMMNRALVLLLLLLFHLLTLSSLASAKVILIRNNITRSFDDIEANFAPSVKAAGEIGLLYVAEPLDACSDLTNKPEQSSNGTSPFVLIVRGGCSFEDKVRKAQRAGFKAAIIHDNEDRGILIAMAGNSGGIKIHAVFVTKETGDALKEFAGLSDTKVWLLPSFENSAWSIMAVSFISLLAMSAVLATCFFVRRHRIRRRTSRSSRVREFHGMSRRLVKAMPSLIFSSVHEDNTTAFTCAICLEDYSVGDKLRLLPCRHKFHAVCVDSWLTSWRTFCPVCKRDARTANGEPPASESTPLLSSAASSFRSSSALSSFRSSAMLIGPSMGSLPTSISFSPAHASSSYIRQSFRSSLRRSPPISVSRSSMDLRQQGASPSPSQRSYMASPHSFNYPAMSPLNSRYMSPYRPSPSNASPGMIGSSSNHPLNPLRYSESAGTFSPYASANSLPDC
BrCAGP88	Bra036147	COBRA	MGFLLPILLGVFLLFTATPPSLSQFPPEIDPPAPAPISPSELCNGIFLSYTFILGRQIPPNDTTDQPYRFESVLTVLNNGREELKEWRVFVGFQHHEILTSASDAIIVNGTDLPAPVGNGTIFAGYPVSDLKTAIQTAGDLKQMTATIELVGTQFMVAPPAIPLPSNISLVNDGWSCPEPTATPLSKRQITTCCIRDPTFEVNTTTITDKFLPRQPGDLTIMYDVIRAYDQNYLAEVTMENHNPLGRL

			DHWELSFDWMREEFIQKMQGAYPTVVDATKCIFGPQSQIYTGLDFADVLTCERRPIIVDL PPTKAEDPVLGKIPSCCRNGTILPRTMDPSKSASIFTMQVAKMPPDFNRSALSPPQNWRIK GTLNPDYSCGPPVRVSPTLYPDPSGMPTNKTSFASWQIVCNITHAKTETPKCCVSFSAFFN DSIIPCNTCACGCVSETRRTCSAETPSLLIPPDALLVPFENRTSLTVAWNALKHKTIPNPMP CGDNCGVSINWHIATDYRGGWTARITIFNWGEIDFPDWFLAVQMKKPAIRGFEKAYSFN ASLLSIEGGVNNTIFMEGLPGLEYLVAERDELDPKKKLRVPGKQQSVIQFSKKLTPGINVP ERDGFPAKVIFNGEECLLPDVLPLPSGGRRNGFDTMVLLCMMIFVVALVI
BrCAGP89	*Bra010350*	PsaE	MAMMSASSVFLLPANVTAPAGASSSRNSVSFLPMRNAGSRLVVRAADEAAPEPAAPEGA PATTAAPAAAAATKPKPPPIGPKRGAKVKILRRESYWFKSVGSVVAVDQDPKTRYPVVVR FAKVNYANISTNNYALDEIEEVKA
BrCAGP90	Bra011057	PsaE	MAMMSASSAFVLTSNVTASAGVSSSRNSVSFLPMRNAGSRLVVRAAEDAAPETSSSEGA PATAVAPAAAAATKPKPPPIGPKRGSKVKILRRESYWFKNVGSVVAVDQDPKTRYPVVVR FAKVNYANISTNNYALDEIEELKA
BrCAGP91	*Bra037792*	Prolamin-like	MSPNTTSKRSLLTFLTLSYLLSTVHIITVAEARNMQTMTVAAEHSGSGNLVDCWNAALEL KSCTDEIVKFFMSRNGTAEPGVTGGIDKDCCGAIGLIGKECWSVMFTSLGLTTMEGNML REYCDFEAEKLVFSPSPSPAPEALALSPVEITYPGLD
BrCAGP92	*Bra001249*	DOMON	MASINSSLLLVLAVACFISPAISQTCSTQNVSTSFDSCMDLPVLDSYLHYTYDAANSSLSV AFVATPPRSGDWVAWGINPTGTRMIGSQAFLAYSPRAGARPMVDTYNISSYNLTPGRLTF DFWNVRAESMPGNLIVIYASVKVPMGANSVNQVWQIGGNVTNGRPGVHPMTPANMAS TRVLRLTGSDAPSSAPGSAPGSAPGSVPGSAQGPTTPGASTTPGQAGGPENAGSMSTSVN

			FGVNFGILVMLATIFIF
BrCAGP93	*Bra001259*	DOMON	MKLCSVSFISSLIALQLLPLFTIVNGQQATDSCNSTLPLKDLAFDSRHHQCVEVWRVQNY<br>ILRYARTVENTWSFILSAPDSSAYIGIGFSTTGQMVGSSAVVGWITSDSRSGSAKQYLLGG<br>KSPGEVIPDQGDLKIINGSLKIESVSSRLYLSFQLRAELPRERLLYARGPAEFFPSSPDFRLR<br>EHQFMTTTTINYNTGPATFGPSMSPGPGSSPPSPSSAYGLSPSLLFLFMGLVALKFY
BrCAGP94	*Bra006651*	Thioredoxin	MTNLTHSLLFFSCSLSLLIRVAIAGSRPAHGPAYSNPSAFSPEAYDFFHPKSSLPDNNPPRNS<br>HSLPFLSPSPSPSKASNVEADTQGSKVSSDERISESRREEGRGETVGIVIGISFTALLLMGIY<br>FVIKKRLANLTRTITVALKWRSAFSSASSPSQLFSFSPSFAASSSDVDDEDFSFLEDLNDEG<br>PGEPLTSPVSLSQTNSTEEKPEDPEAYDDVEYGDFSDLGFTDLVRKIHIFRPNISLIGGRQR<br>AVLRLKGDGVGLAKVDATEENELTHQYSVQGVPTIILFFVDGEHKPYTGGRTK
BrCHAE1	*Bra013339*	LRR	MKTKKMAQIYALFVLHFTFLFSTGLSHSYSLASSNSDLSDKEVHLIRQRQLLYYRDDFGD<br>RGENVVVDPSLVFENPRLRSAYVALQAWKQAILSDPNNFTTNWIGSDVCSYTGVYCAPA<br>PDNPRIRTVAGIDLNHADIAGYLPQELGLLTDLALFHVNSNRFCGTVPHRFNRLKLLFEL<br>DLSNNRFAGIFPAVILQLPSLKFLDLRFNEFEGPVPRELFSKDLDAIFINHNRFRFELPDNL<br>GDSPVSVIVVANNQFHGCIPTSLGDMKNLEEIIFMNNGFNSCLPSEIGRLKNVTVFDFSFN<br>ELVGSLPASTGGMVSLEQLNVAHNRFSGKIPASICQLPRLENFNFSYNFFTGEPPVCIGLPG<br>VDDRRNCIPARPAQRSPGQCAAFLSLPPVNCASFGCGRSVTPSPRPPVVVPSPPTTPSPGG<br>SPPSPSISPASPPMMVPPSPISPIPAPVSPSSPPSIGPSPPSTPPSPPGSPSPPGVVPFPPPSPVYS<br>PPSPPPSTGHSPPSPSPPTKFSPPSPPPSAGHPPPSPPPSTGPSPPPSPPSTGYSPPPPPPSTGYS<br>PPPPPPSTGYSPPSPPPPSTGYSPPSPPPSAGHCPPSPAPPTYSPSPPPPPPTYYPPQPPQPSPS<br>QPPQFSPPPTPYYYSSPPPPPHYWLPPPPHSPPPPVYHYPSPPPPPTPVYSPPPPCIDHSPPPP

			PPPTVHYSPPPSPVYYNSPPPPPSVHYSPPPSSPPPPVIHHSPPPPPPGYEGPLPPIPGVSYAS<br>PPPPPYY
BrCHAE2	*Bra002969*	FH2	<u>MGNQNRGGLLLWFILISGFLVISS</u>LEVNLDKDEPFLTPFVAPSTGMVNEPVVESSWAKSC<br>WQDSDCVKEAVAVFNLCLPASRELFGFKHSHLKQTLLGCIQEQAKLNGHNLKYLKLLPY<br>LLDTPRRNLASRPVSLSPSPSPSPPPKRSRVPPTRSRSPSPSNSFFPPSRSPPPAKKTASSSAK<br>RKEEHEKTIIIAVVSTAVSTFLLAALLFLCCTRVCGKGSGGRKNDERPLLSLSSSDHSVGSS<br>INYGGSIKGGNQSFNIYSNQGKMSSFDGSNSDTSDSLEERLSHEGMRTHGLPPLKPPPGR<br>TSSAHLGKPPSGKVEPLPHEPPKFLKVSSNKGSHHTQPPVPPPPMPSSAGPPRPPPPAPPPG<br>SGGPKPPPPPGPKGPPPPPGPKGPRPPPPMSLGPKAPRPSSGPAKSPSDDDGAPKTKLKPF<br>FWDKVQANPEHSMVWNDIRSGSFQFNEEMIESLFGYAAADKNKNDKKGAAGQAAPQF<br>VQILEPKKGQNLSILLRALNATTEEVCDALREGNELPVEFIQTLLKMAPTPEEELKLRLYC<br>GEIAQLGTAERFLKAVVDIPFAFKRLEALLFMCTLYEEIAFVKESFQTLEVACQELRGSRL<br>FLKLLEAVLKTGNRMNDGTFRGGAQAFKLDTLLKLADVKGTDGKTTLLQFVIQEIIRTE<br>GRRAARTIRESQSFSSVKTEDLMAEEASEEMEDSYRNLGLQKVSGLSSELEHVKKSANI<br>DADGLTGTVLKMGHALSKARDFVNSEMSSGEVSGFREALEDFIQNAEGSIVSILGEEKRI<br>MALVKSTGDYFHGKAGKDEGLRLFVIVRDFLIILDKICKEVKGKPVKMARKQGSTASAS<br>SETPRQAPSLDPKQKLFPAITERRMDQSSSDSD
BrCHAE3	Bra017734	La	MASSATSTNPNSSSSSAAQSRRPSPQVSSPWTLIVRGGDSVPTIAAAAAAPSSPQSKPPIEP<br>IADASPPPAAGEEKPEGNAGKKPVWKRPSNGGAAASEVGPVMGASSWPALSVAANKSS<br>SDSLKSLGDVAPSPPPVLVSQGIANASVPSSASKPAGRANPNPTPNNSRQRSFKRNGASGS<br>SANGTASQPSVQGSLAESPSHNPSPRGQNQRNGFPSHTHGGSGADNVSQRDSYRNQNG

			YHHQSHGGRRNQEHGNQNWTFSRSFNGRDGNAQSQRGAPPAFVRHPPPPPLQTIPPQF<br>MAAHPFASPLPFPPELASQYYQRMPFVAPLSPGPVFYHVQDPPLNIKLQKQIHYYFSEEN<br>LIKDTYLRRQMDDQGFVPLPVIAGFNKVAELTESVQQIAEALQGSPVVEVQGERIRKRYS<br>WQHWLIPQDPSSPQSVGAVASRVGNLALGESSAGPNGGSSSQLQPAGAENKAVSDGQQQ<br>SPGGVPGSNRNGSDGANR
BrCHAE4	Bra027650	GNK2	MVTFCNPSDNFTQTSSYQANRDLLLSSLRDSSSLGTYSNATVGRSPNKVHGMFLCRGDT<br>TAASCSDCVQTATVEIATNCSLNKAAVIYYEECMVRYANVSFFSVLEVRPSIVLYSLRSAP<br>NSDTFNETLADKFNQLILNVSSSSLVPYFVEDQERVTQAEGSYEFESLVQCSPGLDRFNCT<br>VCLRFALLRVSTCCGSPSSALIFTPKCLLRYQTSALSSPPPLSPSPPPPPPSPALFSPPPTLSQ<br>PPPPPLVFTRPQDVPSLSG<u>SFSNVIKGNKIFGRIVITMAALVFALVNL</u>
BrCHAE5	Bra020846	Hydrophobic seed protein	<u>MAPKGVTNTLVFVLALISILFIGQTKA</u>QGRPSPRLPPPPPLQRPSSPPPPLLVCPPCVCPILS<br>PPPPNTPPPTPQTSPITPVAPPPQTPPSNLPPAFPPNNPSATTPPQTSPTPPPQTPSVSPPITPTI<br>SPQLPPSNITPTPTEEILPVRPSVPPSQTPPVHSPIFSPKSPSILPPQVPSVPLPITPSETPSTIPP<br>QTPLQSPPQTPPTTPPQTPLAPPPNTPQPSSPPLNFQPPPPPPQTPPSPPPNSQPPSVPLPITPP<br>QTPPQSPPQTPPTTPPETPPVSPPNTPPLPSPPLNFHPPPPPPQTPPSSPPNSQPPSVPLPITPP<br>QTPPQSPPQTPPTTPPETPPVIPPNTPPLPSPPLNFQPPPPPPQTPPSPPPNSQPPPAPTCPRNA<br>SQLRACSNTIRRFGNFLDFGRAQPCCSLIRDLSDAEVAACLCGLVQPQSQRYSTPSRNIFII<br>CRACGRPMPRGFMCP
BrCHAE6	*Bra038210*	Root cap	<u>MDLAKHTTLQMLGFILLASLVLTMA</u>QPPGLTKPSHATCKIKKYKHCYNLEHVCPKFCPD<br>TCHVECASCKPICGPASPGDDGGDTPPTPVPPVSPPPPAPVPPVSPPPPVTPTPSYPTPTDPL<br>PPAPVSPPPPAPVPPVSPPPPTPTPYVPSPTPPVSPPPPSPTPDVPSPTPPSSPPPTTPTPAVPSP

			TPSSPPPPSPTPAVLTPPHVTPTPPTPAVPSPPDVTPTPPTPSVPSPDTPTAPLPPYSPPATPAPSVPSPTPTPPSSPTPPGSTPTTPTPSVPTPSPSVPVPSAPNSPPYVPPSSPTPTPPSDGEAGAGVRRARCKKKGSPCYGVEYSCPSACPRSCEVDCVTCKPLCNCDKPGSVCQDPRFIGGDGLTFYFHGKKDSNFCLISDPNLHINAHFIGKRRPGMARDFTWVQSIAVLFGTHRFYVGALKTATWDDSVDRISASFDGNVISLPQLDGATWTSSPGVYPQVSVKRVNADTNNIEVEVEGLLKITARVVSITMEDSRIHGYDVKEDDCLAHLDLGFKFQDLSDNVDGVLGQTYRPNYVSRVKIGVHMPVMGGDREFQTTGLFAPDCSAARFIGNGGRNGGWSKMELPEMSCASGVGGKGVVCKR
BrCHAE7	Bra022423	Root cap	MALATRVQILGCILLASLALTMADTPPGIAKNPSHATCKIKKYKHCYNLEHVCPKFCPDSCHVECASCKPICGPPSPGSDDDDGEDDGGYTPPAPVPPVSPPPPTPTPAVPSPTPPVSPPPPPPPTPTPAVPSPPPPVSPPPPPPPTPTPAGASPTPPVSPPPPSPTPAVPSPTPSSSPPPPTPTPAVPTPTPPTPSVPSPPGTPTAPVPPYSPPATPTPSIPSPTPTSPGSTPPYVPPSSPTPTPPSDGEAGAGVRRARCKKKGSPCYGVEYSCPADCPRSCEVDCVTCKPLCNCDKPGSVCQDPRFIGGDGLTFYFHGKKDSNFCLISDSNLHINAHFIGKRRPGMARDFTWVQSIAILFGPHRLYVGALKTSTWDDSVDRISASFDGHVISLPQLDGATWTSSSLGVYPQVSVKRVNADTNNLEVEVEGMLKITARVVPITVEDSRIHGYNVTEDDCLAHLDLGFKFQDLSDNVDGVLGQTYRSNYVSRVKIGVHMPVMGGDREFQTSGLFEPDCSAARFTGNRGSNGGRSKMELPEMSCASGVGGKGVVCKR
BrHAE1	*Bra030020*	-	MKSSIVLVAAAILCIVAFPTATVGKNLRFGLKPTQGWPHPSEASTNQMFMSTSQKFNYGDSKVWRCTYSNGSAPAISISISPPTPTMPSPSTPTTPSPSPPTPKTSPPPPTPSPPPPTSKKAPSPSPPPPPPTPSLPPPTPTKVPSPPPPTLSPPPPTPTKASSPPLPKPSLPPPTPKKTPSPPPPTPSLP

			PPTPKKSPSPSPPSDDESSSPSQPSNPPQEHHHHHEFPLEHIGRCYRNMGQVGFCRGQMAI SFYTRLFKVSKYCCNLIVNMKNECDDVIWGYFYDPHFVPLVRCTCHVSF
BrHAE2	*Bra009880*	-	MVSLNLSFALVFILAILFTFAEANYSRKLLQTPTNYQPAYSPPSPTPVYSPPVNPPPPTPVT YPPPTPAYPPPVALPPPAPINSPPPPAPIIPPLKANPSPQAYRAFYYRKSPPPPSGKPWWWLL
BrHAE3	*Bra014023*	-	MKSLIILIVAHFCIIVSPTTTMGGWPKPSEVSNEEKLVNTGQAQPHLYAGKFNFGDSKVW KCTYNNGSGVAISISYPSPPQPPSQKPPTPSSPPTPKMAPPLPKPSPPRPSPKKSPPPPKPSSP PPTPKKSPPPPKPSPPPPTPKKSPPPPKPSPPPTPKMSPPSPTPSPPRPTPKKSPPNPSSLTPNE SPPPAKTSILIIHSPPPPHEPIPAQSPPKEPTTPSTQWPPYRNWNPLGL

[a] Italics indicate a newly identified BrAGP that is highly similar to AGP-like amino acid sequences predicted in *Brassica rapa* by Ma et al. (2017). XXX, N-terminal signal peptides predicted by SignalP 5.0 Server; XXX, conserved domains predicted by HMMER; XXX, [Ala/Ser/Thr/Gly]-Pro; XXX, [Ala/Ser/Thr/Gly]-Pro-X(0,10)-[Ala/Ser/Thr/Gly]-Pro (two consecutive Pros are not separated by more than 11 amino acid residues); XXX, [A/S/T/G]-P_{2-4}; XXX, ≥ 2 SP_{3-5}; XXX, PPVK/T and KKPCPP; XXX, GPI-anchor addition signal predicted by the GPI-SOM or the BIG-PI Plant Predictor. The conserved residues (His, Cys, His and Gln/Met) involved in Cu binding and Cys residues involved in the disulfide linage are marked with Grey and dark blue in the amino acid sequences of the plastocyanin-like (PCNL) domains of the BrPLAs, respectively. The eight conserved cysteine residues were marked with dark green in the non-specific lipid transfer protein 2 (nsLTP2) domains of the BrXYLPs.
